# A Bis-Glycosylamine Strategy for the Synthesis of Dimeric Iminosugars Based on a DAB-1 Scaffold

**DOI:** 10.3390/molecules30020226

**Published:** 2025-01-08

**Authors:** Kamilia Ould Lamara, Nathan Noël, Fabien Massicot, Jean-Luc Vasse, Stéphane P. Vincent, Jean-Bernard Behr

**Affiliations:** 1 Université de Reims Champagne-Ardenne, CNRS, ICMR, 51097 Reims, France; kamilia.ouldlamara@gmail.com (K.O.L.); nathan.noel@univ-reims.fr (N.N.); fabien.massicot@univ-reims.fr (F.M.); jean-luc.vasse@univ-reims.fr (J.-L.V.); 2 NARILIS (Namur Research Institute for Life Sciences), UNamur, Rue de Bruxelles 61, 5000 Namur, Belgium; stephane.vincent@unamur.be

**Keywords:** iminosugars, multivalency, glycosylamines, glycosidases, inhibition, glycophanes

## Abstract

A straightforward synthetic route towards DAB-1 scaffolded dimeric iminosugars is described here, starting from readily available bis-glycosylamines. The method allows the integration of a variety of linkages (aryl, alkyl, polyethyleneglycol chains) between both iminosugars through the choice of the bis-amine used in the first step. Moreover, an additional substituent (allyl, ethynyl) may be inserted into the structure via nucleophilic addition of an organometallic reagent to the starting bis-glycosylamine. A symmetrical ethynyl-iminosugar proved susceptible to intramolecular Glaser coupling, affording the corresponding macrocyclic structure. Dimeric iminosugars were tested towards a series of commercial glycosidases to uncover potencies and selectivities when compared to DAB-1, their monomeric counterpart. Whereas a significant drop in inhibition potencies was observed towards glucosidases, some compounds displayed unexpected potent inhibition of β-galactosidase.

## 1. Introduction

**Scheme 1:**
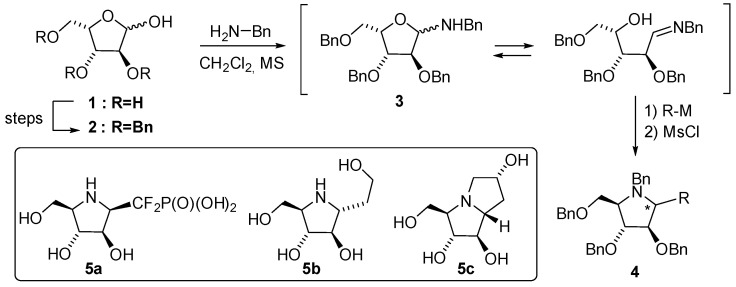
The glycosylamine strategy towards monomeric iminosugars [7].

*N*-glycosides are biomolecules, which originate from the condensation of carbohydrates with amines or related *N*-nucleophilic species [1]. Nucleosides or glycoproteins belong to this family, for which the newly formed anomeric bond is enzymatically processed in biological systems. In such cases, where the *N*-nucleophile is different from a primary amine, the anomeric bond is rather stable and tolerates the aqueous conditions of biological media. Glycosylamines resulting from reactions of sugars with primary amines, however, are much more susceptible to hydrolysis or decomposition [2,3]. Conjugation of carbohydrates with the amino function of aminoacids for instance occurs under heating and culminates in the so-called Maillard transformation after subsequent Amadori or Heyns rearrangements [4,5]. Nevertheless, for synthetic purposes, such glycosylamines might be prepared in non-aqueous media and are conventionally used without further purification in subsequent steps, leading to highly valuable products such as iminosugars, a family of nitrogen-containing carbohydrate analogues [6]. As a relevant example, tri-*O*-benzyl-l-xylofuranose **2** might be *N*-glycosylated with benzylamine in dichloromethane at room temperature in the presence of molecular sieves as a dehydrating agent to afford the expected anomeric amine **3** (Scheme 1) [7]. As observed with simple reducing carbohydrates, mutarotation in glycosylamines enforces an equilibrium between closed alpha/beta isomers as well as the open imine form. Reduction [8,9,10,11,12] or nucleophilic addition [13,14,15,16,17,18] is thus possible in order to functionalize further the glycosylamines into non-hydrolizable derivatives.

Aminosugar **3** for instance was the starting point for the synthesis of difluoromethylphosphono-iminosugar **5a** [19], designed as a potential inhibitor of chitin synthase. The diastereoselective addition of LiCF_2_P(O)(OEt)_2_ onto **3** followed by cyclization in the presence of methanesulfonylchloride afforded the expected iminosugar intermediate **4** (R = CF_2_P(O)(OEt)_2_). Instead, the use of allylmagnesium chloride in the key nucleophilic addition on glycosylamine **3** produced an allyl-pyrrolidine **4** (R = Allyl), which could be transformed in few steps into 6-deoxy-homo-DMDP **5b** (an alkaloid extracted from the bulbs of *Hyacinthus orientalis*) [20] as well as into 7-deoxy casuarine **5c** [7], a non-natural inhibitor of amyloglucosidase. Bis-glycosylamines, molecular tools that connect two carbohydrate moieties via a diamino linker, are rather seldom and have been used mainly in the elaboration of metal-coordinating carbohydrates. The examples from the literature focus on bis-glycosylamines prepared from protecting-group-free reducing sugars and diamino-alkyl, diamino-aryl, or diethylenetriamine as the nitrogenated counterpart [21,22,23,24,25,26,27,28]. Their complexing ability has been studied further in several reports but the use of bis-glycosylamines as intermediates towards the synthesis of more elaborated structures is, to the best of our knowledge, presently undescribed, notably for the synthesis of multivalent iminosugars.

**Figure 1:**
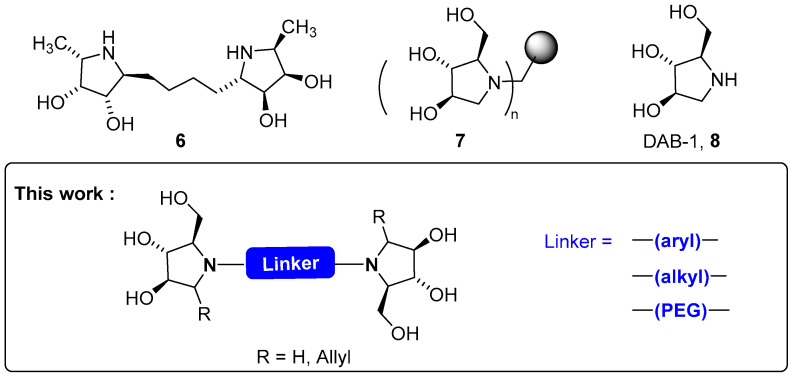
Structures of key compounds.

The presence of multiple copies of a given ligand gathered on a unique chemical structure, a phenomenon found in nature to improve host-guest interactions [29,30], is a recent strategy in pharmaceutical research to increase the performances of enzyme inhibitors via the so-called multivalent effect. During the last decade, a major step forward was achieved with multivalent glycosidase inhibitors, culminating in binding enhancement by several orders of magnitude [31,32,33,34,35,36]. Homo-dimeric compounds are the most simple of the series [37,38]. Although they generally display a modest but measurable multivalent effect, dimeric iminosugars remain attractive for mechanistic investigations. For instance, striking results were obtained with dimer **6**: kinetic analysis showed a 19-fold increase in the inhibition potency for **6** (IC_50_ = 0.108 µM) relative to the monomer towards fucosidase (Figure 1) [39]. Moreover, the use of stereoisomers of **6** allowed a detailed analysis of the mechanism of inhibition of such a dimeric structure, at a molecular level. In the same manner, multivalent architectures with general structure **7** based on *N*-tethered pyrrolidine **8**, a natural product named DAB-1, afforded strong inhibitors of α-mannosidase, *N*-acetylgalactosamine-6-sulfatase, and protein tyrosine phosphatase 1B [40,41,42,43,44]. Resting upon our experience acquired in the field of iminosugar synthesis by using glycosylamines as key intermediates, we aimed to come up with an extension of the method to prepare dimeric iminosugars based on a DAB-1 scaffold (Figure 1). The synthetic sequence exploits bis-glycosylamines as intermediates, which easily allows variation of the spacer (alkyl, aryl, PEG) separating both pyrrolidines by the crucial choice of the starting di-amine.

## 2. Results and Discussion

**Scheme 2:**
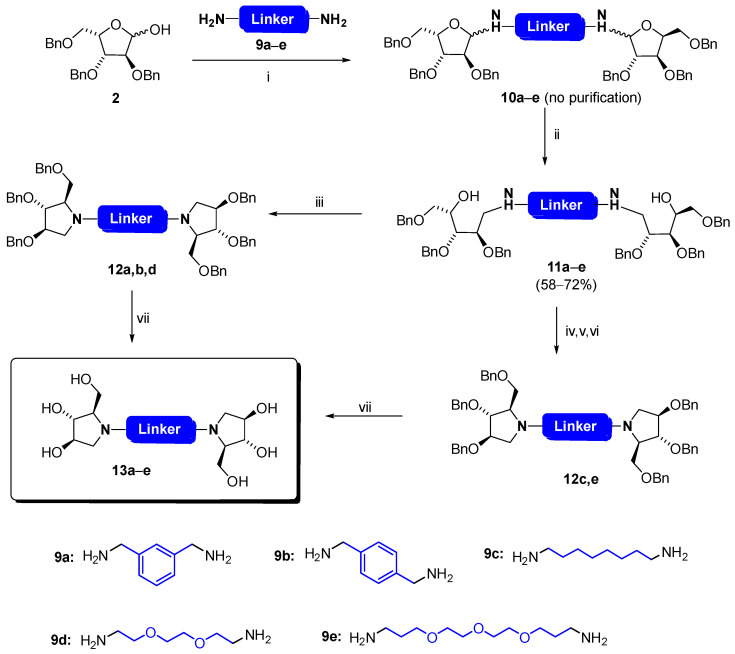
Synthesis of bis-iminosugars **13a**–**e**. Reagents and conditions: (i) **9a**–**e**, molecular sieves (4 Å), CH_2_Cl_2_, r.t., 23–48 h; (ii) LiAlH_4_, Et_2_O, 0 °C, 1 h; (iii) MsCl, pyridine, 0 °C, 2.5 h; (iv) Et_3_N, DMAP, Boc_2_O, MeOH, r.t., 1–12 h; (v) Et_3_N, MsCl, 0 °C then r.t., 40 min; (vi) conc. HCl, EtOH, 12 h at 60 °C (**11c**) or 29 h at r.t. (**11e**); (vii) BCl_3_, CH_2_Cl_2_, 0 °C, 2.5 h.

A critical aspect in the design of divalent glycosidase inhibitors is the chemical nature and the length of the linker which separates the two iminosugars. A variety of spacers have been devised in literature, which comprise alkyl, aromatic, polyamino, or PEG chains, each of them requiring specific chemistry for its introduction on the pre-formed pyrrolidine [35,45,46,47,48]. Here, the linker originates from the di-amine that is used to build the bis-glycosylamine, enabling a large variety of possibilities. Thus, in the first series of experiments, we evaluated the reactivity of a series of diamines **9a**–**e**, which feature various chain lengths and structures (Scheme 2). To this aim, each amine was stirred with tri-*O*-benzyl-l-xylofuranose **2** in dichloromethane in the presence of molecular sieves, following our own methodology for the synthesis of monomeric glycosylamines [7]. However, whereas an excess of amine could be used in the latter case as no competition occurs between mono and bis-glycosylation, the stoichiometry of the reaction has to be carefully controlled here in order to secure the formation of the symmetrical expected bis-glycosylamine by the introduction of the sub-stoichiometric amount of the amine. Fortunately, the reaction proved efficient, whatever the amine used, affording glycosylamines **10a**–**e** as the major compounds. Bis-glycosylation of the amine was confirmed by HRMS with the presence of peaks corresponding to *m*/*z* = [MH]^+^ and *m*/*z* = [MH_2_]^2+^/_2_. In some cases, a small amount of the monofunctionalized bis-amine (ie, tethering of only one sugar moiety) was identified in the Mass or NMR spectra. Nevertheless, the crude product was used as such in the next step since purification was not possible at this stage due to the lability of these adducts.

Next, we wished to use **10a**–**e** as intermediates for the preparation of dimeric iminosugars. The first option consists of reducing the hemiaminal to the corresponding amine, which could lead, after cyclization via an S_N_2 process and benzyl deprotection, to new dimeric DAB-1 models in a very straightforward manner. Although mild reducing agents such as NaBH_4_ or NaBH_3_CN react readily with simple glycosylamines [8,9,10,11], we found LiAlH_4_ as the most efficient hydride donor for our purpose. Treating bis-glycosylamines **10a**–**e** with an excess of LiAlH_4_ (4 equiv) in Et_2_O for 1 h afforded amines **11a**–**e** in acceptable yields. All compounds proved stable and were purified and fully characterized at this stage. A subsequent cyclization is required to reach the pyrrolidine core of DAB-1. This might be performed by activation of the free hydroxyl at C-4 of both sugar moieties, via mesylates or triflates for instance, followed by a spontaneous intramolecular attack of nitrogen to the generated electrophilic center. Contrasting results were obtained with compounds **11a**–**e** after treatment with methanesulfonyl chloride in pyridine, following a protocol used for monomeric analogues [7,19,20]. Whereas **11a**,**b**,**d** gave the expected bis-pyrrolidines **12a**,**b**,**d** in a single step, a mixture of several inseparable products was obtained with **11c**,**e**, due to competition between *N*- and *O*-mesylation. Some attempts were made to limit the reaction at nitrogen, by changing temperature (0 °C, 80 °C), activator (Tf_2_O), or base (NEt_3_), but all failed. The expected pyrrolidines **12c**,**e** were finally obtained by incorporating a transient Boc protecting group at nitrogen, enabling exclusive *O*-mesylation and subsequent cyclization after Boc deprotection with HCl and neutralization during work-up. Final deprotection of benzyl groups was performed with BCl_3_ in CH_2_Cl_2_, to afford bis-iminosugars **13a**–**e**, which were tested against glycosidases (see below).

**Scheme 3:**
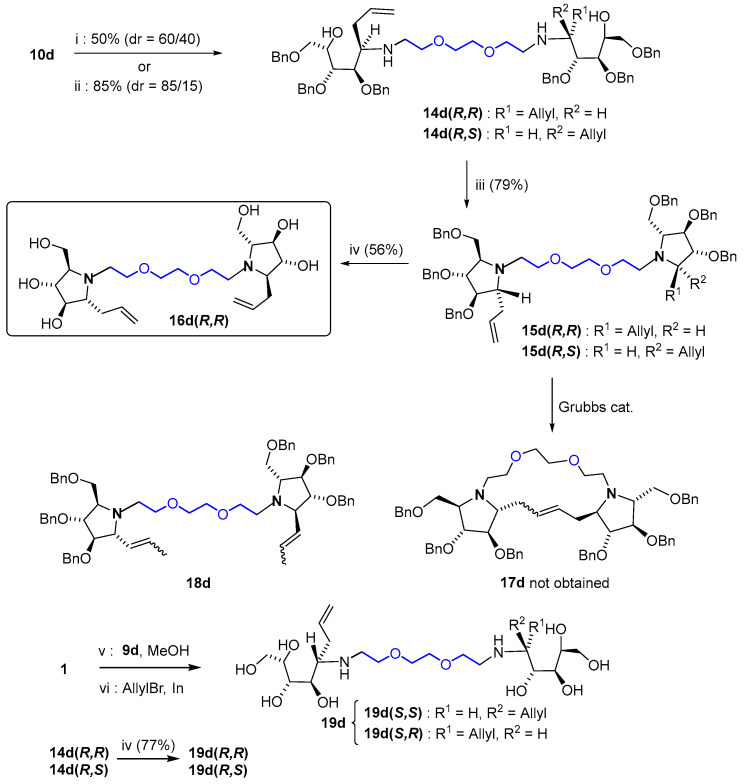
Synthesis of dimeric allyl-pyrrolidine **16d(*R*,*R*)** and determination of the configuration of **19d**. Reagents and conditions: (i) AllylMgCl, THF, 0 °C then r.t., 7 h; (ii) LiCl, AllylMgCl, THF, −78 °C then r.t., 2 h 30; (iii) MsCl, pyridine, THF, −78 °C then 0 °C, 3 h; (iv) BCl_3_, CH_2_Cl_2_, 0 °C, 2 h 30; (v) **9d** (0.5 equiv), MeOH, 45 °C, 4 h; (vi) AllylBr (6 equiv), indium powder (3 equiv), MeOH, r.t., 16 h, 58% (two steps).

The incorporation of an additional substituent onto the final pyrrolidine structure was attempted next (Scheme 3). To this aim, bis-glycosylamine **10d** was used as a model and was treated with an excess (6 equiv) of allyMgCl in THF, in the same manner as that described for monovalent glycosylamines [7]. A 60:40 mixture of two diastereoisomers **14d** was obtained (50% yield), which proved unseparable at this stage. According to their NMR spectra, the major isomer revealed a symmetrical structure (either (*R*,*R*) or (*S*,*S*) configurations at both new stereocenters, see below), whereas the minor one presented an unsymmetrical structure with two opposite configurations (*R*,*S*) generated during the addition. Yield and selectivity were improved in the presence of LiCl, an additive that proved efficient previously in nucleophilic additions to *N*-t-butanesulfinyl glycosylamines [49], affording an 85:15 mixture of diastereoisomers **14d(*R,R*)** and **14d(*R,S*)** in 85% yield.

A cyclization protocol (MsCl in pyridine) was applied to the mixture **14d(*R*,*R*)**/**14d(*R*,*S*)**, which gave pyrrolidines **15d(*R*,*R*)**/**15d(*R*,*S*)** in 79% yield. Both compounds were separated at this stage and the (*R*) configurations at the newly formed stereogenic centers in the structure of the major isomer were established using NOESY experiments. Deprotection was performed next on **15d(*R*,*R*)**, which encompasses an all-trans configuration, the one observed in most active glucosidase inhibitors such as the standard iminosugars DMDP or DNJ [20]. Dimeric allyl-pyrrolidine **16d(*R*,*R*)** obtained after treatment with BCl_3_ (56% yield) was also tested against our panel of commercial glycosidases. An attempt was conducted on **15d(*R*,*R*)** to promote metathesis in order to generate the macrocyclic structure **17d**. Unfortunately, cyclization proved unfavorable, and only isomerization of the double bonds was mediated by the Grubbs catalyst to give **18d**. Interestingly, a more direct procedure for allylation was conducted on unprotected d-xylose **1** by indium-mediated nucleophilic addition on an unprotected bis-glycosylamine, as previously described with monomeric glycosylamines [50]. The reaction proved successful and afforded polyhydroxy bis-allylamine **19d** in 58% yield. Chemical correlation was attempted to determine the configuration of the newly formed stereogenic centers in **19d**. To this end, debenzylation of a sample of the mixture **14d(*R*,*R*)**/**14d(*R*,*S*)** 85/15 with BCl_3_ afforded samples of **19d(*R*,*R*)**/**19d(*R*,*S*)** with ascertained configuration, which served as reference. According to their non-equivalent NMR spectra (see Supplementary Materials), **19d** and **19d(*R***,***R*)** feature distinct configurations, but both are symmetrical compounds in view of the simplicity of their NMR spectra. Thus, the (*S*,*S*) configuration was assigned to the major diastereoisomer resulting from indium-mediated allylation. The selectivity of this reaction is in full agreement with previous reactions on monomeric glycosylamines [50].

**Scheme 4:**
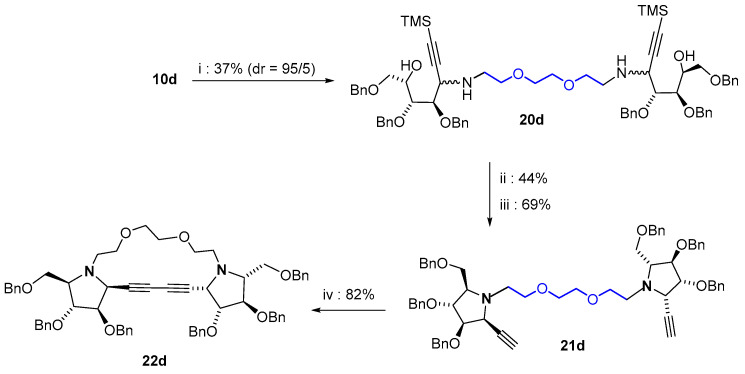
Synthesis of the glycophane **22d**. Reagents and conditions: (i) HC≡C-SiMe_3_, EtMgBr, THF, 0 °C then r.t., 17 h; (ii) MsCl, pyridine, THF, O °C then r.t., 6 h; (iii) TBAF, THF, r.t., 3 h; (iv) Cu(OAc)_2_·H_2_O, NiCl_2_, pyridine, EtOH, CHCl_3_, 60 °C, 16 h.

To go even further in the synthesis of macrocyclic structures by using our method, another strategy was designed, which would exploit alkyne/alkyne Glaser-type coupling to form the corresponding diyne (Scheme 4). To this aim, the addition of an ethynyl nucleophile was attempted on glycosylamine **10d**. Whereas acetylide (via the corresponding Grignard) afforded unsatisfactory results TMS-acetylide was added selectively to **10d** (d.e. = 90%), affording the expected bis-amine **20d** in 37% yield. Here, the (S) configuration was attributed to the newly formed stereocenters according to NOESY experiments conducted on the TMS-alkyne after cyclization, which is the opposite of that observed for the allyl-analogue. After the standard intramolecular nucleophilic substitution protocol mediated by MsCl, trimethylsilyl groups were removed (TBAF) to afford the bis-ethynylpyrrolidine **21d**. Gratifyingly, treatment of **21d** with copper(II) acetate monohydrate and nickel chloride to promote intramolecular diyne coupling afforded glycophane **22d** in 82% yield [51]. At this point, no tentative was made to debenzylate further **22d**.

Finally, enzymatic assays were performed with compounds **13a**–**e** and **16d** on a series of commercial glycosidases (α-glucosidase from rice and from yeast, β-glucosidase from almond, β-glucosidase and α-rhamnosidase from *A. niger*, β-galactosidase from *A. orizae*, α-mannosidase and β-*N*-acetylglucosaminidase from Jack bean and α-galactosidase from green coffee bean) to assess potency and selectivity of these new compounds (Table 1). A prepared sample of DAB-1 **8** was also tested under the same conditions for ease of comparison [52]. A first set of biological assays was effected at 1 mM concentration of inhibitor. Total annihilation (>95%) of enzyme activity at 1 mM is generally a prerequisite for high inhibition potencies. Conversely, % inhibition below 95% at 1 mM is typical of modest or poor inhibitors with half maximal inhibitory concentrations usually above 100 µM. Former results from our laboratories have always followed this empirical rule. Thus, IC_50_ was only determined in cases where inhibition at 1 mM was greater than 95%, by assaying decreasing concentrations of inhibitors.

DAB-1 **8** was chosen as a model iminosugar for our experiments examining the effect of dimerization. Pyrrolidine **8** entails a specific hydroxyl distribution closely related to that of glucose. As a consequence, DAB-1 has strong affinities for enzymes operating with glucosides or glucoconjugates, a result supported by our own experiments towards alpha- or beta-glucosidases from different origins with IC_50_’s in the range 0.69–79 µM (Table 1). However, structural analogies of DAB-1 with other biologically relevant carbohydrates such as mannose induce cross-inhibitions, as observed with α-mannosidase (IC_50_ = 49 µM). Regarding dimers **13a**–**e**, a significant decrease in glucosidase inhibition potencies is observed here, with only **13b** displaying potent alpha-glucosidase (baker’s yeast) inhibition with IC_50_ = 39 µM. However, a more detailed analysis of the results suggests that the linker separating the two pyrrolidine rings plays a role in the tightness of the inhibitor binding to the enzyme. Compound **13d** for instance, displays about 90% inhibition towards the two alpha-glucosidases and the beta-glucosidase from Aspergillus niger at 1 mM, whereas **13a** induces much weaker enzyme inhibition. The introduction of an additional substituent as found in **16d**, was detrimental for enzyme-inhibitor recognition regarding alpha-glucosidases. Whereas its non-substituted counterpart **13d** displayed 90% inhibition at 1 mM, only 26% inhibition was observed with allyl-pyrrolidine **16d** (rice alpha-glucosidase). An astonishing 20% enhancement of enzyme activity was observed in the presence of the latter regarding alpha-glucosidase from yeast. An analogous activation was observed with **16d** and **13d** towards beta-*N*-acetylglucosaminidase from Jack beans (33% and 38% activation, respectively). Concerning the other tested enzymes, dimerization caused a significant enhancement of alpha-mannosidase inhibition by **13c** and **13e** (IC_50_ = 18 µM and 34 µM, respectively) when compared to DAB-1 (IC_50_ = 49 µM) a trend already observed with other multimeric iminosugars towards this same enzyme [31]. Interestingly, whereas monomer **8** is a poor inhibitor of beta-galactosidase, dimeric structures **13a**–**13d** displayed >90% inhibition at 1 mM culminating to IC_50_’s = 2.8 µM and 31 µM in the case of **13b** and **13c**. Some β-galactosidase-inhibiting iminosugars have been considered for their pharmacological chaperone (PC) potency in the treatment of GM1 gangliosidosis, an inherited disorder caused by the disruption of human β-galactosidase. At low concentrations, such inhibitors impede the degradation of misfolded proteins, restoring residual activity in patients suffering from deficient enzymes. The potency and selectivity of bis-iminosugars **13** towards β-galactosidase, as observed here, make them valuable candidates for further studies on their use as PC in the treatment of GM1 gangliosidosis.

In conclusion, we describe here a new and straightforward synthetic route towards dimeric iminosugars, starting from bis-glycosylamines, which are easily prepared by the reaction of a carbohydrate with 0.5 equiv of a diamine. Reduction of such dimeric N-glycosides with LiAlH_4_ followed by MsCl-induced cyclization affords the protected bis-iminosugar, with the inverted configuration at C-4 when compared to the carbohydrate substrate. The method allows the integration of a variety of linkages (aryl, alkyl, polyethyleneglycol chains) connecting both iminosugar templates by the choice of the bis-amine used in the first step. Moreover, an additional substituent (allyl, ethynyl) may be inserted in the structure via nucleophilic addition of an organometallic reagent to the bis-glycosylamine in a highly diastereoselective manner. A bis-ethynyl symmetrical iminosugar proved susceptible to intramolecular Glaser coupling, affording an unprecedented macrocyclic structure. Finally, deprotected bis-iminosugars were tested towards a series of commercial glycosidases to uncover potencies and selectivities when compared to DAB-1, their monomeric counterpart. Whereas a significant drop in inhibition potencies was observed towards glucosidases when compared to DAB-1, compounds **13a**–**d** displayed unexpected potent inhibition of β-galactosidase. Further studies might be conducted in the future to evaluate the effect of bis-iminosugars **13a**–**d** on human β-galactosidase and explore their capacity to act as PC.

## 3. Materials and Methods

### 3.1. General Methods

Reactants and reagents were purchased from standard suppliers (Sigma-Aldrich (St. Louis, MO, USA), Alfa-Aesar (Haverhill, MA, USA), Fisher Scientific (Waltham, MA, USA)) and were used without further purification. Methanol, dichloromethane, tetrahydrofuran, and diethyl ether were dried on a Pure Solv MD system (Innovative Technology, Inc., Oldham, UK). Reactions were monitored on TLC plates using Silica gel F254 (0.2 mm) (Macherey-Nagel SAS, Hoerdt, France), detection was carried out by spraying with an aqueous solution of KMnO_4_ (2%)/Na_2_CO_3_ (4%) or alcoholic solutions of phosphomolybdic acid or p-anisaldehyde, followed by heating. Column chromatography purifications were performed over silica gel M 9385 (40–63 µm) Kieselgel 60 (Macherey-Nagel SAS, Hoerdt, France). NMR spectra were recorded on Bruker AC 500 (Billerica, MA, USA, 500 MHz for ^1^H, 126 MHz for ^13^C) or 600 (600 MHz for ^1^H and 150 MHz for ^13^C) spectrometers. Chemical shifts are expressed in parts per million (ppm) and were calibrated to deuterated or residual non-deuterated solvent peaks for ^1^H and ^13^C spectra. Coupling constants are in Hz and splitting pattern abbreviations are: br, broad; s, singlet; d, doublet; t, triplet; q, quadruplet; m, multiplet. DEPT and JMOD 1D NMR experiments, COSY, HSQC, HMBC, and NOESY 2D NMR experiments were used to confirm the NMR peak assignments for all compounds. Optical rotations were determined at 25 °C with an Anton Paar Model MCP 5100 polarimeter (Graz, Austria). Mass Spectra (MS) and High Resolution Mass Spectra (HRMS) were performed on a Waters Corp. Q-TOF Micro micromass positive ESI (CV = 30 V) (Milford, MA, USA).

### 3.2. General Procedure for the Synthesis of Bis-Glycosylamines ***10a***–***e***

To a solution of 2,3,5-tri-*O*-benzyl-l-xylofuranose **2** (1 equiv) in anhydrous dichloromethane was added molecular sieves (4 Å) then diamine **9a**–**e** (0.5 equiv), and the mixture was stirred at room temperature until total consumption of **2**. The solution was filtrated and concentrated under reduced pressure to afford an anomeric mixture of the bis-glycosylamine which was not purified further.

(**10a**) According to general procedure, the reaction of **2** (200 mg, 0.475 mmol) and **9a** (0.237 mmol, 32.3 mg) in dichloromethane (3.7 mL) in the presence of molecular sieves (450 mg) afforded after 48 h **10a** as a yellow oil. ^1^H NMR (500 MHz, CDCl_3_): δ 7.40–7.24 (m, 34 H, Ar-H), 5.03 (d, *J* = 3.5 Hz, 0.7 H, 2 × 1-H^min^), 4.76 (d, *J* = 2.2 Hz, 1.3 H, 2 × 1-H^maj^), 4.64–4.45 (m, 12 H, 6 × C*H*_2_-Ph^maj^ + 6 × C*H*_2_-Ph^min^), 4.41 (q, *J* = 6.0 Hz, 0.7 H, 2 × 4-H^min^), 4.34 (q, *J* = 5.3 Hz, 1.3 H, 2 × 4-H^maj^), 4.15 (d, *J* = 13.5 Hz, 0.7 H, 2 × C*H_a_*H_b_-NH^min^), 4.07 (dd, *J* = 13.4, 3.6 Hz, 1.3 H, 2 × C*H_a_*H_b_-NH^maj^), 4.01–3.98 (m, 2 H, 2 × 3-H^maj^ + 2 × 3-H^min^), 3.95–3.93 (m, 1.3 H, 2 × 2-H^maj^), 3.91–3.89 (m, 0.7 H, 2 × 2-H^min^), 3.82–3.68 (m, 6 H, 2 × CH_a_*H_b_*-NH^maj^ + 2 × CH_a_*H_b_*-NH^min^ + 2 × 5-H^maj^ + 2 × 5-H^min^) ppm; ^13^C NMR (125 MHz, CDCl_3_): δ 140.7 (C^IV^), 140.4 (C^IV^), 138.5 (C^IV^), 138.4 (C^IV^), 138.1 (C^IV^), 138.0 (C^IV^), 137.9 (C^IV^), 128.7, 128.6, 128.5, 128.5, 128.2, 128.2, 128.1, 128.0, 127.9, 127.9, 127.8, 127.7, 127.7, 127.1, 126.8, 126.7 (C-Ar), 94.6 (C-1^maj^), 90.5 (C-1^min^), 86.1 (C-2^maj^), 81.6 (C-3^maj^), 81.4 (C-3^min^), 81.4 (C-2^min^), 78.9 (C-4^maj^), 77.2 (C-4^min^), 73.6 (*C*H_2_-Ph), 72.9 (*C*H_2_-Ph^min^), 72.4 (*C*H_2_-Ph^min^), 72.2 (*C*H_2_-Ph^maj^), 71.7 (*C*H_2_-Ph^maj^), 69.3 (C-5^maj^), 69.0 (C-5^min^), 50.2 (*C*H_2_-NH^min^), 49.9 (*C*H_2_-NH^maj^) ppm; HRMS (ESI) *m*/*z* calcd for [C_60_H_64_N_2_O_8_ + H]^+^: 941.4741, found 941.4745.

(**10b**) According to general procedure, the reaction of **2** (436 mg, 1.04 mmol) and **9b** (71 mg, 0.52 mmol) in dichloromethane (7.8 mL) in the presence of molecular sieves (950 mg) afforded after 27 h **10b** as a yellow oil. ^1^H NMR (500 MHz, CDCl_3_): δ 7.40–7.27 (m, 34 H, Ar-H), 5.00–4.98 (m, 0.7 H, 2 × 1-H^min^), 4.73–4.72 (m, 1.3 H, 2 × 1-H^maj^), 4.68–4.47 (m, 12 H, 6 × C*H*_2_-Ph^maj^ + 6 × C*H*_2_-Ph^min^), 4.39 (q, *J* = 5.7 Hz, 0.7 H, 2 × 4-H^min^), 4.36 (q, *J* = 5.2 Hz, 1.3 H, 2 × 4-H^maj^), 4.16 (d, *J* = 13.5 Hz, 0.7 H, 2 × C*H_a_*H_b_-NH^min^), 4.08 (d, *J* = 13.2 Hz, 1.3 H, 2 × C*H_a_*H_b_-NH^maj^), 4.03–4.00 (m, 2 H, 2 × 3-H^maj^ + 2 × 3-H^min^), 3.96–3.95 (m, 1.3 H, 2 × 2-H^maj^), 3.92–3.71 (m, 6.7 H, 2 × 2-H^min^ + 2 × CH_a_*H_b_*-NH^maj^ + 2 × CH_a_*H_b_*-NH^min^ + 2 × 5-H^maj^ + 2 × 5-H^min^) ppm; ^13^C NMR (125 MHz, CDCl_3_): δ 139.1 (C^IV^), 138.9 (C^IV^), 138.5 (C^IV^), 138.3 (C^IV^), 138.0 (C^IV^), 138.0 (C^IV^), 137.9 (C^IV^), 128.7, 128.7, 128.6, 128.5, 128.5, 128.5, 128.4, 128.3, 128.2, 128.2, 128.2, 128.1, 128.1, 128.0, 127.9, 127.9, 127.8, 127.7, 127.7 (C-Ar), 94.4 (C-1^maj^), 90.4 (C-1^min^), 86.0 (C-2^maj^), 81.6 (C-3^maj^), 81.4 (C-3^min^), 81.3 (C-2^min^), 78.9 (C-4^maj^), 77.2 (C-4^min^), 73.6 (*C*H_2_-Ph), 72.8 (*C*H_2_-Ph^min^), 72.3 (*C*H_2_-Ph^min^), 72.2 (*C*H_2_-Ph^maj^), 71.7 (*C*H_2_-Ph^maj^), 69.2 (C-5^maj^), 69.0 (C-5^min^), 49.9 (*C*H_2_-NH^min^), 49.6 (*C*H_2_-NH^maj^) ppm; HRMS (ESI) *m*/*z* calcd for [C_60_H_64_N_2_O_8_ + H]^+^: 941.4741, found 941.4742.

(**10c**) According to general procedure, the reaction of **2** (600 mg, 1.43 mmol) and **9c** (103 mg, 0.71 mmol) in dichloromethane (11.1 mL) in the presence of molecular sieves (1.35 g) afforded after 23 h **10c** as a yellow oil. ^1^H NMR (500 MHz, CDCl_3_): δ 7.40–7.26 (m, 30 H, Ar-H), 5.04 (d, *J* = 3.4 Hz, 0.7 H, 2 × 1-H^min^), 4.77 (d, *J* = 2.2 Hz, 1.3 H, 2 × 1-H^maj^), 4.66–4.47 (m, 12 H, 6 × C*H*_2_-Ph^maj^ + 6 × C*H*_2_-Ph^min^), 4.39 (q, *J* = 5.5 Hz, 0.7 H, 2 × 4-H^min^), 4.34 (q, *J* = 5.4 Hz, 1.3 H, 2 × 4-H^maj^), 4.01–3.99 (m, 2 H, 2 × 3-H^maj^ + 2 × 3-H^min^), 3.92–3.89 (m, 2 H, 2 × 2-H^maj^ + 2 × 2-H^min^), 3.80–3.67 (m, 4 H, 2 × 5-H^maj^ + 2 × 5-H^min^), 3.01–2.87 (m, 2 H, 2 × C*H_a_*H_b_-NH^maj^ + 2 × C*H_a_*H_b_-NH^min^), 2.69–2.57 (m, 2 H, 2 × CH_a_*H_b_*-NH^maj^ + 2 × CH_a_*H_b_*-NH^min^), 1.53–1.28 (m, 12 H, 6 × CH_2_^maj^ + 6 × CH_2_^min^) ppm; ^13^C NMR (125 MHz, CDCl_3_): δ 138.5 (C^IV^), 138.4 (C^IV^), 138.1 (C^IV^), 138.0 (C^IV^), 137.9 (C^IV^), 137.9 (C^IV^), 128.7, 128.6, 128.5, 128.5, 128.5, 128.5, 128.4, 128.4, 128.1, 128.1, 128.1, 128.0, 128.0, 128.0, 127.9, 127.9, 127.8, 127.8, 127.7, 127.7 (C-Ar), 95.1 (C-1^maj^), 91.0 (C-1^min^), 86.1 (C-2^maj^), 81.6 (C-3^maj^), 81.5 (C-3^min^), 81.2 (C-2^min^), 78.8 (C-4^maj^), 77.2 (C-4^min^), 73.5 (*C*H_2_-Ph), 72.8 (*C*H_2_-Ph^min^), 72.4 (*C*H_2_-Ph^min^), 72.2 (*C*H_2_-Ph^maj^), 71.7 (*C*H_2_-Ph^maj^), 69.2 (C-5^maj^), 68.9 (C-5^min^), 46.6 (*C*H_2_-NH^min^), 46.1 (*C*H_2_-NH^maj^), 30.8 (CH_2_), 30.5 (CH_2_), 29.6 (CH_2_), 29.6 (CH_2_), 27.4 (CH_2_), 27.4 (CH_2_), 27.4 (CH_2_), 27.3 (CH_2_) ppm; HRMS (ESI) *m*/*z* calcd for [C_60_H_72_N_2_O_8_ + H]^+^: 949.5372, found 949.5367.

(**10d**) According to general procedure, the reaction of **2** (100 mg, 0.24 mmol) and **9d** (18 mg, 0.12 mmol) in dichloromethane (2 mL) in the presence of molecular sieves (227 mg) afforded after 24 h **10d** as a brown oil. ^1^H NMR (500 MHz, CDCl_3_): δ 7.39–7.25 (m, 30 H, Ar-H), 4.97 (d, *J* = 3.6 Hz, 0.6 H, 2 × 1-H^min^), 4.72–4.71 (m, 1.4 H, 2 × 1-H^maj^), 4.65–4.45 (m, 12 H, 6 × C*H*_2_-Ph^maj^ + 6 × C*H*_2_-Ph^min^), 4.37 (q, *J* = 5.9 Hz, 0.6 H, 2 × 4-H^min^), 4.34 (q, *J* = 5.4 Hz, 1.4 H, 2 × 4-H^maj^), 3.99–3.98 (m, 2 H, 2 × 3-H^maj^ + 2 × 3-H^min^), 3.93 (m, 1.4 H, 2 × 2-H^maj^), 3.90–3.89 (m, 0.6 H, 2 × 2-H^min^), 3.80–3.68 (m, 4 H, 2 × 5-H^maj^ + 2 × 5-H^min^), 3.62–3.52 (m, 8 H, 4 × CH_2_^maj^ + 4 × CH_2_^min^), 3.19–3.07 (m, 2 H, 2 × C*H_a_*H_b_-NH^maj^ + 2 × C*H_a_*H_b_-NH^min^), 2.88–2.75 (m, 2 H, 2 × CH_a_*H_b_*-NH^maj^ + 2 × CH_a_*H_b_*-NH^min^) ppm; ^13^C NMR (125 MHz, CDCl_3_): δ 138.5 (C^IV^), 138.4 (C^IV^), 138.1 (C^IV^), 138.0 (C^IV^), 137.9 (C^IV^), 137.9 (C^IV^), 128.7, 128.7, 128.5, 128.5, 128.4, 128.4, 128.0, 127.9, 127.9, 127.8, 127.7 (C-Ar), 95.3 (C-1^maj^), 90.9 (C-1^min^), 86.1 (C-2^maj^), 81.6 (C-3^min^), 81.5 (C-3^maj^), 81.3 (C-2^min^), 78.9 (C-4^maj^), 77.2 (C-4^min^), 73.5 (*C*H_2_-Ph^maj^), 73.5 (*C*H_2_-Ph^min^), 72.8 (*C*H_2_-Ph^min^), 72.4 (*C*H_2_-Ph^min^), 72.2 (*C*H_2_-Ph^maj^), 71.7 (*C*H_2_-Ph^maj^), 71.5 (CH_2_^min^), 71.3 (CH_2_^maj^), 70.4 (CH_2_^min^), 70.3 (CH_2_^min^), 70.2 (CH_2_^maj^), 70.2 (CH_2_^maj^), 69.2 (C-5^maj^), 68.9 (C-5^min^), 46.1 (*C*H_2_-NH^min^), 45.7 (*C*H_2_-NH^maj^) ppm; HRMS (ESI) *m*/*z* calcd for [C_58_H_68_N_2_O_10_ + H]^+^: 953.4952, found 953.4957.

(**10e**) According to general procedure, the reaction of **2** (157 mg, 0.37 mmol) and **9e** (41 mg, 0.18 mmol) in dichloromethane (3.1 mL) in the presence of molecular sieves (356 mg) afforded after 27 h **10e** as a yellow oil. ^1^H NMR (500 MHz, CDCl_3_): δ 7.39–7.26 (m, 30 H, Ar-H), 4.95 (d, *J* = 3.6 Hz, 0.7 H, 2 × 1-H^min^), 4.68 (d, *J* = 2.2 Hz, 1.3 H, 2 × 1-H^maj^), 4.65–4.45 (m, 12 H, 6 × C*H*_2_-Ph^maj^ + 6 × C*H*_2_-Ph^min^), 4.36 (q, *J* = 6.0 Hz, 0.7 H, 2 × 4-H^min^), 4.30 (q, *J* = 5.4 Hz, 1.3 H, 2 × 4-H^maj^), 3.99–3.97 (m, 2 H, 2 × 3-H^maj^ + 2 × 3-H^min^), 3.87 (m, 2 H, 2 × 2-H^maj^ + 2 × 2-H^min^), 3.76–3.65 (m, 4 H, 2 × 5-H^maj^ + 2 × 5-H^min^ H^min^), 3.62–3.48 (m, 12 H, 6 × CH_2_^maj^ + 6 × CH_2_^min^), 3.06–2.93 (m, 2 H, 2 × C*H_a_*H_b_-NH^maj^ + 2 × C*H_a_*H_b_-NH^min^), 2.78–2.66 (m, 2 H, 2 × CH_a_*H_b_*-NH^maj^ + 2 × CH_a_*H_b_*-NH^min^), 1.81–1.69 (m, 4 H, 2 × C*H_2_*-CH_2_-NH^maj^ + 2 × C*H_2_*-CH_2_-NH^min^) ppm; ^13^C NMR (125 MHz, CDCl_3_): δ 138.5 (C^IV^), 138.4 (C^IV^), 138.1 (C^IV^), 138.0 (C^IV^), 137.9 (C^IV^), 128.7, 128.6, 128.5, 128.5, 128.5, 128.0, 127.9, 127.9, 127.9, 127.8, 127.8, 127.7, 127.7 (C-Ar), 95.2 (C-1^maj^), 91.0 (C-1^min^), 86.2 (C-2^maj^), 81.6 (C-3^maj^), 81.4 (C-2^min^), 81.2 (C-3^min^), 78.7 (C-4^maj^), 77.2 (C-4^min^), 73.6 (*C*H_2_-Ph^j^), 72.8 (*C*H_2_-Ph^min^), 72.4 (*C*H_2_-Ph^min^), 72.2 (*C*H_2_-Ph^maj^), 71.7 (*C*H_2_-Ph^maj^), 70.7 (CH_2_), 70.3 (CH_2_), 69.8 (CH_2_), 69.7 (CH_2_), 69.6 (CH_2_), 69.2 (C-5^maj^), 68.9 (C-5^min^), 43.7 (*C*H_2_-NH^min^), 43.3 (*C*H_2_-NH^maj^), 30.7 (*C*H_2_-CH_2_-NH^min^), 30.6 (*C*H_2_-CH_2_-NH^maj^) ppm; HRMS (ESI) *m*/*z* calcd for [C_62_H_76_N_2_O_11_ + H]^+^: 1025.5527, found 1025.5531.

### 3.3. Representative Procedure for the Reduction of Bis-Glycosylamines ***10a***–***e***

To a solution of bis-glycosylamine **10a**–**e** (1 equiv) in anhydrous diethyl ether at 0 °C under argon atmosphere was added dropwise lithium aluminium hydride 1 M in diethyl ether (4 equiv). The mixture was stirred for 1 h at the same temperature and then sequentially supplemented dropwise with water (38 µL per mL of LiAlH_4_ 1 M used), an aqueous solution of sodium hydroxide 3 M (38 µL per mL of LiAlH_4_ 1 M used) and water again (114 µL per mL of LiAlH_4_ 1 M used). The resulting mixture was filtered, the inorganic residue was washed with ether, and the resulting ether solution was then combined with the organic layer of the filtrate. The solution was dried with magnesium sulfate, filtered, and concentrated under reduced pressure, and finally, the crude was purified by column chromatography on silica gel.

(**11a**) According to general procedure, the reaction of **10a** (232 mg, 0.246 mmol) and LiAlH_4_ 1 M in diethyl ether (984 µL, 0.984 mmol) in diethyl ether (2.5 mL) afforded after purification (eluent: EtOAc/PE gradient, 80/20 to 95/5) **11a** (149 mg, 0.158 mmol, 64%) as a yellow oil. ^1^H NMR (500 MHz, CDCl_3_): δ 7.36–7.15 (m, 34 H, Ar-H), 4.56–4.42 (m, 12 H, 6 × C*H_2_*-Ph), 4.09–4.06 (m, 2 H, 2 × 4-H), 3.83–3.76 (m, 4 H, 2 × 3-H + 2 × NH-C*H_a_*H_b_-Ph), 3.72–3.52 (m, 8 H, 2 × 2-H + 2 × NH-CH_a_*H_b_*-Ph + 2 × 5-H), 2.97 (dd, *J* = 12.2, 1.4 Hz, 2 H, 2 × 1-*H_a_*H_b_), 2.83 (dd, *J* = 12.2, 5.1 Hz, 2 H, 2 × 1-H_a_*H_b_*) ppm; ^13^C NMR (125 MHz, CDCl_3_): δ 138.5 (C^IV^), 138.4 (C^IV^), 138.1 (C^IV^), 129.2, 128.6, 128.6, 128.5, 128.5, 128.4, 128.4, 128.0, 128.0, 127.9, 127.9, 127.9, 127.8, 127.7, 127.7, 127.7, 127.7 (C-Ar), 76.8 (C-3), 76.2 (C-2), 73.8 (*C*H_2_-Ph), 73.3 (*C*H_2_-Ph), 72.1 (*C*H_2_-Ph), 70.9 (C-5), 66.7 (C-4), 53.6 (NH-*C*H_2_-Ph), 46.5 (C-1) ppm; ${\left[\alpha \right]}_{D}^{25}$ = +6.3 (*c* 1, CHCl_3_). HRMS (ESI) *m*/*z* calcd for [C_60_H_68_N_2_O_8_ + H]^+^: 945.5054, found 945.5063.

(**11b**) According to general procedure, the reaction of **10b** (583 mg, 0.619 mmol) and LiAlH_4_ 1 M in diethyl ether (2.48 mL, 2.476 mmol) in diethyl ether (6 mL) afforded after purification (eluent: EtOAc/PE gradient, 80/20 to 95/5) **11b** (357 mg, 0.377 mmol, 61%) as a yellow oil. ^1^H NMR (500 MHz, CDCl_3_): δ 7.36–7.22 (m, 34 H, Ar-H), 4.56–4.41 (m, 12 H, 6 × C*H_2_*-Ph), 4.09–4.06 (m, 2 H, 2 × 4-H), 3.81–3.77 (m, 4 H, 2 × 3-H + 2 × NH-C*H_a_*H_b_-Ph), 3.68–3.61 (m, 6 H, 2 × 2-H + 2 × NH-CH_a_*H_b_*-Ph + 2 × 5-*H_a_*H_b_), 3.58–3.54 (m, 2 H, 2 × 5-H_a_*H_b_*), 2.95 (dd, *J* = 12.2, 1.4 Hz, 2 H, 2 × 1-*H_a_*H_b_), 2.83 (dd, *J* = 12.2, 5.0 Hz, 2 H, 2 × 1-H_a_*H_b_*) ppm; ^13^C NMR (125 MHz, CDCl_3_): δ 138.6 (C^IV^), 138.4 (C^IV^), 138.1 (C^IV^), 137.9 (C^IV^), 128.8, 128.7, 128.7, 128.6, 128.6, 128.5, 128.4, 128.4, 128.0, 127.9, 127.9, 127.6 (C-Ar), 76.7 (C-3), 76.2 (C-2), 73.8 (*C*H_2_-Ph), 73.3 (*C*H_2_-Ph), 72.1 (*C*H_2_-Ph), 70.9 (C-5), 66.5 (C-4), 53.5 (NH-*C*H_2_-Ph), 46.3 (C-1) ppm; ${\left[\alpha \right]}_{D}^{25}$ = +14.8 (*c* 1, CHCl_3_). HRMS (ESI) *m*/*z* calcd for [C_60_H_68_N_2_O_8_ + H]^+^: 945.5054, found 945.5051.

(**11c**) According to general procedure, the reaction of **10c** (597 mg, 0.629 mmol) and LiAlH_4_ 1 M in diethyl ether (2.52 mL, 2.516 mmol) in diethyl ether (6 mL) afforded after purification (eluent: EtOAc/PE 70/30 then EtOAc/MeOH 95/5) **11c** (388 mg, 0.407 mmol, 65%) as a yellow oil. ^1^H NMR (500 MHz, CDCl_3_): δ 7.37–7.25 (m, 30 H, Ar-H), 4.57–4.47 (m, 12 H, 6 × C*H*_2_-Ph), 4.09–4.06 (m, 2 H, 2 × 4-H), 3.76 (d, *J* = 5.8 Hz, 2 H, 2 × 3-H), 3.67–3.61 (m, 4 H, 2 × 2-H + 2 × 5-*H_a_*H_b_), 3.56 (t, *J* = 8.5 Hz, 2 H, 2 × 5-H_a_*H_b_*), 2.90 (dd, *J* = 12.2, 1.3 Hz, 2 H, 2 × 1-*H_a_*H_b_), 2.79 (dd, *J* = 12.2, 5.0 Hz, 2 H, 2 × 1-H_a_*H_b_*), 2.55 (t, *J* = 7.4 Hz, 4 H, 2 × NH-C*H_2_*-CH_2_), 1.53–1.38 (m, 4 H, 2 × NH-CH_2_-C*H_2_*), 1.28–1.24 (m, 8 H, 4 × CH_2_) ppm; ^13^C NMR (125 MHz, CDCl_3_): δ 138.6 (C^IV^), 138.5 (C^IV^), 138.2 (C^IV^), 128.6, 128.6, 128.5, 128.5, 128.4, 128.4, 128.0, 127.9, 127.9, 127.9, 127.6 (C-Ar), 76.7 (C-3), 76.2 (C-2), 73.7 (*C*H_2_-Ph), 73.3 (*C*H_2_-Ph), 72.0 (*C*H_2_-Ph), 71.0 (C-5), 66.3 (C-4), 49.6 (NH-*C*H_2_-CH_2_), 46.5 (C-1), 29.5 (NH-CH_2_-*C*H_2_), 29.4 (CH_2_), 27.3 (CH_2_) ppm; ${\left[\alpha \right]}_{D}^{25}$ = +20.3 (*c* 1, CHCl_3_). HRMS (ESI) *m*/*z* calcd for [C_60_H_76_N_2_O_8_ + H]^+^: 953.5680, found 953.5685.

(**11d**) According to general procedure, the reaction of **10d** (1.07 g, 1.12 mmol) and LiAlH_4_ 1 M in diethyl ether (4.48 mL, 4.48 mmol) in diethyl ether (11 mL) afforded after purification (eluent: EtOAc/MeOH gradient, 80/20 to 50/50) **11d** (773 mg, 0.806 mmol, 72%) as a yellow oil. ^1^H NMR (500 MHz, CDCl_3_): δ 7.37–7.25 (m, 30 H, Ar-H), 4.57–4.45 (m, 12 H, 6 × C*H_2_*-Ph), 4.10 (t, *J* = 6.7 Hz, 2 H, 2 × 4-H), 3.76 (dm, *J* = 5.9 Hz, 2 H, 2 × 3-H), 3.68–3.66 (m, 2 H, 2 × 2-H), 3.64–3.49 (m, 12 H, 2 × 5-H + 4 × CH_2_), 2.90 (dd, *J* = 12.5, 1.6 Hz, 2 H, 2 × 1-*H_a_*H_b_), 2.83 (dd, *J* = 12.5, 4.8 Hz, 2 H, 2 × 1-H_a_*H_b_*), 2.80–2.70 (m, 4 H, 2 × NH-C*H_2_*-CH_2_) ppm; ^13^C NMR (125 MHz, CDCl_3_): δ 138.5 (C^IV^), 138.4 (C^IV^), 138.1 (C^IV^), 128.5, 128.4, 128.3, 128.3, 127.8, 127.8, 127.8, 127.5 (C-Ar), 76.9 (C-3), 76.2 (C-2), 73.6 (*C*H_2_-Ph), 73.2 (*C*H_2_-Ph), 71.8 (*C*H_2_-Ph), 71.0 (C-5), 70.3 (CH_2_), 69.7 (CH_2_), 66.4 (C-4), 48.7 (NH-*C*H_2_-CH_2_), 46.2 (C-1) ppm; ${\left[\alpha \right]}_{D}^{25}$ = +14.9 (*c* 1, CHCl_3_). HRMS (ESI) *m*/*z* calcd for [C_58_H_72_N_2_O_10_ + H]^+^: 957.5265, found 957.5270.

(**11e**) According to general procedure, the reaction of **10e** (0.578 g, 0.564 mmol) and LiAlH_4_ 1 M in diethyl ether (2.26 mL, 2.26 mmol) in diethyl ether (5 mL) afforded after purification (eluent: EtOAc/MeOH gradient, 80/20 to 50/50) **11e** (336 mg, 0.327 mmol, 58%) as a yellow oil. ^1^H NMR (500 MHz, CDCl_3_): δ 7.37–7.25 (m, 30 H, Ar-H), 4.56–4.45 (m, 12 H, 6 × C*H_2_*-Ph), 4.10–4.07 (m, 2 H, 2 × 4-H), 3.77 (dm, *J* = 5.8 Hz, 2 H, 2 × 3-H), 3.69–3.67 (m, 2 H, 2 × 2-H), 3.65–3.45 (m, 16 H, 2 × 5-H + 6 × CH_2_), 2.91 (dd, *J* = 12.2, 1.1 Hz, 2 H, 2 × 1-*H_a_*H_b_), 2.82 (dd, *J* = 12.2, 4.9 Hz, 2 H, 2 × 1-H_a_*H_b_*), 2.75–2.64 (m, 4 H, 2 × NH-C*H_2_*-CH_2_), 1.79–1.74 (m, 4 H, 2 × NH-CH_2_-C*H_2_*) ppm; ^13^C NMR (125 MHz, CDCl_3_): δ 138.5 (C^IV^), 138.3 (C^IV^), 138.1 (C^IV^), 128.4, 128.4, 128.3, 128.3, 128.3, 128.0, 127.8, 127.8, 127.7, 127.7, 127.5 (C-Ar), 76.6 (C-3), 76.0 (C-2), 73.5 (*C*H_2_-Ph), 73.1 (*C*H_2_-Ph), 71.8 (*C*H_2_-Ph), 70.9 (C-5), 70.5 (CH_2_), 70.1 (CH_2_), 69.5 (CH_2_), 66.3 (C-4), 46.9 (NH-*C*H_2_-CH_2_), 46.4 (C-1), 29.2 (NH-CH_2_-*C*H_2_) ppm; ${\left[\alpha \right]}_{D}^{25}$ = +8.0 (*c* 1, CHCl_3_). HRMS (ESI) *m*/*z* calcd for [C_62_H_80_N_2_O_11_ + H]^+^: 1029.5840, found 1029.5833.

### 3.4. Representative Procedure for the Synthesis of Dimeric Iminosugars ***12a***,***b***,***d***

To a solution of **11a**,**b**,**d** (1 equiv) in pyridine at 0 °C was added dropwise methanesulfonyl chloride (2.4 equiv). The mixture was stirred for 2.5 h at the same temperature then the reaction was quenched with water (10 mL) and extracted with ethyl acetate (3 × 5 mL). The organic phase was dried with magnesium sulfate, filtered, and concentrated under reduced pressure, and finally, the crude was purified by column chromatography on silica gel.

(**12a**) According to general procedure, the reaction of **11a** (187 mg, 0.198 mmol) and methanesulfonyl chloride (38 µL, 0.475 mmol) in pyridine (2.5 mL) afforded after purification (eluent: EtOAc/PE gradient, 20/80 to 30/70) **12a** (33 mg, 0.036 mmol, 19%) as a yellow oil. ^1^H NMR (500 MHz, CDCl_3_): δ 7.33–7.27 (m, 34 H, Ar-H), 4.54–4.53 (m, 8 H, 4 × C*H_2_*-Ph), 4.46 (d, *J* = 12.2 Hz, 2 H, 2 × C*H_a_*H_b_Ph), 4.38 (d, *J* = 12.2 Hz, 2 H, 2 × CH_a_*H_b_*Ph), 4.14 (d, *J* = 12.2 Hz, 2 H, 2 × N-C*H_a_*H_b_Ar), 3.93–3.91 (m, 4 H, 2 × 3-H + 2 × 4-H), 3.64–3.62 (m, 4 H, 2 × 6-H), 3.49 (d, *J* = 12.2 Hz, 2 H, 2 × N-CH_a_*H_b_*Ar), 3.05 (d, *J* = 10.7 Hz, 2 H, 2 × 2-*H_a_*H_b_), 2.89 (q, *J* = 5.2 Hz, 2 H, 2 × 5-H), 2.58 (dd, *J* = 10.7, 5.1 Hz, 2 H, 2 × 2-H_a_*H_b_*) ppm; ^13^C NMR (125 MHz, CDCl_3_): δ 138.7 (C^IV^), 138.6 (C^IV^), 138.4 (C^IV^), 129.7, 128.5, 128.4, 128.2, 127.9, 127.9, 127.8, 127.7, 127.7, 127.6 (C-Ar), 86.1 (C-4), 81.7 (C-3), 73.3 (*C*H_2_-Ph), 71.5 (*C*H_2_-Ph), 71.4 (C-6), 71.0 (*C*H_2_-Ph), 68.6 (C-5), 59.2 (N-*C*H_2_-Ar), 57.1 (C-2) ppm; ${\left[\alpha \right]}_{D}^{25}$ = +22.2 (*c* 1, CHCl_3_). HRMS (ESI) *m*/*z* calcd for [C_60_H_64_N_2_O_6_ + H]^+^: 909.4843, found 909.4849.

(**12b**) According to general procedure, the reaction of **11b** (166 mg, 0.176 mmol) and methanesulfonyl chloride (33 µL, 0.422 mmol) in pyridine (2 mL) afforded after purification (eluent: EtOAc/PE gradient, 20/80 to 30/70) **12b** (65 mg, 0.071 mmol, 41%) as a yellow oil. ^1^H NMR (500 MHz, CDCl_3_): δ 7.34–7.27 (m, 34 H, Ar-H), 4.54–4.53 (m, 8 H, 4 × C*H_2_*-Ph), 4.47 (d, *J* = 12.2 Hz, 2 H, 2 × C*H_a_*H_b_Ph), 4.39 (d, *J* = 12.2 Hz, 2 H, 2 × CH_a_*H_b_*Ph), 4.13 (d, *J* = 13.2 Hz, 2 H, 2 × N-C*H_a_*H_b_Ar), 3.94–3.92 (m, 4 H, 2 × 3-H + 2 × 4-H), 3.65–3.61 (m, 4 H, 2 × 6-H), 3.49 (d, *J* = 13.2 Hz, 2 H, 2 × N-CH_a_*H_b_*Ar), 3.06 (d, *J* = 10.7 Hz, 2 H, 2 × 2-*H_a_*H_b_), 2.88 (q, *J* = 5.4 Hz, 2 H, 2 × 5-H), 2.58 (dd, *J* = 10.7, 5.2 Hz, 2 H, 2 × 2-H_a_*H_b_*) ppm; ^13^C NMR (125 MHz, CDCl_3_): δ 138.6 (C^IV^), 138.4 (C^IV^), 137.5 (C^IV^), 129.0, 128.5, 127.9, 127.8, 127.7, 127.7, 127.6 (C-Ar), 86.0 (C-4), 81.7 (C-3), 73.4 (*C*H_2_-Ph), 71.6 (*C*H_2_-Ph), 71.4 (C-6), 71.0 (*C*H_2_-Ph), 68.6 (C-5), 59.0 (N-*C*H_2_-Ar), 57.1 (C-2) ppm; ${\left[\alpha \right]}_{D}^{25}$ = +18.0 (*c* 1, CHCl_3_).

(**12d**) According to general procedure, the reaction of **11d** (197 mg, 0.206 mmol) and methanesulfonyl chloride (39 µL, 0.494 mmol) in pyridine (3 mL) afforded after purification (eluent: EtOAc/PE 70/30) **12d** (66 mg, 0.072 mmol, 35%) as a yellow oil. ^1^H NMR (500 MHz, CDCl_3_): δ 7.37–7.28 (m, 30 H, Ar-H), 4.57–4.50 (m, 10 H, 4 × C*H_2_*-Ph + 2 × C*H_a_*H_b_-Ph), 4.46 (d, *J* = 12.3 Hz, 2 H, 2 × CH_a_*H_b_*Ph), 3.94 (d, *J* = 5 Hz, 2 H, 2 × 3-H), 3.89 (d, *J* = 3.9 Hz, 2 H, 2 × 4-H), 3.64–3.53 (m, 12 H, 2 × 6-H + 4 × CH_2_), 3.27 (d, *J* = 10.6 Hz, 2 H, 2 × 2-*H_a_*H_b_), 3.11 (dt, *J* = 12.7, 6.3 Hz, 2 H, 2 × N-C*H_a_*H_b_-CH_2_), 2.82 (q, *J* = 5.0 Hz, 2 H, 2 × 5-H), 2.71 (dd, *J* = 10.6, 5.1 Hz, 2 H, 2 × 2-H_a_*H_b_*), 2.65 (dt, *J* = 12.7, 6.3 Hz, 2 H, 2 × N-CH_a_*H_b_*-CH_2_) ppm; ^13^C NMR (125 MHz, CDCl_3_): δ 138.5 (C^IV^), 138.4 (C^IV^), 138.3 (C^IV^), 128.4, 127.9, 127.9, 127.9, 127.8, 127.7, 127.7, 127.7, 127.6 (C-Ar), 85.3 (C-4), 81.8 (C-3), 73.3 (*C*H_2_-Ph), 71.4 (*C*H_2_-Ph), 71.1 (*C*H_2_-Ph), 71.0 (C-6), 70.4 (CH_2_), 70.3 (CH_2_), 69.5 (C-5), 58.2 (C-2), 54.5 (N-*C*H_2_-CH_2_) ppm; ${\left[\alpha \right]}_{D}^{25}$ = −14.1 (*c* 1, CHCl_3_). HRMS (ESI) *m*/*z* calcd for [C_58_H_69_N_2_O_8_ + H]^+^: 921.5054, found 921.5046.

### 3.5. Representative Procedure for the Synthesis of Dimeric Iminosugars ***12c***,***e***

Step 1, protection of amine functions: To a solution of **11c**,**e** (1 equiv) in anhydrous methanol (10 mL) at room temperature were successively added triethylamine (4 equiv), 4-(dimethylamino)pyridine (0.2 equiv) and di-tert-butyl dicarbonate (6 equiv). The resulting mixture was stirred until the total consumption of the starting material (typically 12 h for **11c** and 1h for **11e**) then the reaction was quenched with water (10 mL) and extracted with dichloromethane (3 × 5 mL). The organic phase was dried with magnesium sulfate, filtered, and concentrated under reduced pressure. The resulting product was used in the next step without further purification.

Step 2, *O*-mesylation: To the crude obtained in step 1 was added dropwise at 0 °C triethylamine (20 equiv) then methanesulfonyl chloride (16 equiv). The mixture was warmed to room temperature and was stirred for 40 min. The reaction was quenched with water (10 mL) and extracted with dichloromethane (3 × 5 mL). The organic phase was dried with MgSO_4_, filtered, and concentrated under reduced pressure. The resulting product was used in the last step without further purification.

Step 3, cyclization: To the crude obtained in step 2 and dissolved in ethanol (20 mL) was added hydrochloric acid 37% in water (18 mL). Then, the solution was stirred for 12 h at 60 °C (**11c**) or 29 h at room temperature (**11e**). Ethanol was removed under reduced pressure, the crude was diluted with dichloromethane (40 mL) and washed with a saturated aqueous solution of sodium hydrogen carbonate until the acid was neutralized. The organic phase was dried with MgSO_4_, filtered, and concentrated under reduced pressure, and finally, the crude was purified by column chromatography on silica gel.

(**12c**) According to general procedure, the reaction of **11c** (553 mg, 0.580 mmol) afforded after the three steps and purification (eluent: EtOAc/PE 20/80) **12c** (302 mg, 0.329 mmol, 56%) as a yellow oil. ^1^H NMR (500 MHz, CDCl_3_): δ 7.37–7.28 (m, 30 H, Ar-H), 4.59–4.51 (m, 10 H, 4 × C*H_2_*-Ph + 2 × C*H_a_*H_b_-Ph), 4.47 (d, *J* = 12.3 Hz, 2 H, 2 × CH_a_*H_b_*Ph), 3.94 (d, *J* = 5 Hz, 2 H, 2 × 3-H), 3.92 (d, *J* = 3.6 Hz, 2 H, 2 × 4-H), 3.62 (dd, *J* = 9.5, 4.8 Hz, 2 H, 2 × 6-*H_a_*H_b_), 3.55 (dd, *J* = 9.5, 6.9 Hz, 2 H, 2 × 6-H_a_*H_b_*), 3.25 (d, *J* = 10.3 Hz, 2 H, 2 × 2-*H_a_*H_b_), 2.88–2.82 (m, 2 H, 2 × N-C*H_a_*H_b_-CH_2_), 2.75–2.72 (m, 2 H, 2 × 5-H), 2.58 (dd, *J* = 10.4, 5.0 Hz, 2 H, 2 × 2-H_a_*H_b_*), 2.37–2.31 (m, 2 H, 2 × N-CH_a_*H_b_*-CH_2_), 1.54–1.48 (m, 4H, 2 × N-CH_2_-C*H_2_*), 1.31–1.28 (m, 8H, 4 × CH_2_) ppm; ^13^C NMR (125 MHz, CDCl_3_): δ 138.6 (C^IV^), 138.4 (C^IV^), 138.4 (C^IV^), 128.5, 128.5, 128.4, 128.4, 128.4, 127.9, 127.9, 127.9, 127.7, 127.6 (C-Ar), 85.6 (C-4), 81.7 (C-3), 73.3 (*C*H_2_-Ph), 71.4 (*C*H_2_-Ph), 71.1 (C-6), 71.0 (*C*H_2_-Ph), 69.5 (C-5), 57.4 (C-2), 55.8 (N-*C*H_2_-CH_2_), 29.7 (CH_2_), 28.3 (N-CH_2_-*C*H_2_), 27.7 (CH_2_) ppm; ${\left[\alpha \right]}_{D}^{25}$ = −26.6 (*c* 1, CHCl_3_). HRMS (ESI) *m*/*z* calcd for [C_60_H_72_N_2_O_6_ + H]^+^: 917.5469, found 917.5472.

(**12e**) According to general procedure, the reaction of **11e** (1.7 g, 1.672 mmol) afforded after the three steps and purification (eluent: EtOAc/PE gradient, 50/50 to 100/00) **12e** (349 mg, 0.352 mmol, 21%) as a yellow oil. ^1^H NMR (500 MHz, CDCl_3_): δ 7.37–7.27 (m, 30 H, Ar-H), 4.57–4.49 (m, 10 H, 4 × C*H_2_*-Ph + 2 × C*H_a_*H_b_-Ph), 4.45 (d, *J* = 12.3 Hz, 2 H, 2 × CH_a_*H_b_*Ph), 3.94 (d, *J* = 5.1 Hz, 2 H, 2 × 3-H), 3.91 (d, *J* = 3.9 Hz, 2 H, 2 × 4-H), 3.65–3.47 (m, 16 H, 6 × CH_2_ + 2 × 6-H), 3.23 (d, *J* = 10.3 Hz, 2 H, 2 × 2-*H_a_*H_b_), 2.95 (dt, *J* = 11.9, 8.0 Hz, 2 H, 2 × N-C*H_a_*H_b_-CH_2_), 2.76–2.73 (m, 2 H, 2 × 5-H), 2.59 (dd, *J* = 10.4, 5.1 Hz, 2 H, 2 × 2-H_a_*H_b_*), 2.43 (dt, *J* = 12.4, 6.7 Hz, 2 H, 2 × N-CH_a_*H_b_*-CH_2_), 1.80 (dq, *J* = 14.4, 7.0 Hz, 4 H, 2 × N-CH_2_-C*H_2_*) ppm; ^13^C NMR (125 MHz, CDCl_3_): δ 138.6 (C^IV^), 138.4 (C^IV^), 138.4 (C^IV^), 128.5, 128.5, 128.4, 128.0, 127.9, 127.9, 127.8, 127.8, 127.8, 127.7, 127.7, 127.6 (C-Ar), 85.6 (C-4), 81.7 (C-3), 73.3 (*C*H_2_-Ph), 71.4 (*C*H_2_-Ph), 71.1 (C-6 + *C*H_2_-Ph), 70.7 (CH_2_), 70.3 (CH_2_), 69.8 (CH_2_), 69.5 (C-5), 57.3 (C-2), 52.4 (N-*C*H_2_-CH_2_), 28.4 (N-CH_2_-*C*H_2_) ppm; ${\left[\alpha \right]}_{D}^{25}$ = −29.3 (*c* 1, CHCl_3_). HRMS (ESI) *m*/*z* calcd for [C_62_H_76_N_2_O_9_ + H]^+^: 993.5629, found 993.5627.

### 3.6. Representative Procedure for the Synthesis of Deprotected Bis-Iminosugars ***13a***–***e***

To a solution of dimeric iminosugars **12a**–**e** (1 equiv) in anhydrous dichloromethane at 0 °C and under argon atmosphere was added dropwise boron trichloride solution 1 M in dichloromethane (10 equiv). The mixture was stirred for 2 h 30 at the same temperature and then was quenched with methanol (3 mL). The solvent was removed under reduced pressure and the residue was washed with diethyl ether (3 × 1 mL) and then chloroform (1.5 mL). Finally, the crude was purified by column chromatography on silica gel (**13a**–**c**,**e**) or passed through an ion exchange resin (DOWEX 50WX8 (NH_4_^+^) column (Sigma-Aldrich: St. Louis, MO, USA), elution with a solution of 0.2% ammonium bicarbonate) and lyophilized (**13d**).

(**13a**) According to general procedure, the reaction of **12a** (57 mg, 0.063 mmol) and boron trichloride solution 1 M in dichloromethane (630 µL, 0.630 mmol) in dichloromethane (0.5 mL) afforded after purification (eluent: CHCl_3_/MeOH/NH_4_OH 0.8 M 60/40/10) **13a** (8.3 mg, 0.023 mmol, 36%) as a yellow oil. ^1^H NMR (500 MHz, D_2_O): δ 7.55–7.54 (m, 4 H, Ar-H), 4.38 (d, *J* = 12.7 Hz, 2 H, 2 × N-C*H_a_*H_b_Ar), 4.21–4.20 (m, 2 H, 3-H), 4.10–4.05 (m, 4 H, N-CH_a_*H_b_*Ar + 4-H), 3.83 (dd, *J* = 12.1, 5.4 Hz, 2 H, 2 × 6-*H_a_*H_b_), 3.77 (dd, *J* = 12.1, 6.1 Hz, 2 H, 2 × 6-H_a_*H_b_*), 3.30–3.15 (m, 6 H, 2 × 2-H + 2 × 5-H) ppm; ^13^C NMR (125 MHz, D_2_O): δ 133.3 (C^IV^), 132.6, 131.3, 129.5 (C-Ar), 77.6 (C-4), 74.3 (C-3), 72.8 (C-5), 59.6 (C-6), 58.9 (N-*C*H_2_-Ar), 58.3 (C-2) ppm; ${\left[\alpha \right]}_{D}^{25}$ = +3.5 (*c* 1, MeOH). HRMS (ESI) *m*/*z* calcd for [C_18_H_28_N_2_O_6_ + H]^+^: 369.2026, found 369.2025.

(**13b**) According to general procedure, the reaction of **12b** (115 mg, 0.127 mmol) and boron trichloride solution 1 M in dichloromethane (1.27 mL, 1.27 mmol) in dichloromethane (2 mL) afforded after purification (eluent: CHCl_3_/MeOH gradient, 75/25 to 60/40) **13b** (18 mg, 0.049 mmol, 39%) as a yellow oil. ^1^H NMR (500 MHz, D_2_O): δ 7.61 (s, 4 H, Ar-H), 4.57 (d, *J* = 12.8 Hz, 2 H, 2 × N-C*H_a_*H_b_Ar), 4.34 (d, *J* = 12.8 Hz, 2 H, 2 × N-CH_a_*H_b_*Ar), 4.29–4.28 (m, 2 H, 3-H), 4.11–4.10 (m, 2 H, 4-H), 3.89 (dd, *J* = 12.3, 5.2 Hz, 2 H, 2 × 6-*H_a_*H_b_), 3.83 (dd, *J* = 12.3, 6.9 Hz, 2 H, 2 × 6-H_a_*H_b_*), 3.54–3.49 (m, 4 H, 2 × 2-*H_a_*H_b_ + 2 × 5-H), 3.37–3.34 (m, 2 H, 2 × 2-H_a_*H_b_*) ppm; ^13^C NMR (125 MHz, D_2_O): δ 132.2 (C^IV^), 131.6 (C-Ar), 76.7 (C-4), 73.8 (C-3), 73.7 (C-5), 59.0 (C-6), 58.9 (N-*C*H_2_-Ar), 58.5 (C-2) ppm; ${\left[\alpha \right]}_{D}^{25}$ = –1.4 (*c* 1, MeOH). HRMS (ESI) *m*/*z* calcd for [C_18_H_28_N_2_O_6_ + H]^+^: 369.2026, found 369.2023.

(**13c**) According to general procedure, the reaction of **12c** (117 mg, 0.127 mmol) and boron trichloride solution 1 M in dichloromethane (1.27 mL, 1.27 mmol) in dichloromethane (2 mL) afforded after purification (eluent: CHCl_3_/MeOH gradient, 70/30 to 50/50) **13c** (42 mg, 0.110 mmol, 87%) as a yellow oil. ^1^H NMR (500 MHz, D_2_O): δ 4.36–4.35 (m, 2 H, 3-H), 4.12–4.11 (m, 2 H, 4-H), 4.01 (dd, *J* = 12.5, 4.9 Hz, 2 H, 2 × 6-*H_a_*H_b_), 3.94 (dd, *J* = 12.5, 7.6 Hz, 2 H, 2 × 6-H_a_*H_b_*), 3.68 (d, *J* = 12.4 Hz, 2 H, 2 × 2-*H_a_*H_b_), 3.55–3.44 (m, 6 H, 2 × 2-H_a_*H_b_* + 2 × 5-H + 2 × N-C*H_a_*H_b_-CH_2_), 3.24–3.17 (m, 2 H, 2 × N-CH_a_*H_b_*-CH_2_), 1.78–1.72 (m, 4 H, 2 × N-CH_2_-C*H_2_*), 1.40–1.37 (m, 8 H, 4 × CH_2_) ppm; ^13^C NMR (125 MHz, D_2_O): δ 78.4 (C-4), 77.7 (C-5), 76.3 (C-3), 61.1 (C-2), 61.0 (C-6), 59.4 (N-*C*H_2_-CH_2_), 30.4 (CH_2_), 28.1 (CH_2_), 27.1 (N-CH_2_-*C*H_2_) ppm; ${\left[\alpha \right]}_{D}^{25}$ = −18.9 (*c* 0.45, H_2_O). HRMS (ESI) *m*/*z* calcd for [C_18_H_36_N_2_O_6_ + H]^+^: 377.2652, found 377.2650.

(**13d**) According to general procedure, the reaction of **12d** (90 mg, 0.098 mmol) and boron trichloride solution 1 M in dichloromethane (980 µL, 0.98 mmol) in dichloromethane (1 mL) afforded after purification **13d** (13 mg, 0.034 mmol, 35%) as a yellow oil. ^1^H NMR (500 MHz, D_2_O): δ 4.11 (dt, *J* = 5.4, 2.5 Hz, 2 H, 2 × 3-H), 3.92 (dd, *J* = 5.0, 2.8 Hz, 2 H, 2 × 4-H), 3.72–3.63 (m, 12 H, 2 × 6-H + 4 × CH_2_), 3.11–3.04 (m, 4 H, 2 × 2-*H_a_*H_b_ + 2 × N-C*H_a_*H_b_-CH_2_), 2.83–2.79 (m, 2 H, 2 × 2-H_a_*H_b_*), 2.64–2.61 (m, 2 H, 2 × N-CH_a_*H_b_*-CH_2_), 2.58 (q, *J* = 5.0 Hz, 2 H, 2 × 5-H) ppm; ^13^C NMR (125 MHz, D_2_O): δ 78.8 (C-4), 75.4 (C-3), 72.0 (C-5), 69.4 (CH_2_), 68.6 (CH_2_), 60.9 (C-6), 58.8 (C-2), 53.8 (N-*C*H_2_-CH_2_) ppm; ${\left[\alpha \right]}_{D}^{25}$ = −14.6 (*c* 1, MeOH). HRMS (ESI) *m*/*z* calcd for [C_16_H_32_N_2_O_8_ + H]^+^: 381.2237, found 381.2236.

(**13e**) According to general procedure, the reaction of **12e** (277 mg, 0.279 mmol) and boron trichloride solution 1 M in dichloromethane (2.79 mL, 2.79 mmol) in dichloromethane (3 mL) afforded after purification (eluent: EtOAc/MeOH/NH_4_OH 0.8 M 50/50/5) **13e** (78 mg, 0.173 mmol, 62%) as a yellow oil. ^1^H NMR (500 MHz, D_2_O): δ 4.33–4.32 (m, 2 H, 2 × 3-H), 4.10–4.09 (m, 2 H, 2 × 4-H), 3.98 (dd, *J* = 12.4, 4.9 Hz, 2 H, 2 × 6-*H_a_*H_b_), 3.91 (dd, *J* = 12.4, 6.8 Hz, 2 H, 2 × 6-H_a_*H_b_*), 3.70–3.61 (m, 14 H, 2 × 2-*H_a_*H_b_ + 6 × CH_2_), 3.52–3.56 (m, 2 H, 2 × N-C*H_a_*H_b_-CH_2_), 3.41 (dd, *J* = 12.1, 4.4 Hz, 2 H, 2 × 2-H_a_*H_b_*), 3.36–3.35 (m, 2 H, 2 × 5-H), 3.21–3.17 (m, 2 H, 2 × N-CH_a_*H_b_*-CH_2_), 2.10–1.95 (m, 4 H, 2 × N-CH_2_-C*H_2_*) ppm; ^13^C NMR (125 MHz, D_2_O): δ 76.5 (C-4), 74.7 (C-5), 74.0 (C-3), 69.5 (CH_2_), 69.5 (CH_2_), 68.4 (CH_2_), 58.7 (C-6), 58.6 (C-2), 54.2 (N-*C*H_2_-CH_2_), 25.1 (N-CH_2_-*C*H_2_) ppm; ${\left[\alpha \right]}_{D}^{25}$ = –5.7 (*c* 0.37, H_2_O). HRMS (ESI) *m*/*z* calcd for [C_20_H_40_N_2_O_9_ + H]^+^: 453.2812, found 453.2814.

### 3.7. Procedure for Allylation of Bis-Glycosylamine ***10d***

To a solution of dry lithium chloride (650 mg, 15.24 mmol, 6 equiv) in anhydrous tetrahydrofuran (10 mL) at −78 °C and under argon atmosphere was added dropwise a solution of allyl magnesium chloride 2 M in tetrahydrofuran (7.62 mL, 15.24 mmol, 6 equiv). The mixture was stirred for 20 min then a solution of the bis-glycosylamine **10d** (2.42 g, 2.54 mmol, 1 equiv) in anhydrous tetrahydrofuran (20 mL) was added dropwise. The mixture was stirred at −78 °C for 30 min then was warmed at room temperature and stirred until total consumption of starting material (typically 2 h). The reaction was quenched with a saturated aqueous solution of ammonium chloride and extracted with ethyl acetate (3 × 30 mL). The organic phase was dried with magnesium sulfate, filtered, and concentrated under reduced pressure. The crude was purified by column chromatography on silica gel (eluent: EtOAC/MeOH 97:3) to afford a mixture of **14d(*R,R*)** and **14d(*R,S*)** (85/15) (2.24 g, 2.16 mmol, 85%) as a yellow oil.

(**14d**) ^1^H NMR (500 MHz, CDCl_3_): δ 7.37–7.22 (m, 30 H, Ar-H), 5.80–5.72 (m, 1.7 H, 2 × 2-H^maj^), 5.60–5.51 (m, 0.3 H, 2 × 2-H^min^), 5.14–5.09 (m, 3.4 H, 2 × 1-H^maj^), 5.02 (d, *J* = 10.2 Hz, 0.3 H, 2 × 1-*H_a_*H_b_^min^), 4.90 (d, *J* = 17 Hz, 0.3 H, 2 × 1-H_a_*H_b_*^min^), 4.68–4.32 (m, 12 H, 6 × C*H_2_*-Ph^maj^ + 6 × C*H_2_*-Ph^min^), 4.16–4.13 (m, 0.3 H, 2 × 6-H^min^), 4.08 (d, *J* = 6.7 Hz, 1.7 H, 2 × 6-H^maj^), 3.83–3.81 (m, 2 H, 2 × 7-H^maj^ +2 × 7-H^min^), 3.65–3.39 (m, 14 H, 2 × 5-H^maj^ + 2 × 5-H^min^ + 2 × 8-H^maj^ + 2 × 8-H^min^ + 4 × CH_2_^maj^ + 4 × CH_2_^min^), 3.06–2.93 (m, 4 H, 2 × 4-H^maj^ + 2 × 4-H^min^ + 2 × NH-C*H_a_*H_b_^maj^ + 2 × NH-C*H_a_*H_b_^min^), 2.81–2.77 (m, 1.7 H, 2 × NH-CH_a_*H_b_*^maj^), 2.56–2.45 (m, 4 H, 2 × 3-H^maj^ + 2 × 3-*H_a_*H_b_^min^ + 2 × NH-CH_a_*H_b_*^min^), 2.15–2.07 (m, 0.3 H, 2 × 3-H_a_*H_b_*^min^) ppm; ^13^C NMR (125 MHz, D_2_O): δ 138.7 (C^IV^), 138.6 (C^IV^), 138.6 (C^IV^), 138.5 (C^IV^), 138.4 (C^IV^), 138.3 (C^IV^), 138.3 (C^IV^), 138.3 (C^IV^), 138.1 (C^IV^), 138.0 (C^IV^), 137.9 (C^IV^), 135.7 (C-2^min^), 135.3 (C-2^maj^), 128.8, 128.7, 128.5, 128.4, 128.4, 128.3, 128.0, 127.9, 127.9, 127.8, 127.6, 127.6 (C-Ar), 118.2 (C-1^maj^), 117.7 (C-1^min^), 78.4 (C-7^maj^), 77.1 (C-5^min^), 75.1 (C-5^maj^ + C-7^min^), 73.9 (CH_2_-Ph^maj^), 73.6 (CH_2_-Ph^min^), 73.2 (CH_2_-Ph^min^), 73.2 (CH_2_-Ph^maj^), 73.0 (CH_2_-Ph^maj^), 72.8 (CH_2_-Ph^min^), 71.1 (C-8^maj^), 70.8 (C-8^min^), 70.3 (CH_2_), 70.2 (CH_2_), 67.2 (C-6^maj^), 66.3 (C-6^min^), 58.9 (C-4^min^), 54.5 (C-4^maj^), 46.8 (NH-*C*H_2_^maj^), 45.6 (NH-*C*H_2_^min^), 34.6 (C-3^min^), 34.2 (C-3^maj^) ppm; HRMS (ESI) *m*/*z* calcd for [C_64_H_80_N_2_O_10_ + H]^+^: 1037.5891, found 1037.5898.

### 3.8. Procedure for the Synthesis of Bis-Allylpyrrolidines ***15d*(R,R)** and ***15d*(R,S)**

To a solution of the bis-glycosylamines **14d(*R,R*)** and **14d(*R,S*)** (2.24 g, 2.16 mmol, 1 equiv) in anhydrous tetrahydrofuran (10 mL) under argon atmosphere was added pyridine (10 mL). The mixture was cooled to −78 °C then methanesulfonyl chloride (0.84 mL, 10.81 mmol, 5 equiv) was added dropwise. The mixture was stirred for 1 h at the same temperature, and then was warmed to 0 °C and stirred for another 2 h. The reaction was quenched with water (10 mL) and extracted with ethyl acetate (4 × 40 mL). The organic phase was dried with magnesium sulfate, filtered, and concentrated under reduced pressure, and finally, the crude was purified by column chromatography on silica gel (eluent: PE/EtOAc 70/30) to afford **15d(*R,R*)** (731 mg, 0.731 mmol), **15d(*R,S*)** (100 mg, 0.1 mmol) and a mixture of the two diastereomers (869 mg, 0.869 mmol, **15d(*R,R*)**/**15d(*R,S*)**: 83/17)). Global yield 79% yellow oils.

(**15d(*R,R*)**) ^1^H NMR (500 MHz, CDCl_3_): δ 7.37–7.28 (m, 30 H, Ar-H), 5.83–5.74 (m, 2 H, 2 × 2a-H), 5.08–5.05 (m, 4 H, 2 × 3a-H), 4.57–4.41 (m, 12 H, 6 × C*H_2_*-Ph), 3.90 (s, 2 H, 2 × 4-H), 3.80 (s, 2 H, 2 × 3-H), 3.70–3.52 (m, 12 H, 2 × 6-H + 4 × CH_2_), 3.29–3.26 (m, 2 H, 2 × 5-H), 3.19–3.17 (m, 2 H, 2 × 2-H), 3.01–2.91 (m, 4 H, 2 × N-C*H_2_*), 2.54–2.49 (m, 2 H, 2 × 1a-*H_a_*H_b_), 2.23–2.17 (m, 2 H, 2 × 1a-H_a_*H_b_*) ppm; ^13^C NMR (125 MHz, D_2_O): δ 138.5 (C^IV^), 135.6 (C-2a), 128.4, 128.4, 128.4, 128.0, 127.9, 127.9, 127.8, 127.6, (C-Ar), 117.2 (C-3a), 85.7 (C-3), 85.6 (C-4), 73.4 (CH_2_-Ph), 71.4 (CH_2_-Ph), 71.3 (CH_2_-Ph), 70.6 (CH_2_), 70.5 (CH_2_), 69.5 (C-6), 66.1 (C-5), 65.7 (C-2), 46.7 (N-CH_2_), 32.1 (C-1a) ppm; ${\left[\alpha \right]}_{D}^{25}$ = −8.4 (*c* 1, MeOH). HRMS (ESI) *m*/*z* calcd for [C_64_H_76_N_2_O_8_ + H]^+^: 1001.5680, found 1001.5682.

(**15d(*R,S*)**) ^1^H NMR (500 MHz, CDCl_3_): δ 7.38–7.25 (m, 30 H, Ar-H), 5.82–5.68 (m, 2 H, 2a-H + 2a′-H), 5.10–5.04 (m, 3 H, 3a-H + 3a′-*H_a_*H_b_), 4.99 (dd, *J* = 10.2, 1.5 Hz, 1 H, 3a′-H_a_*H_b_*), 4.62–4.41 (m, 11 H, 5 × C*H_2_*-Ph + C*H_a_*H_b_-Ph), 4.32 (d, *J* = 11.7 Hz, 1 H, CH_a_*H_b_*-Ph), 3.89–3.88 (m, 2 H, 4-H + 4′-H), 3.79–3.77 (m, 2 H, 3-H + 3′-H), 3.69–3.51 (m, 11 H, 6-H + 6′-*H_a_*H_b_ + 4 × CH_2_), 3.43 (t, *J* = 9.3 Hz, 1 H, 6′-H_a_*H_b_*), 3.28–3.25 (m, 1 H, 5-H), 3.18–3.16 (m, 1 H, 2-H), 3.14–3.10 (m, 1 H, 5′-H), 3.09–3.05 (m, 1 H, 2′-H), 3.04–2.87 (m, 4 H, 2 × N-C*H_2_*), 2.53–2.43 (m, 2 H, 1a-*H_a_*H_b_ + 1a′-*H_a_*H_b_), 2.34–2.29 (m, 1 H, 1a′-H_a_*H_b_*), 2.23–2.16 (m, 1 H, 1a-H_a_*H_b_*) ppm; ^13^C NMR (125 MHz, D_2_O): δ 138.6 (C^IV^), 138.5 (C^IV^), 138.65 (C^IV^), 138.2 (C^IV^), 136.1 (C-2a′), 135.6 (C-2a), 128.5, 128.5, 128.4, 128.4, 128.4, 128.3, 128.3, 128.3, 128.2, 128.2, 128.2, 128.2, 128.0, 128.0, 127.9, 127.9, 127.9, 127.8, 127.8, 127.7, 127.7, 127.6, (C-Ar), 117.2 (C-3a), 116.5 (C-3a′), 85.7 (C-3), 85.6 (C-4), 82.3 (C-4′), 82.3 (C-3′), 73.4 (CH_2_-Ph), 73.2 (CH_2_′-Ph), 72.4 (C-6′), 71.7 (CH_2_′-Ph), 71.4 (CH_2_-Ph), 71.3 (CH_2_-Ph), 70.7 (CH_2_′-Ph), 70.5 (CH_2_), 70.2 (CH_2_), 69.7 (C-5′), 69.5 (C-6), 66.9 (C-2′), 66.1 (C-5), 65.7 (C-2), 53.1 (N-CH_2_′), 46.7 (N-CH_2_), 32.8 (C-1a′), 32.1 (C-1a) ppm; ${\left[\alpha \right]}_{D}^{25}$ = +1.9 (*c* 1, MeOH). HRMS (ESI) *m*/*z* calcd for [C_64_H_76_N_2_O_8_ + H]^+^: 1001.5680, found 1001.5682.

### 3.9. Procedure for the Synthesis of Deprotected Bis-Allylpyrrolidine ***16d*(R,R)**

According to general procedure described in 3.6, the reaction of **15d(*R,R*)** (27 mg, 0.027 mmol, 1 equiv) and boron trichloride solution (270 µL, 0.27 mmol) in anhydrous dichloromethane (3 mL) afforded after purification (eluent: AcOEt/MeOH/NH_4_OH 0.8 M 40/60/5) **16d(*R,R*)** (7 mg, 0.015 mmol, 56%) as a yellow oil.

(**16d(*R,R*)**) ^1^H NMR (600 MHz, D_2_O): δ 5.91–5.84 (m, 2 H, 2 × 2a-H), 5.19 (dd, *J* = 19.9, 13.8 Hz, 4 H, 2 × 3a-H), 3.96–3.94 (s, 2 H, 2 × 4-H), 3.91–3.89 (s, 2 H, 2 × 3-H), 3.80–3.74 (m, 4 H, 2 × 6-H), 3.70–3.63 (m, 8 H, 4 × CH_2_), 3.05 (dt, *J* = 8.7, 4.0 Hz, 2 H, 2 × 2-H), 2.99–2.91 (m, 6 H, 2 × 5-H + 2 × N-C*H_2_*), 2.43 (dt, *J* = 14.0, 4.9 Hz, 2 H, 2 × 1a-*H_a_*H_b_), 2.12 (dt, *J* = 14.0, 8.8 Hz, 2 × 1a-H_a_*H_b_*) ppm; ^13^C NMR (151 MHz, D_2_O): δ 135.1 (C-2a), 117.6 (C-3a), 79.7 (C-3), 79.1 (C-4), 69.6 (CH_2_), 68.7 (CH_2_), 68.2 (C-5), 66.2 (C-2), 59.7 (C-6), 45.7 (N-CH_2_), 31.4 (C-1a) ppm; ${\left[\alpha \right]}_{D}^{25}$ = −17.6 (*c* 0.10, H_2_O). HRMS (ESI) *m*/*z* calcd for [C_22_H_40_N_2_O_8_ + Na]^+^: 483.2682, found 483.2691.

### 3.10. Procedure for the Synthesis of ***19d*(R,R)** and ***19d*(R,S)**

To a solution of **14d(*R,R*)** and **14d(*R,S*)** (85/15) (150 mg, 0,145 mmol, 1 equiv) in anhydrous dichloromethane (10 mL) at 0 °C and under argon atmosphere was added dropwise boron trichloride solution 1 M in dichloromethane (1.45 mL, 1.45 mmol, 10 equiv). The mixture was stirred for 2 h 30 at the same temperature and then was quenched with methanol (2 mL). The solvent was removed under reduced pressure and the residue was washed with chloroform (3 × 1 mL) then dichloromethane (3 × 1 mL) to afford a mixture of **19d(*R,R*)** and **19(*R,S*)** (85/15) (55 mg, 0.112 mmol, 77%) as a yellow oil.

(**19d(*R,R*)**) ^1^H NMR (500 MHz, MeOD): δ 5.92–5.77 (m, 2 H, 2 × 2-H), 5.29 (d, *J* = 17.1 Hz, 2 H, 2 × 1-*H_a_*H_b_), 5.19 (d, *J* = 10.2 Hz, 2 H, 2 × 1-H_a_*H_b_*), 3.98 (dd, *J* = 5.3, 3.1 Hz, 2 H, 2 × 5-H), 3.87 (t, *J* = 3.3 Hz, 2 H, 2 × 6-H), 3.82–3.55 (m, 14 H, 2 × 7-H + 2 × 8-H + 4 × CH_2_), 3.52 (q, *J* = 6.5 Hz, 2 H, 2 × 4-H), 3.42–3.34 (m, 2 H, 2 × NH-C*H_a_*H_b_), 3.30–3.25 (m, 2 H, 2 × NH-CH_a_*H_b_*), 2.68–2.54 (m, 4 H, 2 × 3-H) ppm; ^13^C NMR (125 MHz, MeOD): δ 133.8 (C-2), 120.3 (C-1), 72.9 (C-7), 71.8 (C-6), 71.4 (CH_2_), 70.1 (C-5), 66.8 (CH_2_), 64.0 (C-8), 62.8 (C-4), 47.7 (NH-*C*H_2_), 33.3 (C-3) ppm; HRMS (ESI) *m*/*z* calcd for [C_22_H_44_N_2_O_10_ + H]^+^: 497.3074, found 497.3070.

### 3.11. Procedure for Ethynylation of Bis-Glycosylamine ***10d***

To a solution of trimethylsilylacetylene (1.65 mL, 11.6 mmol, 8 equiv) in anhydrous tetrahydrofuran (10 mL) at 0 °C and under argon atmosphere was added dropwise ethyl magnesium bromide 3 M in diethylether (3.9 mL, 11.6 mmol, 8 equiv). The mixture was stirred for 30 min at the same temperature then a solution of the bis-glycosylamine **10d** (1.38 g, 1.45 mmol, 1 equiv) in anhydrous tetrahydrofuran (20 mL) was added dropwise. The mixture was stirred at 0 °C for 30 min then was finally warmed at room temperature and stirred for 16 h. The reaction was quenched with a saturated aqueous solution of ammonium chloride and extracted with ethyl acetate (2 × 40 mL). The organic phase was dried with magnesium sulfate, filtered, and concentrated under reduced pressure. The crude was purified by column chromatography on silica gel (eluent: PE/EtOAc 70/30) to afford **20d** (605 mg, 0.53 mmol, 37%) as a mixture of two diastereoisomers (95:5) and a yellow oil.

(**20d**) ^1^H NMR (500 MHz, CDCl_3_) major diastereomer: δ 7.36–7.27 (m, 28 H, Ar-H), 7.18–7.16 (m, 2 H, Ar-H), 4.73 (d, *J* = 11.8 Hz, 2 H, 2 × C*H_a_*H_b_-Ph), 4.64 (d, *J* = 11.8 Hz, 2 H, 2 × CH_a_*H_b_*-Ph), 4.51 (d, *J* = 12.0 Hz, 2 H, 2 × C*H_a_*H_b_-Ph), 4.46 (d, *J* = 12.0 Hz, 2 H, 2 × CH_a_*H_b_*-Ph), 4.42 (d, *J* = 12.1 Hz, 2 H, 2 × C*H_a_*H_b_-Ph), 4.38 (d, *J* = 12.1 Hz, 2 H, 2 × CH_a_*H_b_*-Ph), 4.09 (dd, *J* = 8.1, 5.8 Hz, 2 H, 2 × 6-H), 3.81–3.79 (m, 4 H, 2 × 3-H + 2 × 4-H), 3.63–3.44 (m, 14 H, 2 × 5-H + 2 × 7-H + 4 × CH_2_), 3.22–3.17 (m, 2 H, 2 × NH-C*H_a_*H_b_), 2.62 (dt, *J* = 12.1, 4.2 Hz, 2 H, 2 × NH-CH_a_*H_b_*), 0.2 (s, 18 H, 2 × Si(CH_3_)_3_) ppm; ^13^C NMR (125 MHz, CDCl_3_) major diastereomer: δ 138.7 (C^IV^), 138.3 (C^IV^), 138.0 (C^IV^), 128.6, 128.5, 128.5, 128.4, 128.4, 128.3, 128.2, 128.0, 127.9, 127.9, 127.8, 127.8, 127.7, 127.5 (C-Ar), 106.5 (C^IV^, C≡*C*-Si), 89.0 (C^IV^, *C*≡C-Si), 79.7 (C-4), 76.4 (C-5), 74.2 (CH_2_-Ph), 73.7 (CH_2_-Ph), 73.1 (CH_2_-Ph), 70.6 (C-7), 70.2 (CH_2_), 70.0 (CH_2_), 65.9 (C-6), 48.3 (C-3), 46.4 (NH-*C*H_2_), 0.1 (Si(CH_3_)_3_) ppm; HRMS (ESI) *m*/*z* calcd for [C_68_H_88_N_2_O_10_Si_2_ + H]^+^: 1149.6056, found 1149.6057.

### 3.12. Procedure for the Synthesis of Bis-Ethynylpyrrolidine ***21d***

Step 1, *O*-mesylation and cyclization: according to the procedure described in 3.8, the reaction of **20d** (550 mg, 0.48 mmol) and methanesulfonyl chloride (0.19 mL, 2.4 mmol) in pyridine (3 mL) and THF (3 mL) afforded the cyclized intermediate (235 mg, 0.21 mmol, 44%) as a yellow oil after purification (eluent: PE/EtOAc 90/10).

Step 2, TMS removing: To a solution of the pyrrolidine obtained in step 1 (200 mg, 0.18 mmol, 1 equiv) in anhydrous tetrahydrofuran (10 mL) was added a solution of tetrabutylammonium fluoride 1 M in tetrahydrofuran (0.45 mL, 0.447 mmol, 2.5 equiv). The mixture was stirred for 3 h at room temperature and then was concentrated under reduced pressure. The crude product was purified by column chromatography on silica gel (eluent: EP/EtOAc 70:30) to afford **21d** (140 mg, 0.144 mmol, 69%) as a colorless oil.

(**21d**) ^1^H NMR (500 MHz, CDCl_3_): δ 7.39–7.26 (m, 30 H, Ar-H), 4.64–4.58 (m, 6 H, 2 × CH_2_Ph + 2 × C*H_a_*H_b_-Ph), 4.54–4.45 (m, 6 H, 2 × CH_2_Ph + 2 × CH_a_*H_b_*-Ph), 3.90–3.89 (m, 4 H, 2 × 2-H + 2 × 3-H), 3.87–3.86 (m, 2 H, 2 × 4-H), 3.74–3.67 (m, 4 H, 2 × CH_2_), 3.63–3.53 (m, 8 H, 2 × CH_2_ + 6-H), 3.17–3.03 (m, 6 H, 2 × N-CH_2_ + 2 × 5-H), 2.39 (s, 2 H, 2 × C≡CH) ppm; ^13^C NMR (125 MHz, CDCl_3_): δ 138.5 (C^IV^), 138.1 (C^IV^), 138.0 (C^IV^), 128.4, 128.3, 128.3, 128.3, 128.0, 127.8, 127.8, 127.7, 127.6, 127.6, 127.6, 127.5 (C-Ar), 83.6 (C-4), 82.3 (C-3), 81.2 (C^IV^, *C*≡CH), 73.7 (C≡*C*H), 73.1 (CH_2_-Ph), 72.1 (CH_2_-Ph), 71.5 (C-6), 70.9 (CH_2_-Ph), 70.3 (CH_2_), 69.5 (CH_2_), 67.6 (C-5), 58.5 (C-2), 52.1 (N-*C*H_2_) ppm; ${\left[\alpha \right]}_{D}^{25}$ = −13.6 (*c* 1, CHCl_3_). HRMS (ESI) *m*/*z* calcd for [C_62_H_68_N_2_O_8_ + H]^+^: 969.5054, found 969.5056.

### 3.13. Procedure for the Synthesis of Glycophane ***22d***

To a solution of the bis-ethynylpyrrolidine **21d** (80 mg, 0.083 mmol, 1 equiv) in a mixture of ethanol (20 mL) and chloroform (5 mL) were successively added pyridine (340 μL, 0.42 mmol, 5 equiv), triethylamine (350 μL, 0.25 mmol, 3 equiv), copper(II) acetate monohydrate (17 mg, 0.083 mmol, 1 equiv) and nickel chloride (11 mg, 0.083 mmol, 1 equiv). The mixture was heated to 60 °C and stirred for 16 h. The solvents were removed under reduced pressure and the crude was purified by column chromatography on silica gel (eluent: EP/EtOAc 70/30) to afford **22d** (67 mg, 0.068 mmol, 82%) as a colorless oil.

(**22d**) ^1^H NMR (500 MHz, CDCl_3_): δ 7.37–7.27 (m, 26 H, Ar-H), 7.21–7.19 (m, 4 H, Ar-H), 4.65 (d, *J* = 12.5 Hz, 2 H, 2 × C*H_a_*H_b_-Ph), 4.57 (d, *J* = 12.5 Hz, 2 H, 2 × CH_a_*H_b_*-Ph), 4.54 (d, *J* = 12.0 Hz, 2 H, 2 × C*H_a_*H_b_-Ph), 4.47 (d, *J* = 12.0 Hz, 2 H, 2 × CH_a_*H_b_*-Ph), 4.41 (d, *J* = 12.1 Hz, 2 H, 2 × C*H_a_*H_b_-Ph), 4.38 (d, *J* = 12.1 Hz, 2 H, 2 × CH_a_*H_b_*-Ph), 3.85–3.82 (m, 4 H, 2 × 2-H + 2 × 3-H), 3.74 (dd, *J* = 3.3, 1.2 Hz, 2 H, 2 × 4-H), 3.60–3.48 (m, 6 H, 2 × CH_2_ + 2 × C*H_a_*H_b_), 3.60–3.50 (m, 6 H, 2 × 6-H + 2 × CH_a_*H_b_*), 3.09–3.04 (m, 2 H, 2 × N-C*H_a_*H_b_), 2.91–2.83 (m, 4 H, 2 × 5-H + 2 × N-CH_a_*H_b_*) ppm; ^13^C NMR (125 MHz, CDCl_3_): δ 138.4 (C^IV^), 138.0 (C^IV^), 138.0 (C^IV^), 128.5, 128.5, 128.5, 128.3, 127.9, 127.8, 127.8 (C-Ar), 83.9 (C-4), 82.5 (C-3), 78.0 (C^IV^, *C*≡C-C≡*C*), 73.3 (CH_2_-Ph), 72.3 (CH_2_-Ph), 71.7 (C-6), 71.4 (CH_2_-Ph), 71.0 (C^IV^, C≡*C*-*C*≡C), 70.9 (CH_2_), 70.0 (C-5), 69.7 (CH_2_), 60.6 (C-2), 54.5 (N-*C*H_2_) ppm; ${\left[\alpha \right]}_{D}^{25}$ = −84.2 (*c* 1, CHCl_3_). HRMS (ESI) *m*/*z* calcd for [C_62_H_67_N_2_O_8_ + H]^+^: 967.4897, found 967.4896.

### 3.14. Biological Assays Towards Glycosidases

All the enzymes were purchased from Sigma Chemical Co. In a typical experiment, the glycosidase (0.013 U/mL) was pre-incubated at 33 °C for 5 min in the presence of the inhibitor in 50 mM acetate buffer (pH 5.6, except for rice α-glucosidase pH 5.1 and yeast α-glucosidase pH 6.2). The reaction was started by the addition of the appropriate substrate (*p*-nitrophenyl glycoside, 1 mM concentration) to a final volume of 250 µL. The reaction was stopped after 10–15 min (depending on the enzyme) by the addition of 350 µL of 0.4 M Na_2_CO_3_. The released *p*-nitrophenolate was quantified spectrophotometrically at 415 nm with a microplate reader (300 µL of the reaction mixture per well). In cases where inhibition was greater than 95%, IC_50_ values were determined further after assaying decreasing concentrations of inhibitor. All the assays were performed in duplicate (less than 10% variability in each case).

## Figures and Tables

**Table 1:** Glycosidase inhibition potencies of prepared compounds ^a,b^.

Enzyme	13a	13b	13c	13d	13e	16d	DAB-1
α-glucosidase (rice)	40%	77%	92%	90%	72%	26%	>95% 7.8 μM
α-glucosidase (*Sac. cerevisiae*)	88%	95% 39 μM	88%	94%	65%	–20% ^c^	>95% 0.69 μM
β-glucosidase (almond)	13%	16%	60%	24%	23%	25%	95% 79 μM
β-glucosidase (*Asp. niger*)	24%	43%	94%	93%	91%	51%	>95% 62 μM
β-galactosidase (*Asp. orizae*)	93%	>95% 2.8 μM	>95% 31 μM	92%	81%	58%	76%
α-mannosidase (Jack bean)	75%	84%	>95% 18 μM	82%	>95% 34 μM	32%	>95% 49 μM
α-rhamnosidase (*Asp. niger*)	67%	24%	69%	46%	18%	25%	34%
α-galactosidase (green coffee)	38%	NI	61%	22%	22%	NI	11%
β-GlcNAc-ase (Jack bean)	NI	NI	NI	–38% ^c^	NI	–33% ^c^	14%

^a^ % inhibition at 1 mM concentration of inhibitor; IC_50_ in µM, determined when >95% inhibition. ^b^ NI means no impact on enzyme activity (less than 5% inhibition or activation). ^c^ negative values mean activation of enzyme activity.

## Data Availability

The data that support the findings of this study are available in the Supplementary Materials of this article.

## Supplementary Materials

The following supporting information can be downloaded at: https://www.mdpi.com/article/10.3390/molecules30020226/s1, analytical data of isomerized compound **18d**, ^1^H NMR and ^13^C NMR spectra of compounds **10a**–**e**, **11a**–**e**, **12a**–**e**, **13a**–**e**, **14d**, **15d(*R*,*R*)** (additional NOESY), **15d(*R*,*S*)**, **16d(*R*,*R*)**, **18d**, **19d**, **20d**, **21d** (additional NOESY), **22d** are available.
