# Supplementary material for: A Bis-Glycosylamine Strategy for the Synthesis of Dimeric Iminosugars Based on a DAB-1 Scaffold
Source: Molecules. 2025 Jan 8;30(2):226. doi: 10.3390/molecules30020226 (PMC11767490; doi:10.3390/molecules30020226)
Supplement: Supplementary file 1 [file molecules-30-00226-s001.zip › molecules-3390264-supplementary.pdf]

molecules

MDPI

Supplementary Material

A bis-glycosylamine strategy for the synthesis of dimeric iminosugars based on a DAB-1 scaffold

Kamilia Ould Lamara [1], Nathan Noël [1], Fabien Massicot [1], Jean-Luc Vasse [1], Stéphane P. Vincent [2] and Jean-Bernard Behr [1,*]

[1] Université de Reims Champagne-Ardenne, CNRS, ICMR, 51097 Reims, France; kamilia.ouldlamara@gmail.com (K.O.L.); nathan.noel@univ-reims.fr (N.N.); fabien.massicot@univ-reims.fr (F.M.); jean-luc.vasse@univ-reims.fr (J.-L.V.)

[2] NARILIS (Namur Research Institute for Life Sciences), UNamur, Rue de Bruxelles 61, 5000 Namur, Belgium; stephane.vincent@unamur.be

* Correspondence: jb.behr@univ-reims.fr

Content :

1. Analytical data of **18d** (page S2)

2. ^{1}H NMR and ^{13}C NMR spectra of new compounds (pages S3-S34)

3. Comparative ^{1}H NMR and ^{13}C NMR data of **19d(*R*,*R*)** and **19d(*S*,*S*)** (pages S35)

molecules

1. Characterizations of 18d

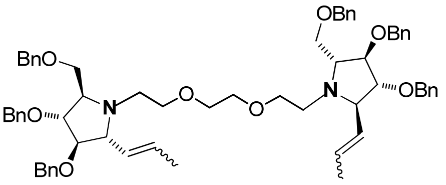

^1H NMR (500 MHz, $CDCl_3$): δ 7.34 – 7.29 (m, 30 H, Ar-H), 5.73 – 5.64 (m, 2 H, 2 x 1a-H^{maj} + 2 x 1a-H^{min}), 5.4 – 5.45 (m, 2 H, 2 x 2a-H^{maj} + 2 x 2a-H^{min}), 4.58 – 4.46 (m, 12 H, 6 x CH_2-Ph), 4.00 – 3.94 (m, 2 H, 2 x 4-H^{maj} + 2 x 4-H^{min}), 3.79 – 3.76 (m, 2 H, 2 x 3-H^{maj} + 2 x 3-H^{min}), 3.68 – 3.50 (m, 14 H, 2 x 6-H^{maj} + 2 x 6-H^{min} + 2 x 2-H^{maj} + 2 x 2-H^{min} + 4 x CH_2), 3.35 – 3.31 (m, 2 H, 2 x 5-H^{maj} + 2 x 5-H^{min}), 2.91 – 2.81 (m, 4 H, 2 x NH-CH_2^{maj} + 2 x NH-CH_2^{min}), 1.74 – 1.70 (m, 6 H, 2 x 3a-H^{maj} + 2 x 3a-H^{min}) ppm ; ^{13}C NMR (125 MHz, $CDCl_3$): δ 138.5 (C^{IV}), 138.5 (C^{IV}), 130.8 (C-2a^{maj}), 130.1 (C-2a^{min}), 129.6 (C-1a^{maj}), 128.4, 128.3, 127.9, (C-Ar), 127.9 (C-1a^{min}), 127.8, 127.7, 127.7, 127.6 (C-Ar), 88.5 (C-3^{min}), 88.2 (C-3^{maj}), 85.8 (C-4^{min}), 85.6 (C-4^{maj}), 73.3 (CH_2-Ph), 71.7 (CH_2-Ph), 71.4 (CH_2-Ph), 70.4 (CH_2), 70.2 (CH_2), 70.0 (C-2^{maj} + C-2^{min}), 69.1 (C-6^{min}), 68.9 (C-6^{maj}), 65.6 (C-5^{min}), 65.5 (C-5^{maj}), 46.8 (NH-CH_2^{min}), 46.7 (NH-CH_2^{maj}), 18.0 (C-3a^{maj}), 13.4 (C-3a^{min}) ppm; $[\alpha]_D^{25}$ = –30.8 (*c* 1, $CHCl_3$). HRMS (ESI) m/z calcd for $[C_{64}H_{76}N_2O_8 + H]^+$: 1001.5680, found 1001.5671.

molecules

MDPI

1. ^{1}H NMR and ^{13}C NMR spectra of new compounds

^{1}H-NMR spectrum of 10a ($CDCl_3$)

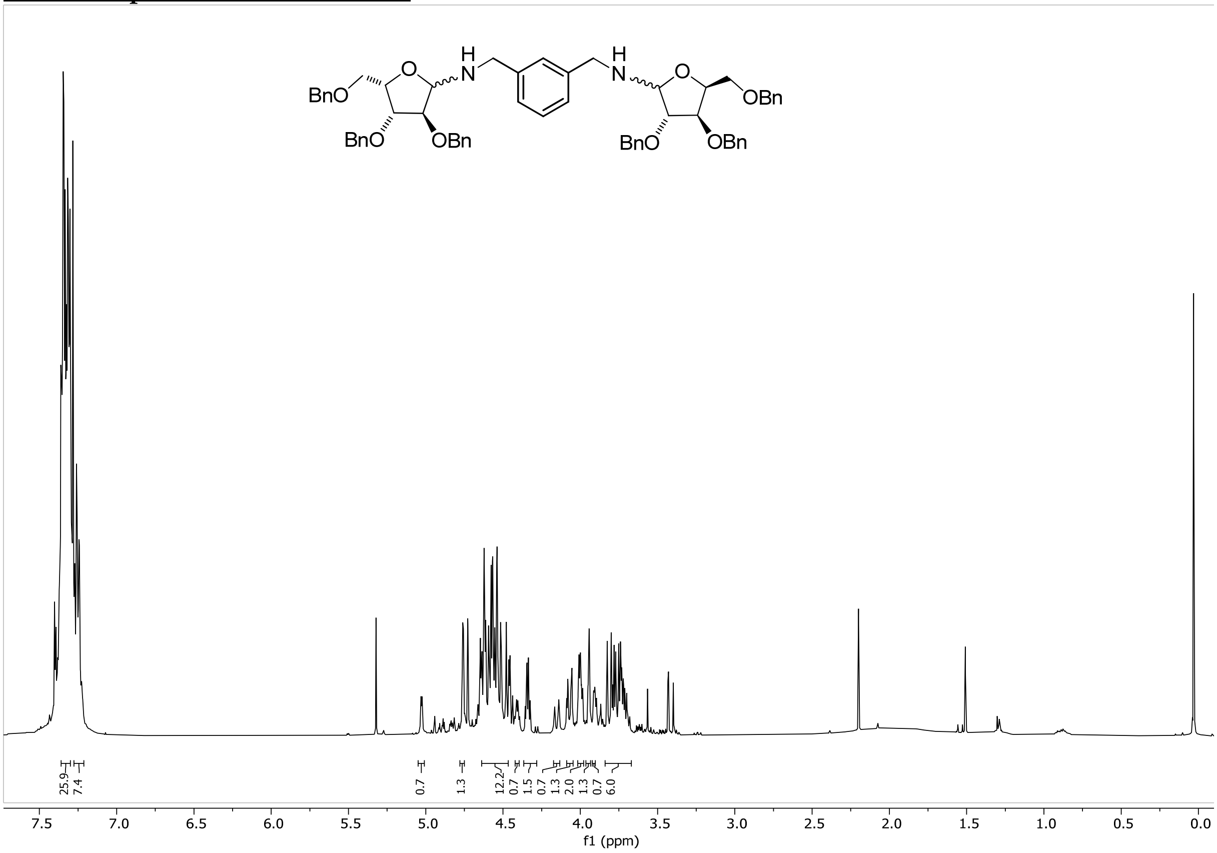

^{13}C-NMR spectrum of 10a ($CDCl_3$)

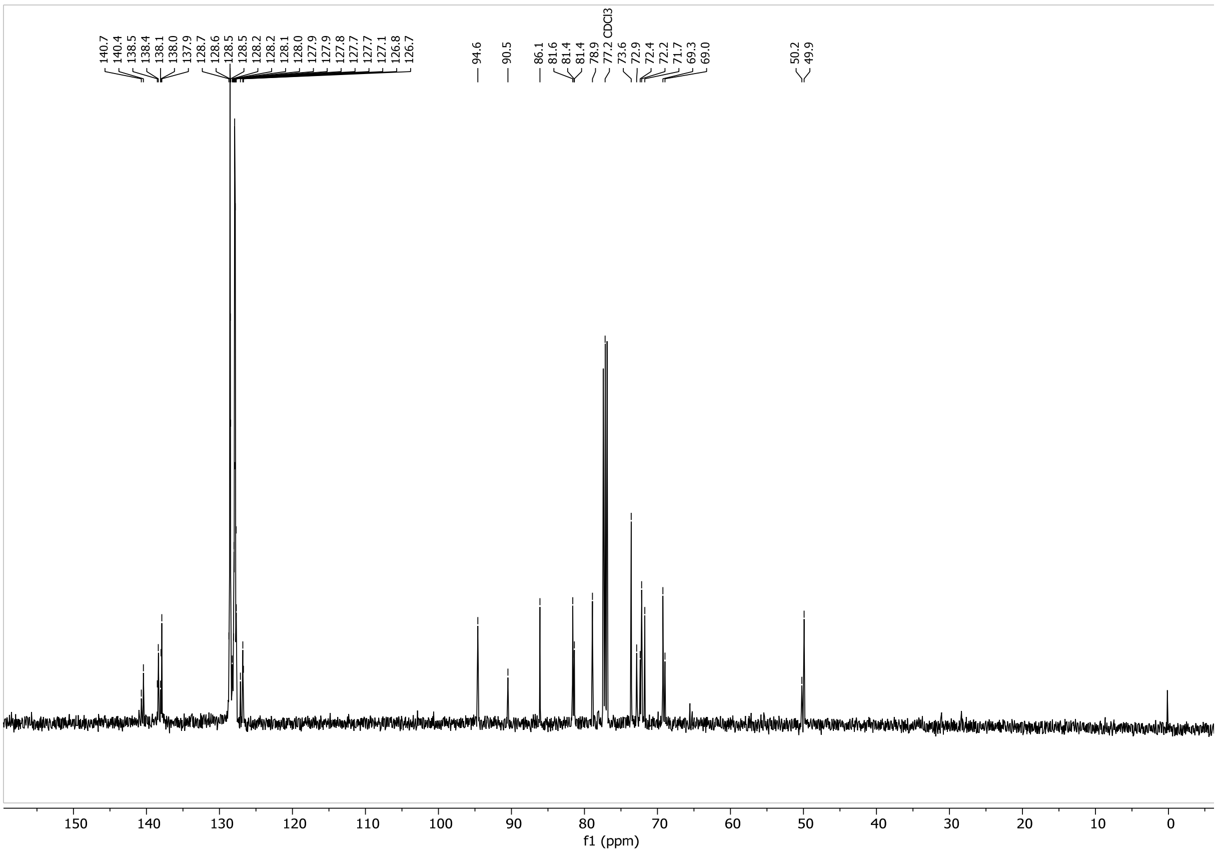

^{1}H-NMR spectrum of 10b ($CDCl_3$)

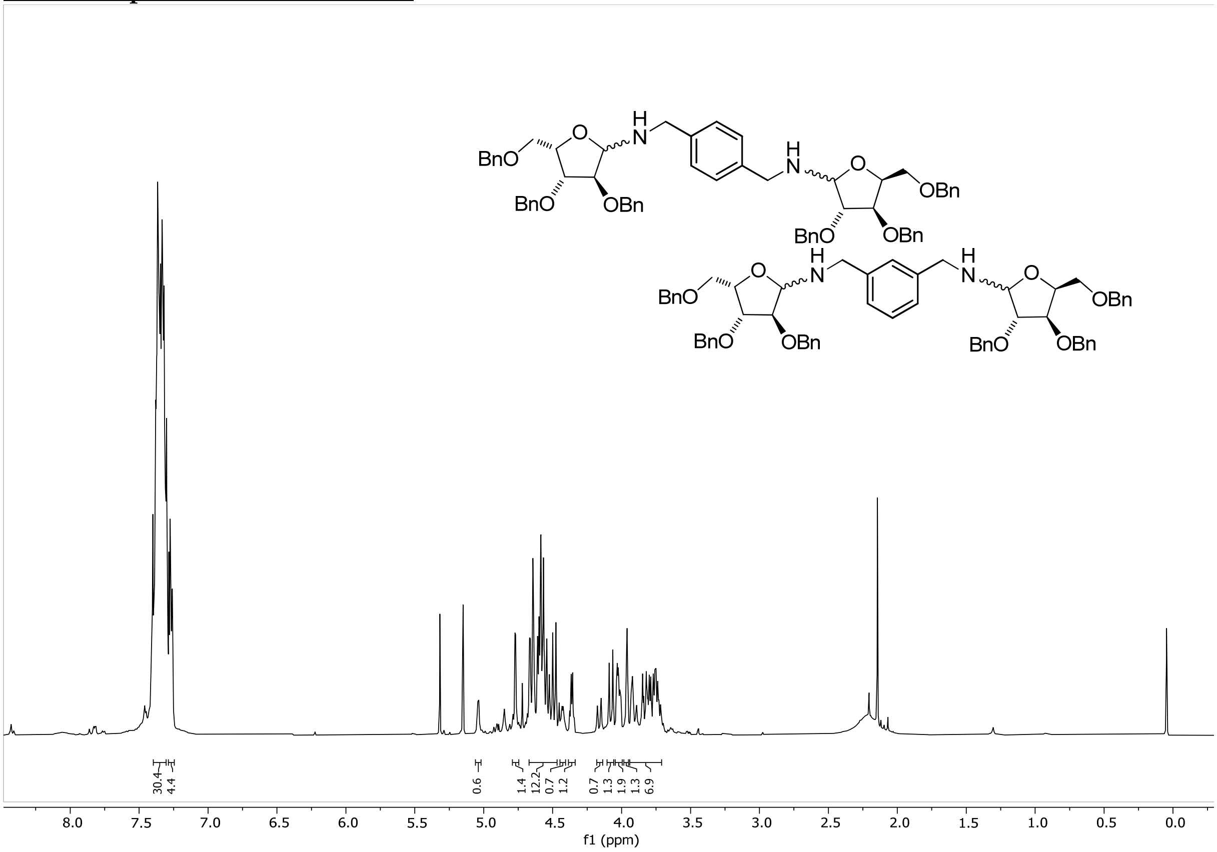

^{13}C-NMR spectrum of 10b ($CDCl_3$)

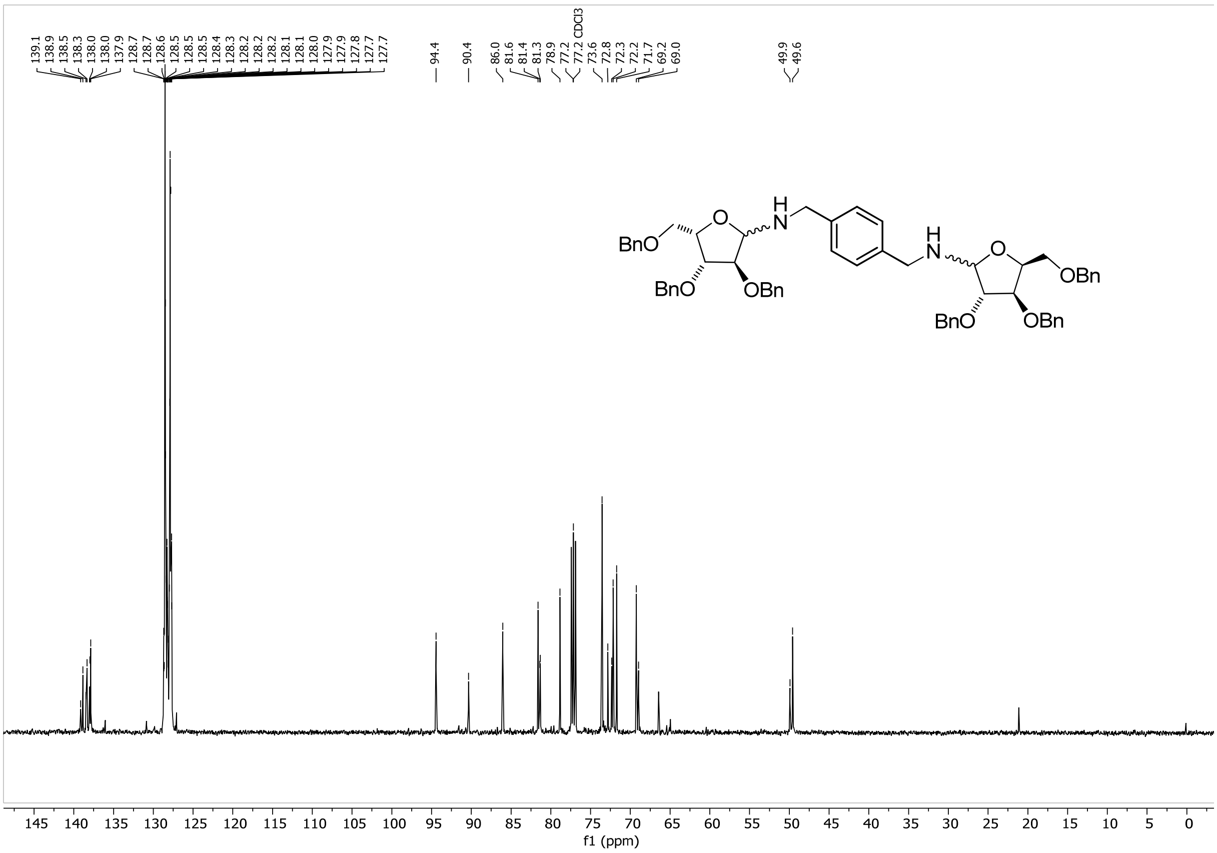

^{1}H-NMR spectrum of 10c (CDCl3)

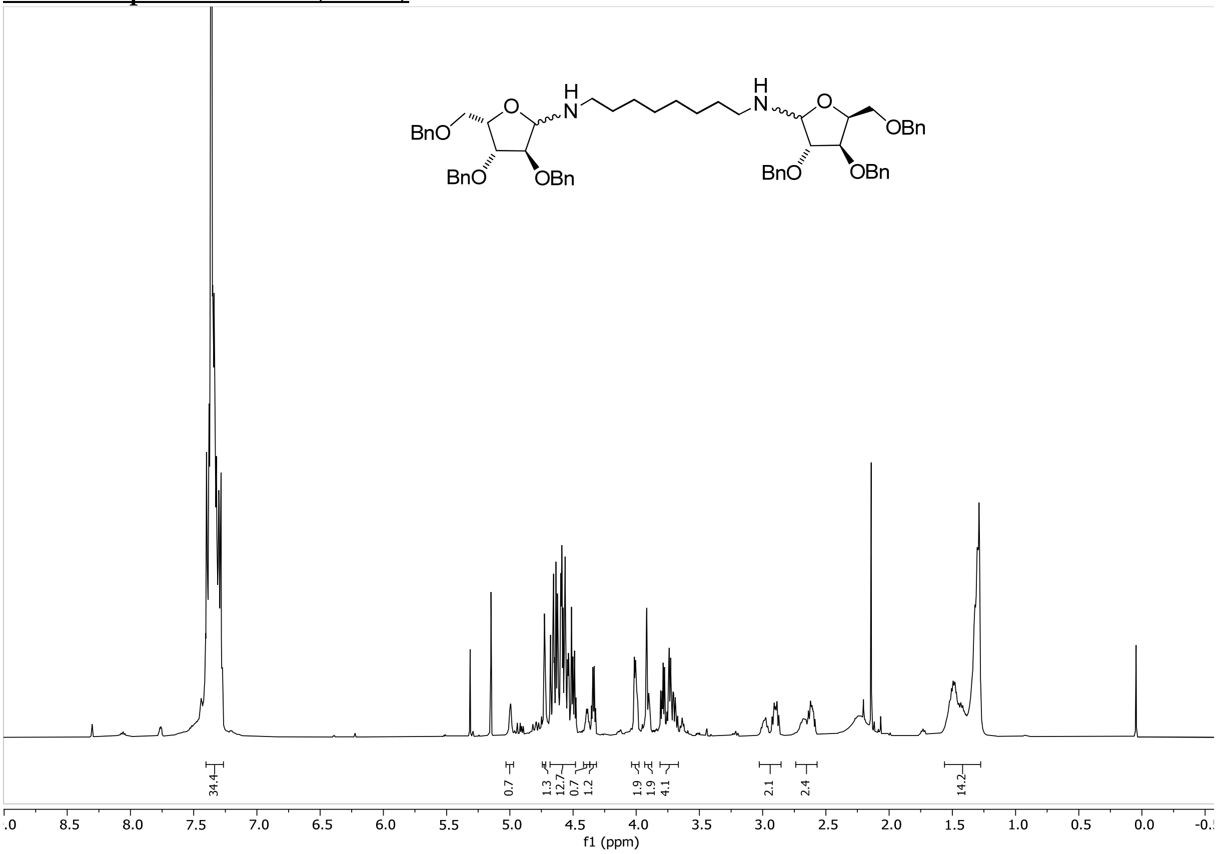

^{13}C-NMR spectrum of 10c (CDCl3)

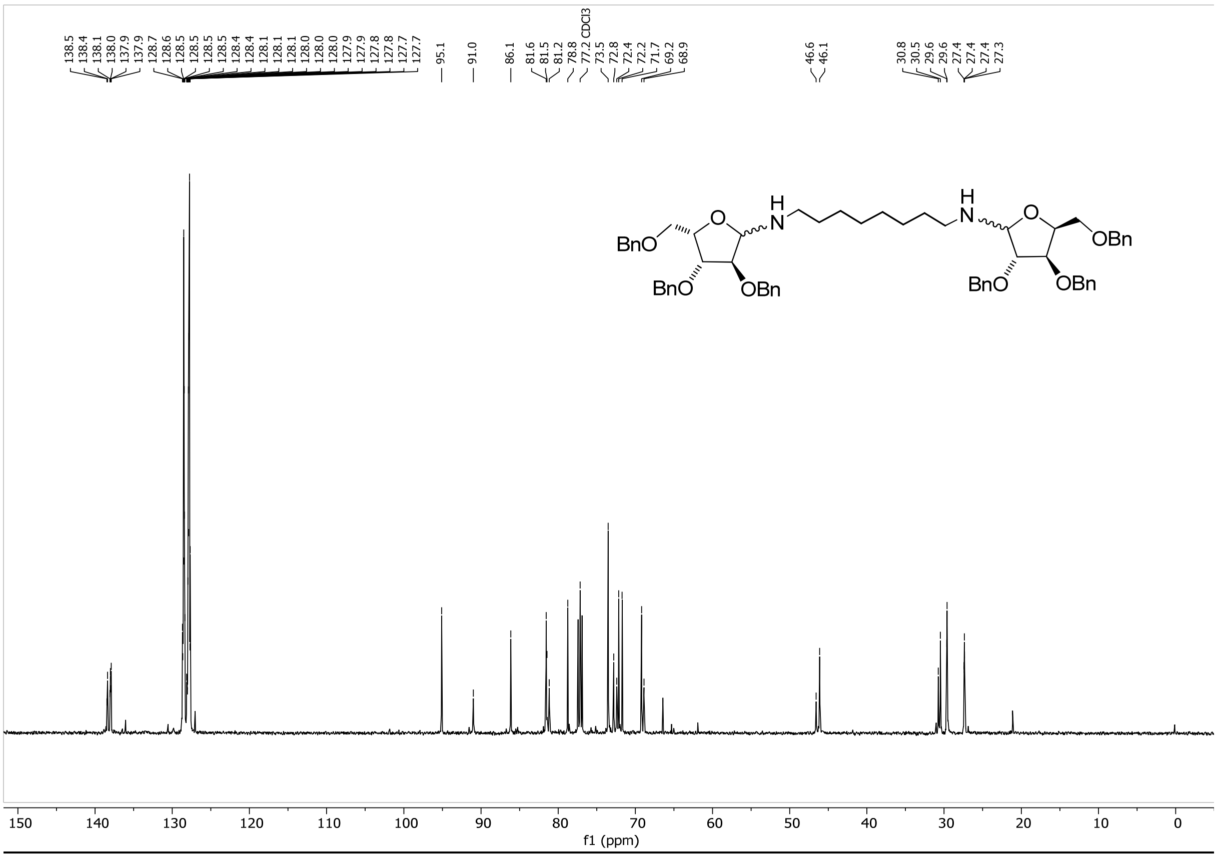

^{1}H-NMR spectrum of 10d ($CDCl_3$)

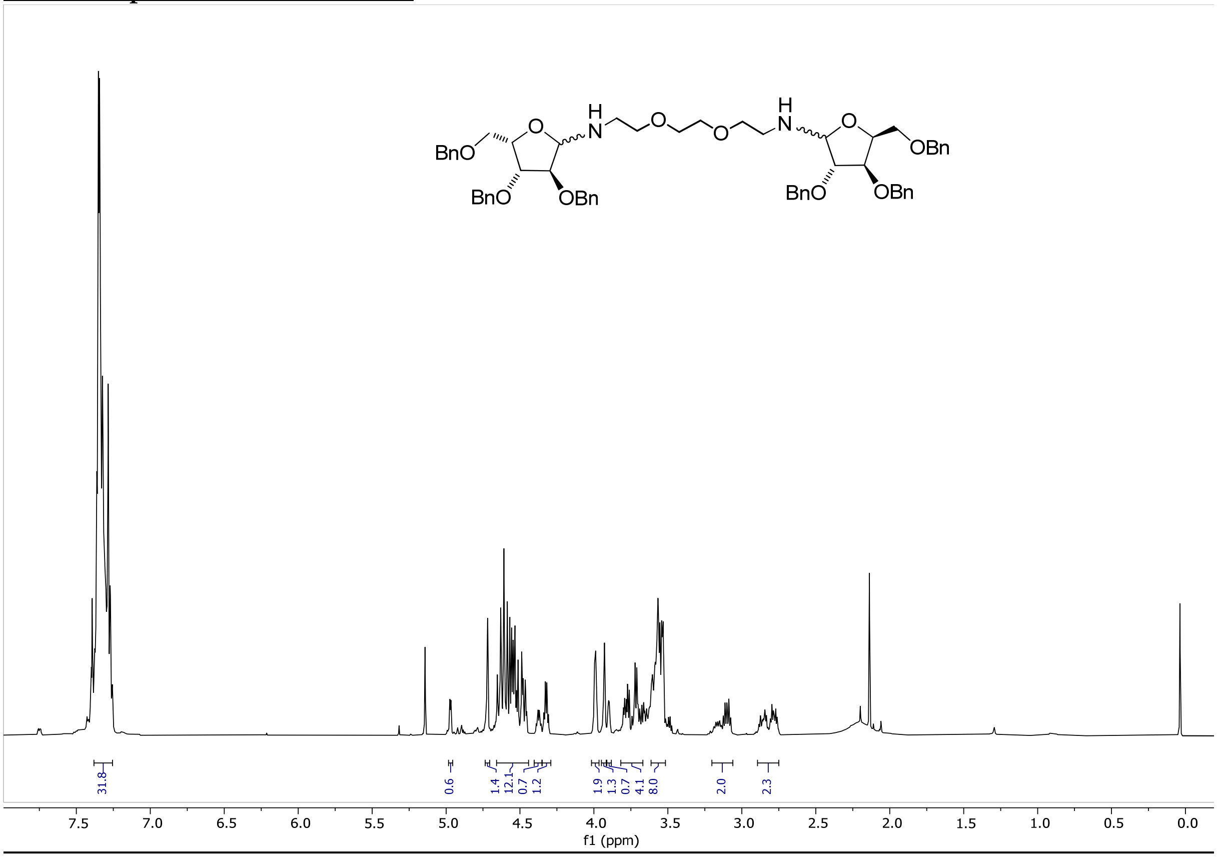

^{13}C-NMR spectrum of 10d ($CDCl_3$)

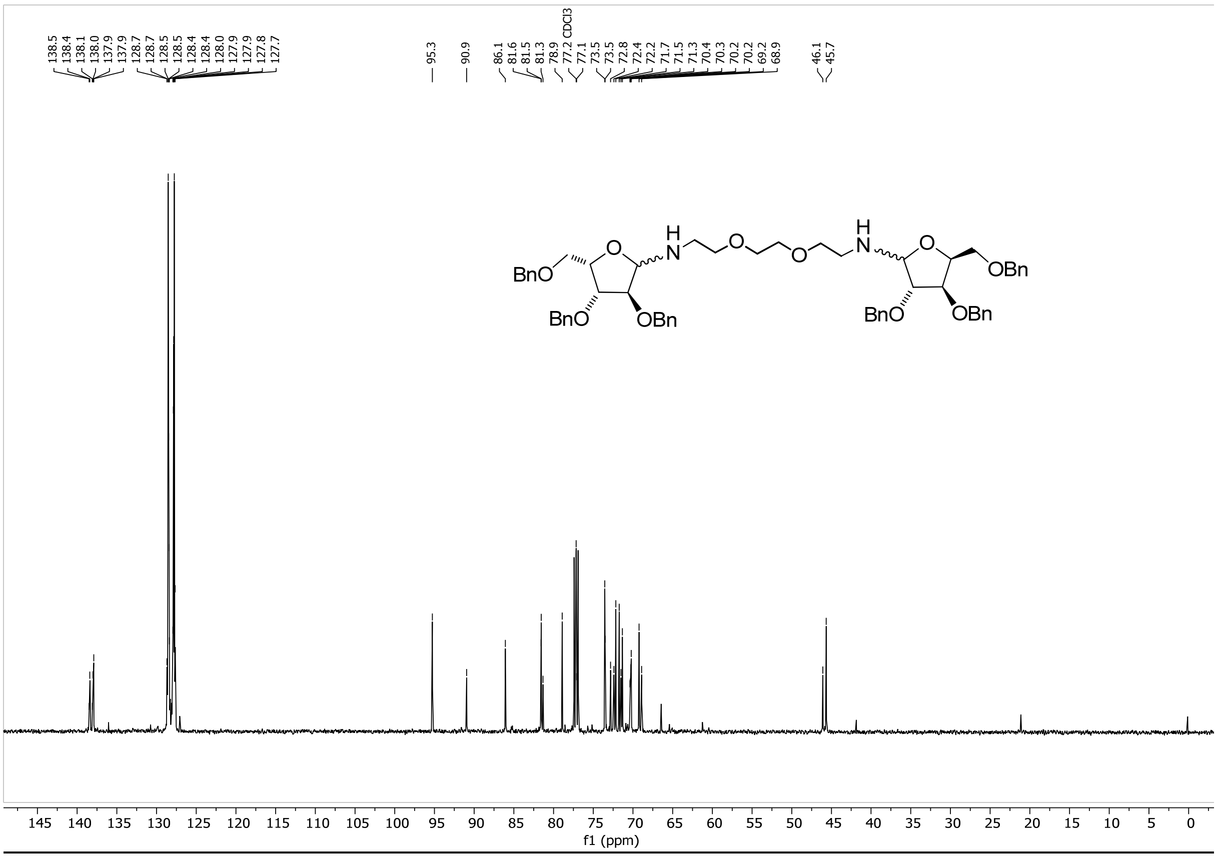

^{1}H-NMR spectrum of 10e ($CDCl_3$)

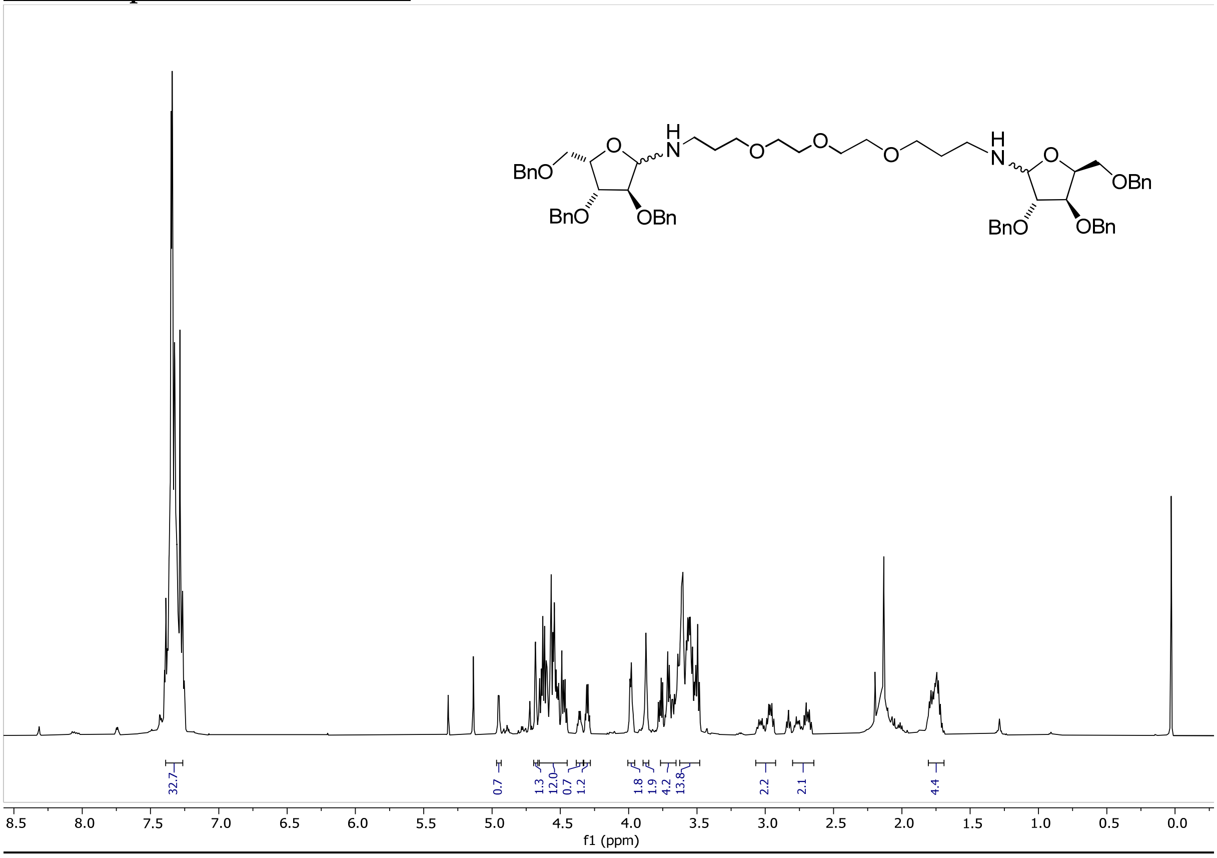

^{13}C-NMR spectrum of 10e ($CDCl_3$)

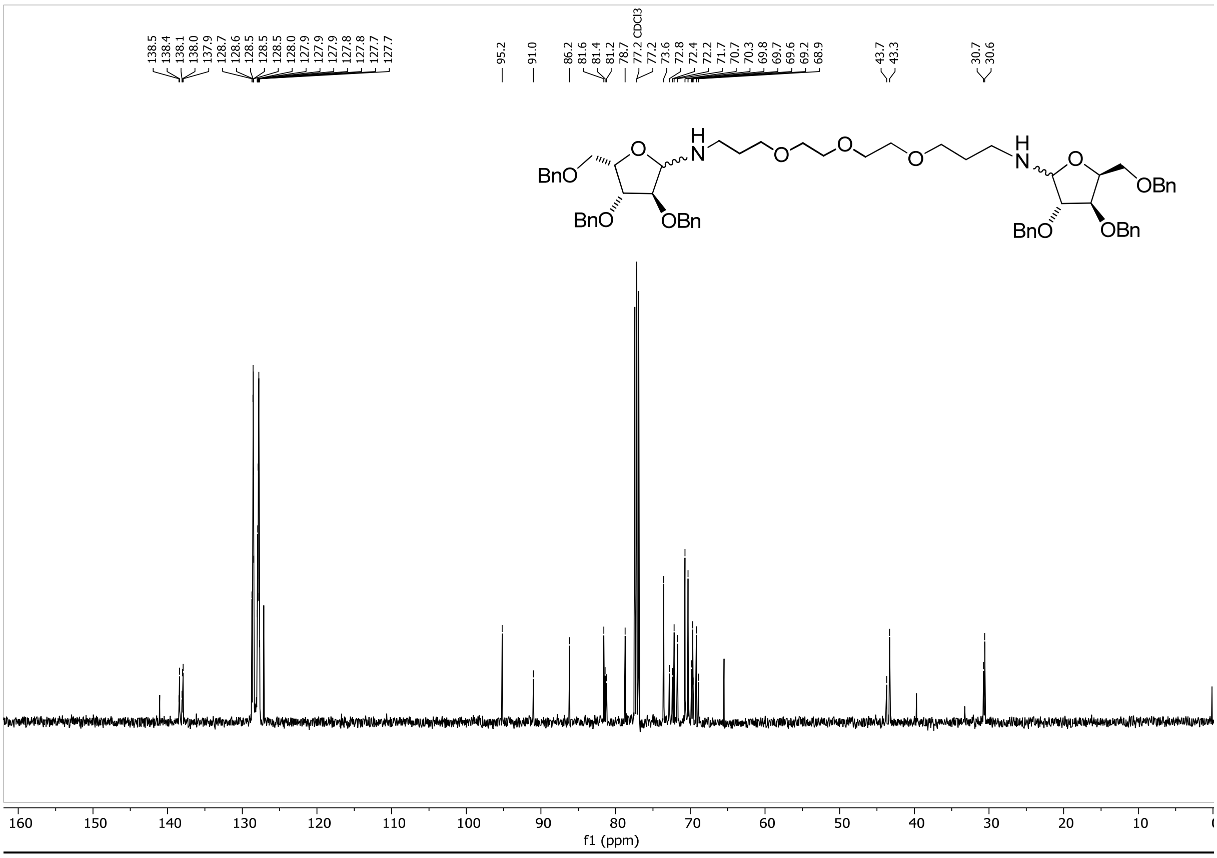

^{1}H-NMR spectrum of 11a ($CDCl_3$)

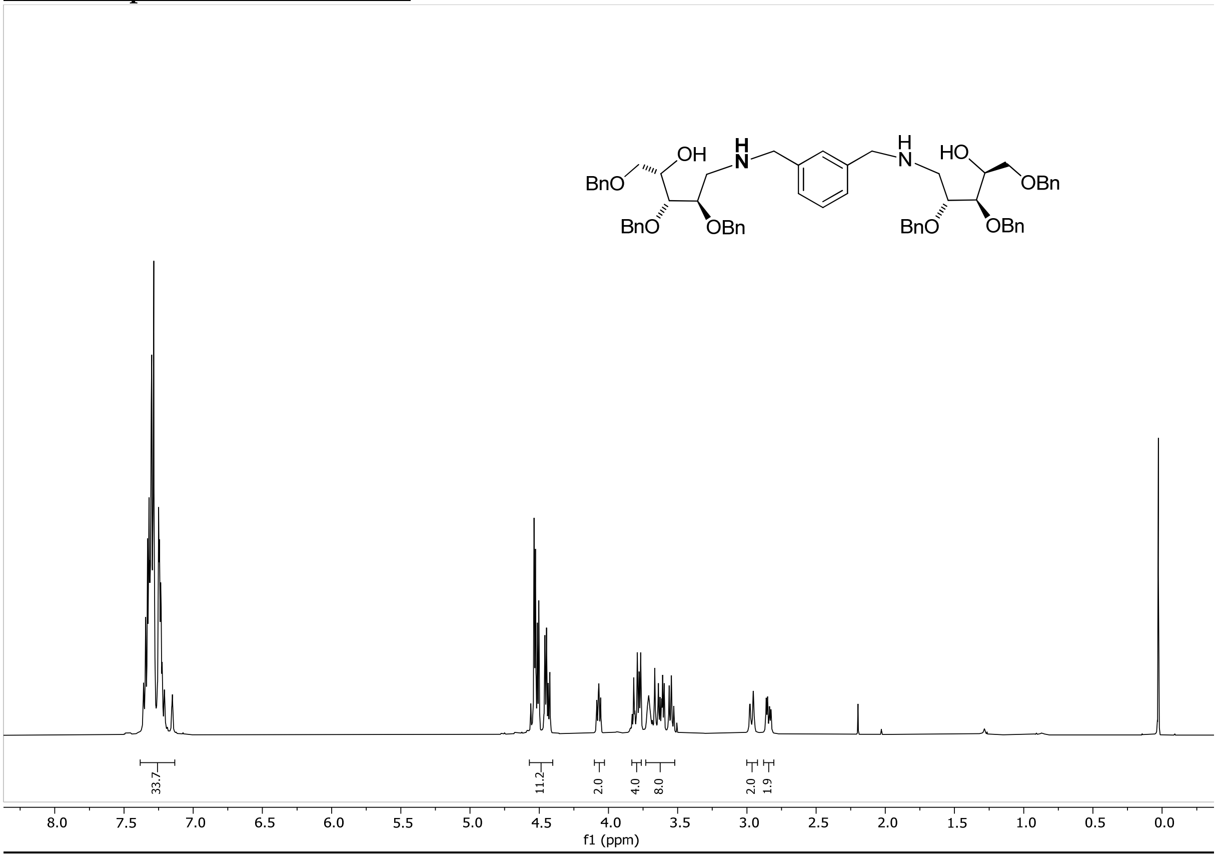

^{13}C-NMR spectrum of 11a ($CDCl_3$)

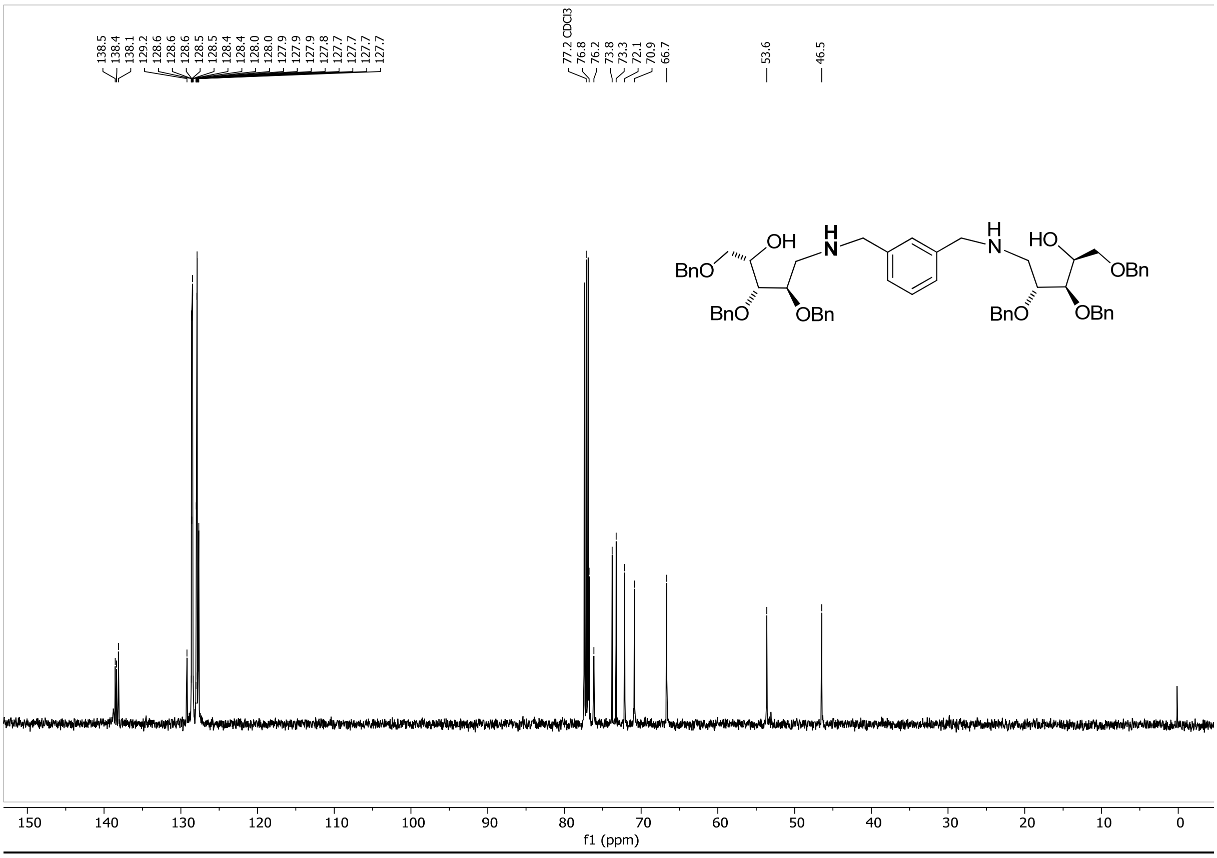

^{1}H-NMR spectrum of 11b ($CDCl_3$)

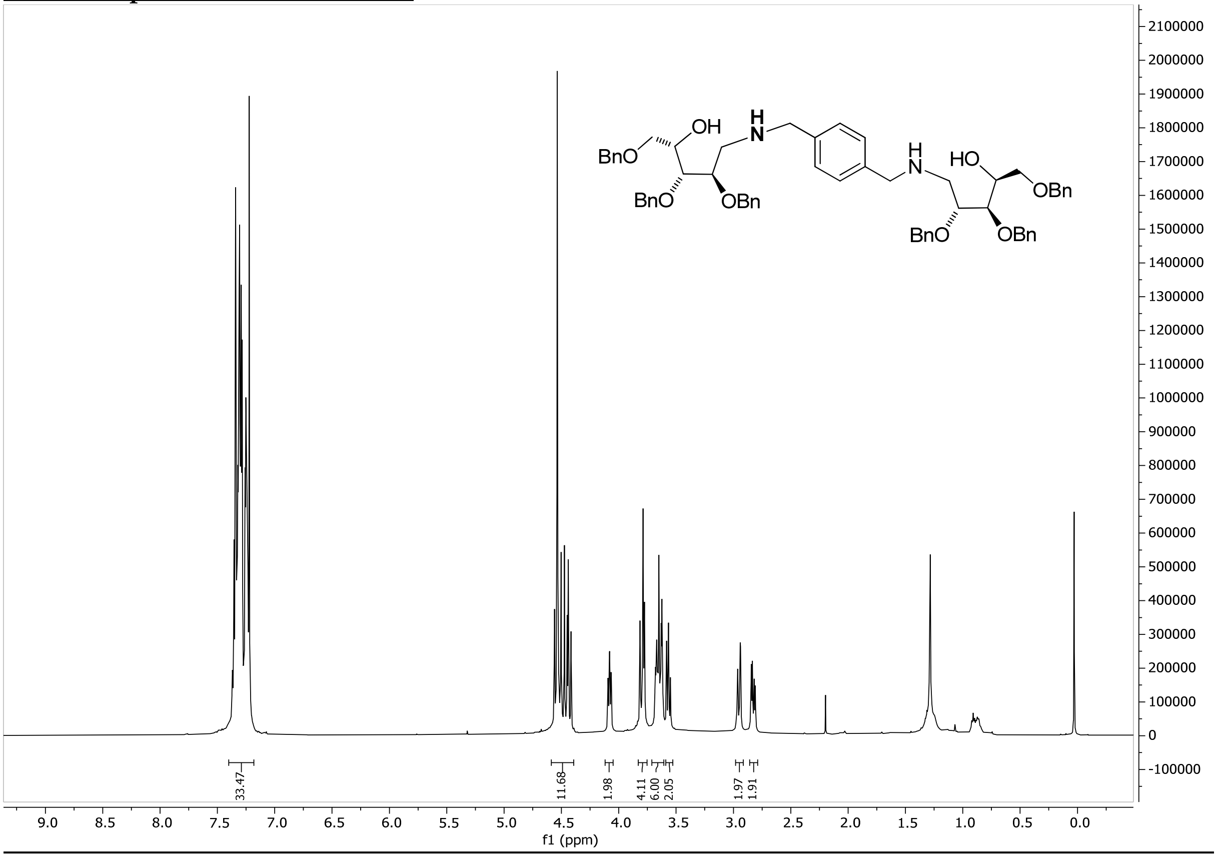

^{13}C-NMR spectrum of 11b ($CDCl_3$)

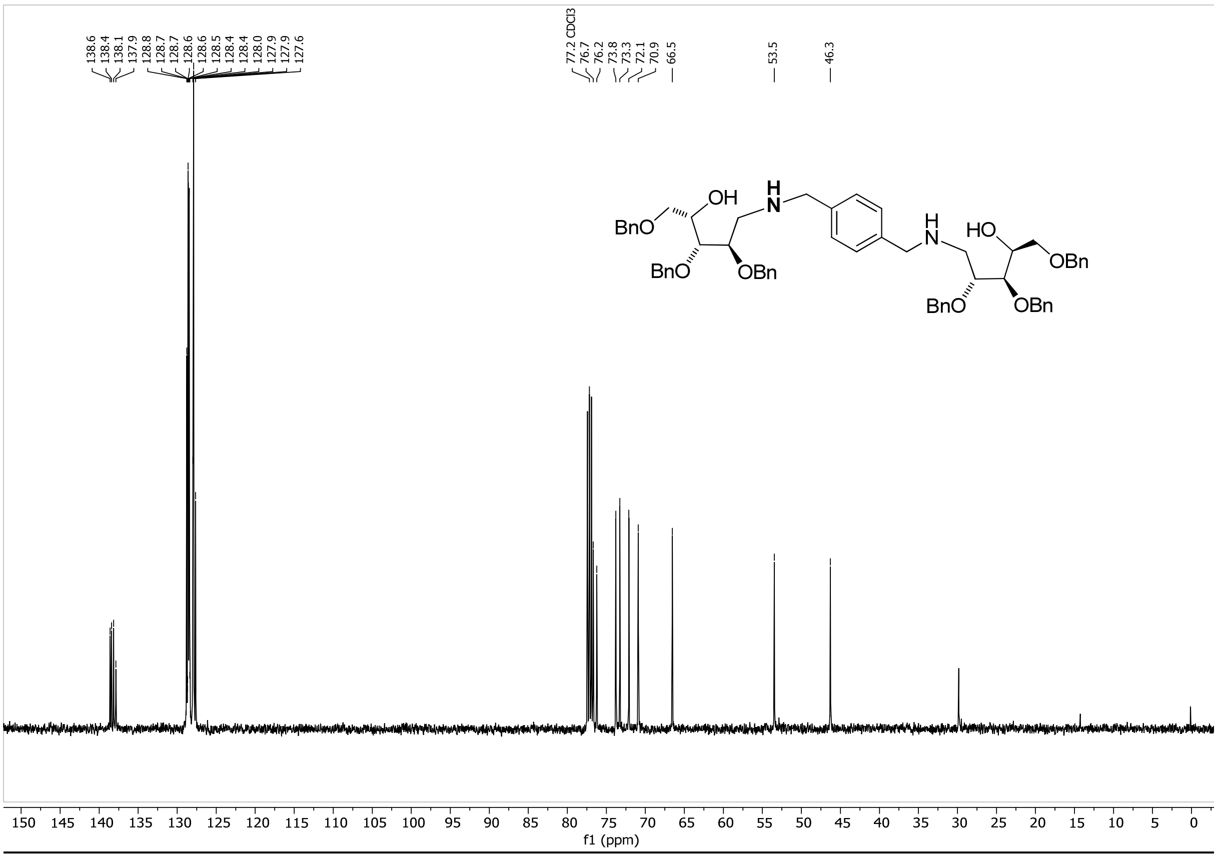

^{1}H-NMR spectrum of 11c (CDCl3)

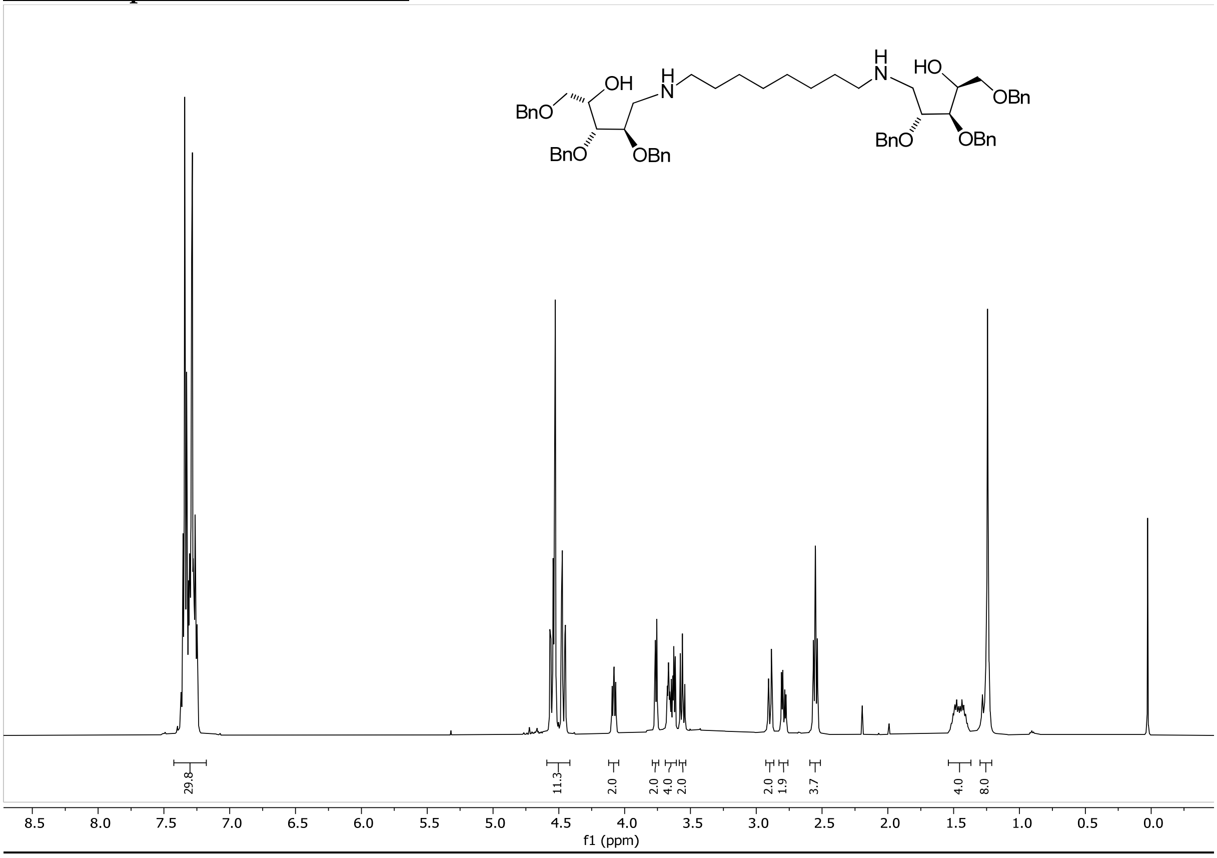

^{13}C-NMR spectrum of 11c (CDCl3)

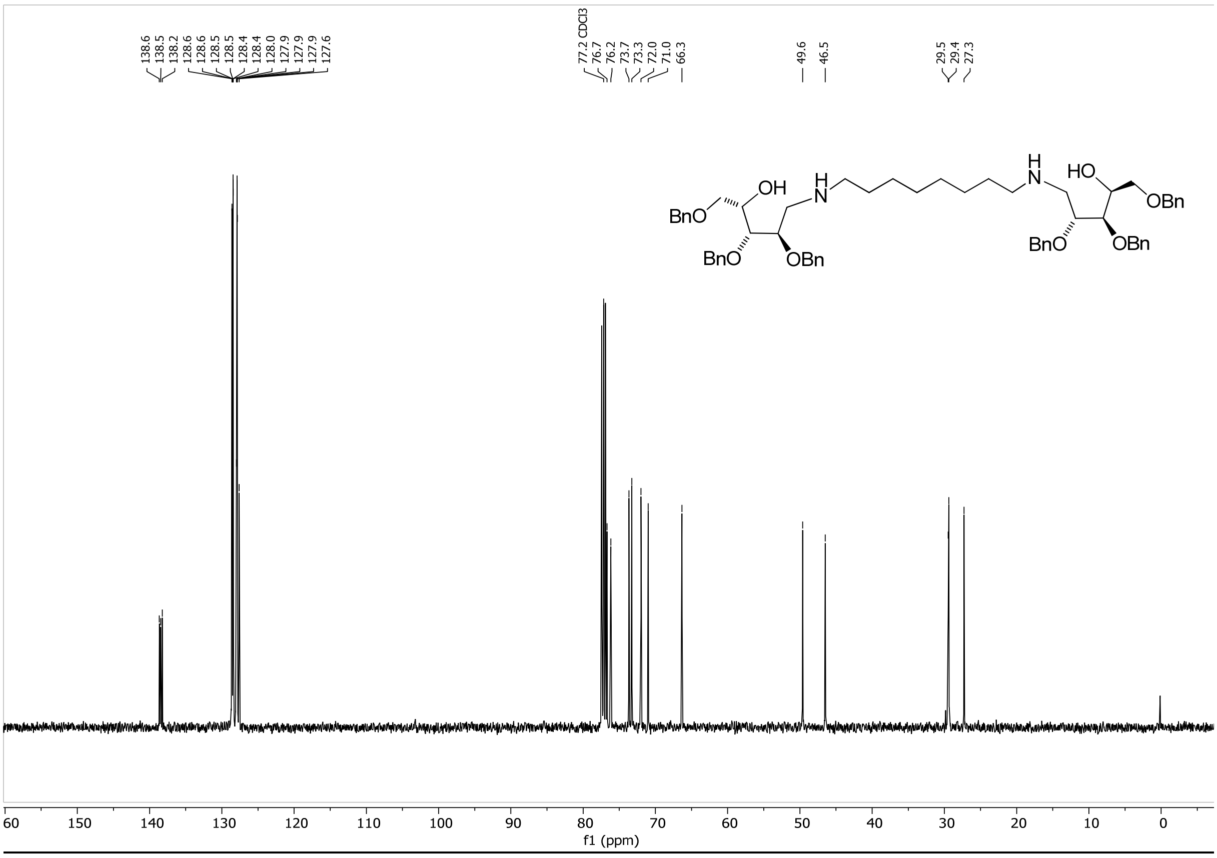

^{1}H-NMR spectrum of 11d (CDCl$_3$)

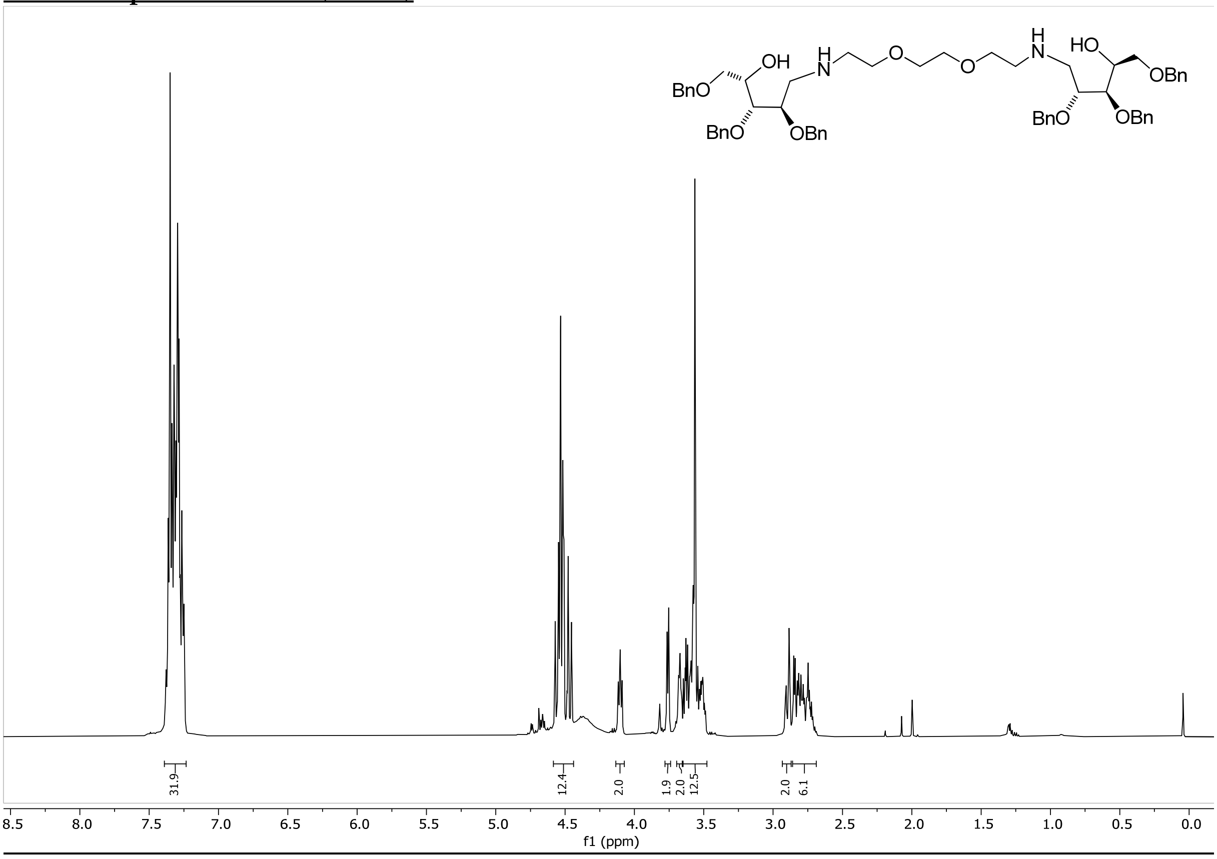

^{13}C-NMR spectrum of 11d (CDCl$_3$)

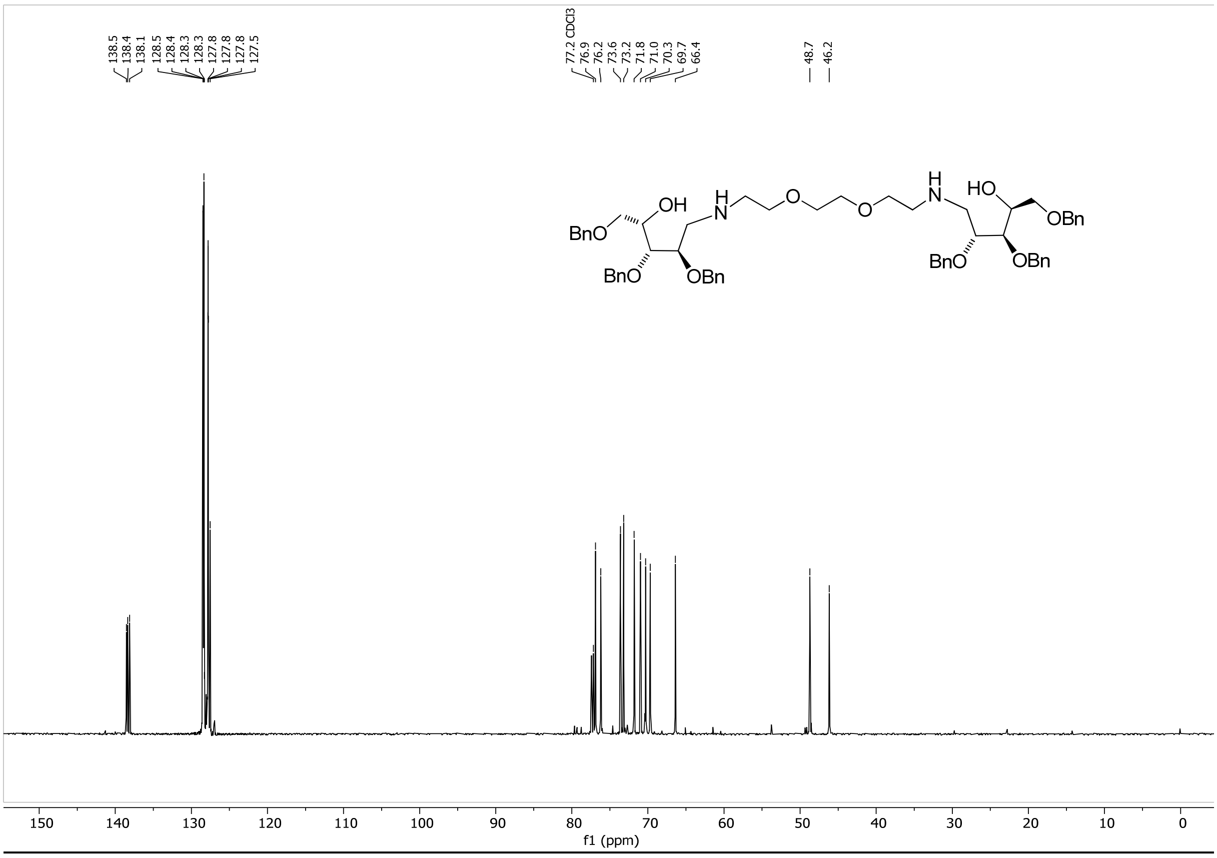

^{1}H-NMR spectrum of 11e ($CDCl_3$)

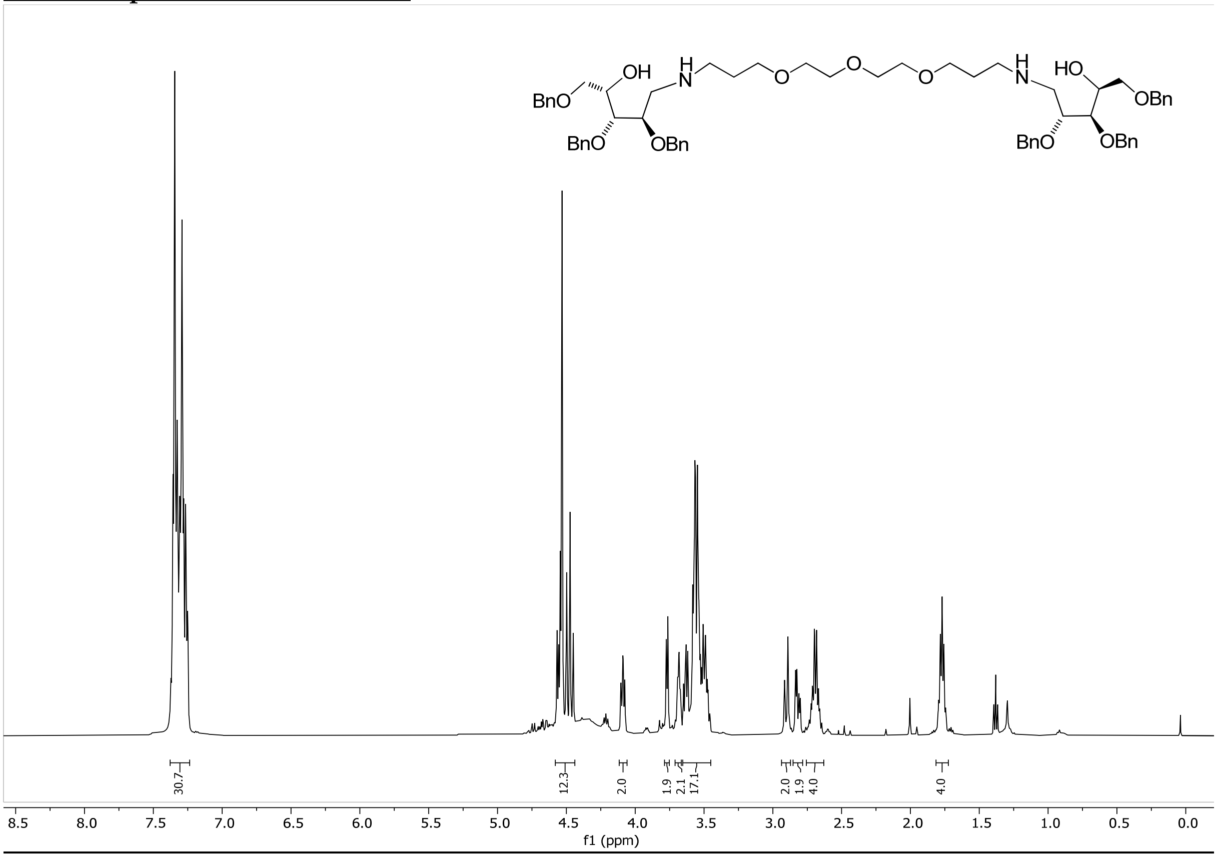

^{13}C-NMR spectrum of 11e ($CDCl_3$)

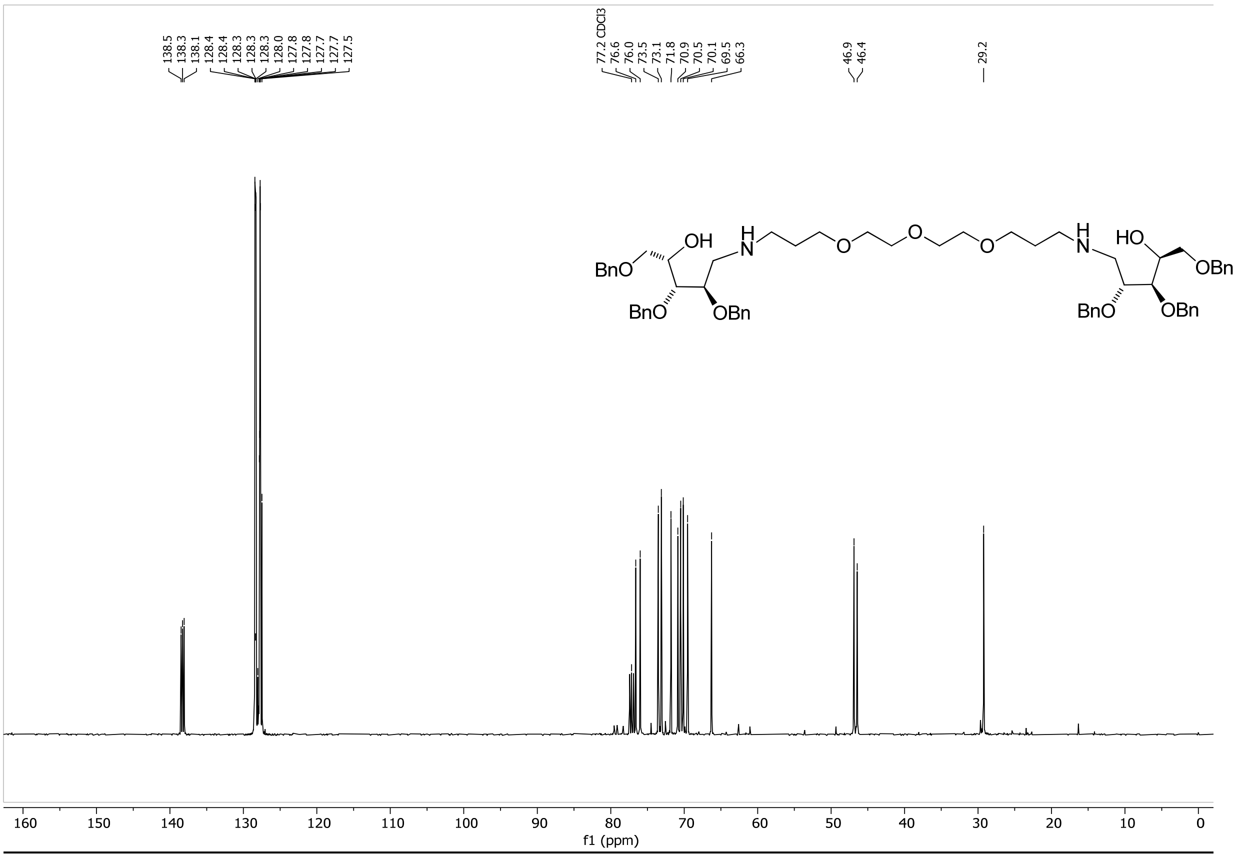

<u>^{1}H-NMR spectrum of 12a (CDCl3)</u>

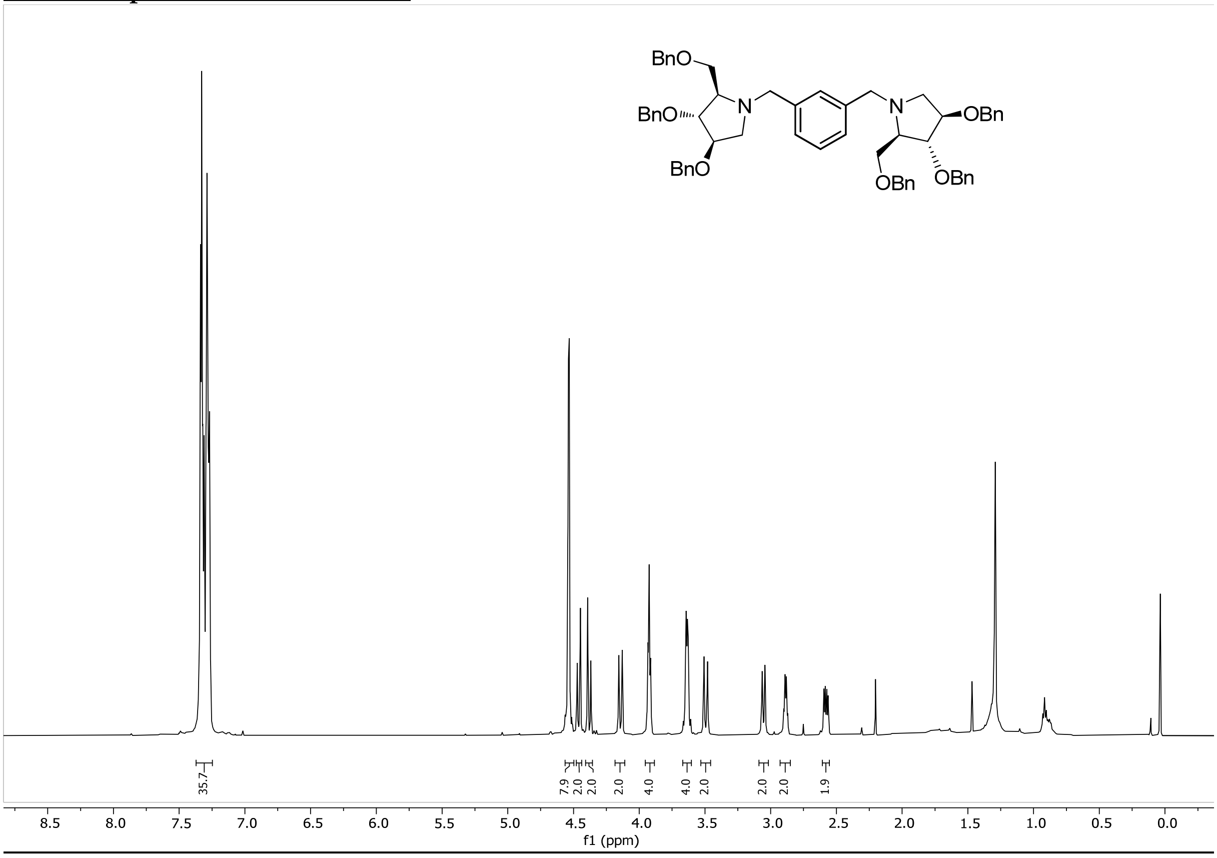

<u>^{13}C-NMR spectrum of 12a (CDCl3)</u>

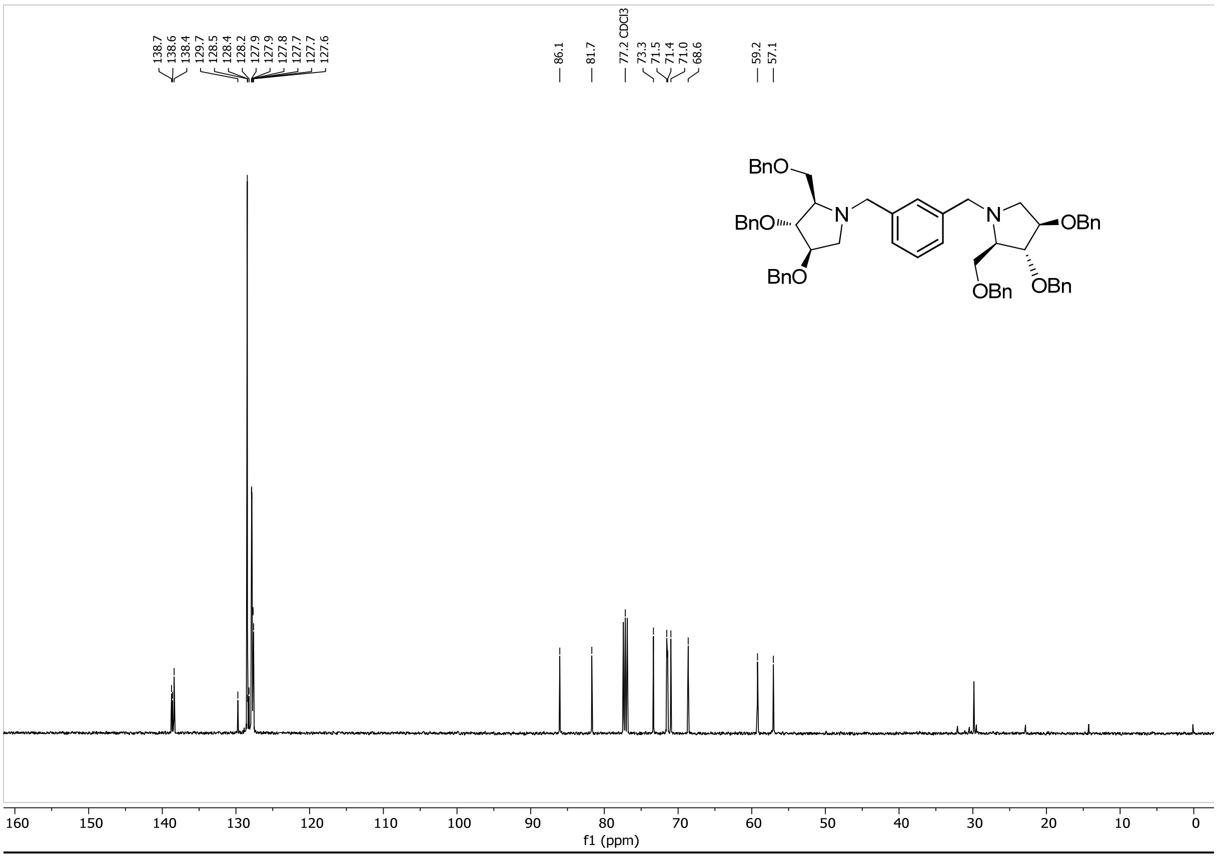

<u>^{1}H-NMR spectrum of 12b (CDCl3)</u>

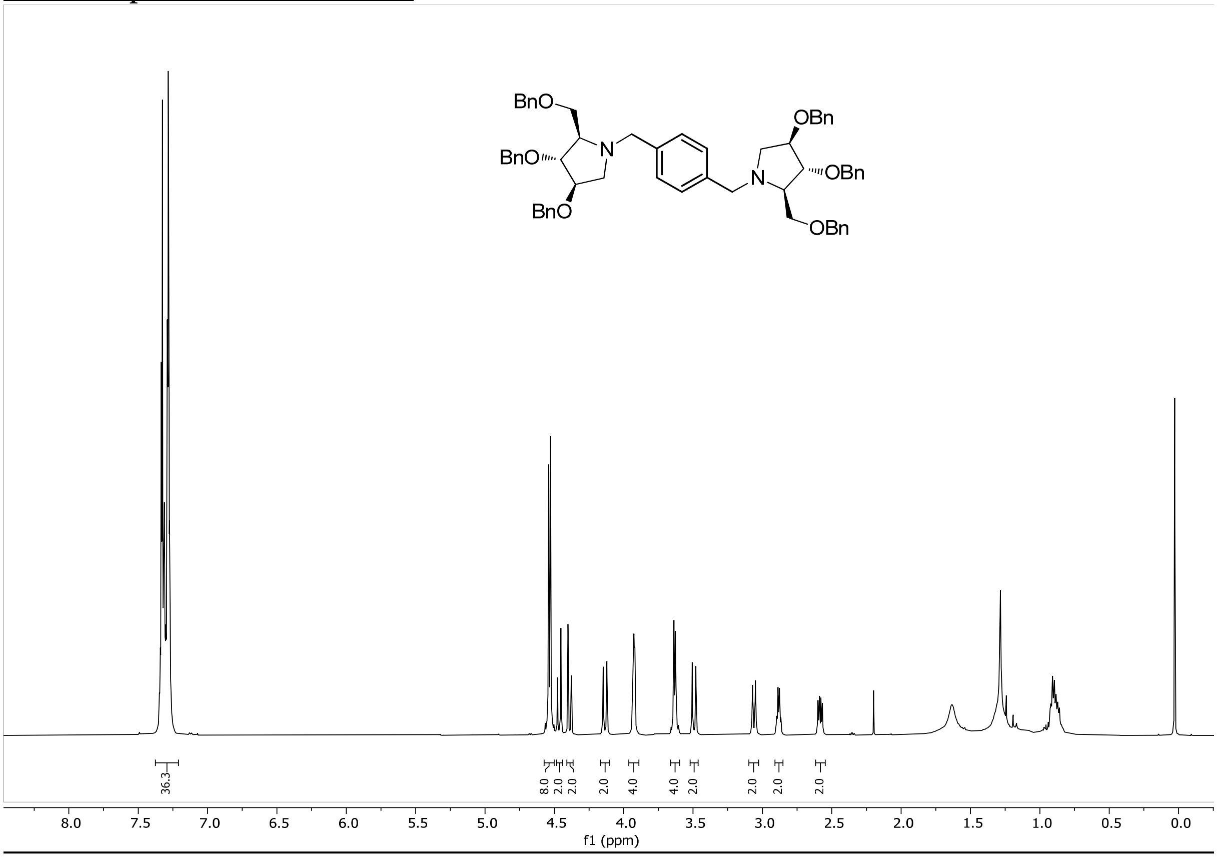

<u>^{13}C-NMR spectrum of 12b (CDCl3)</u>

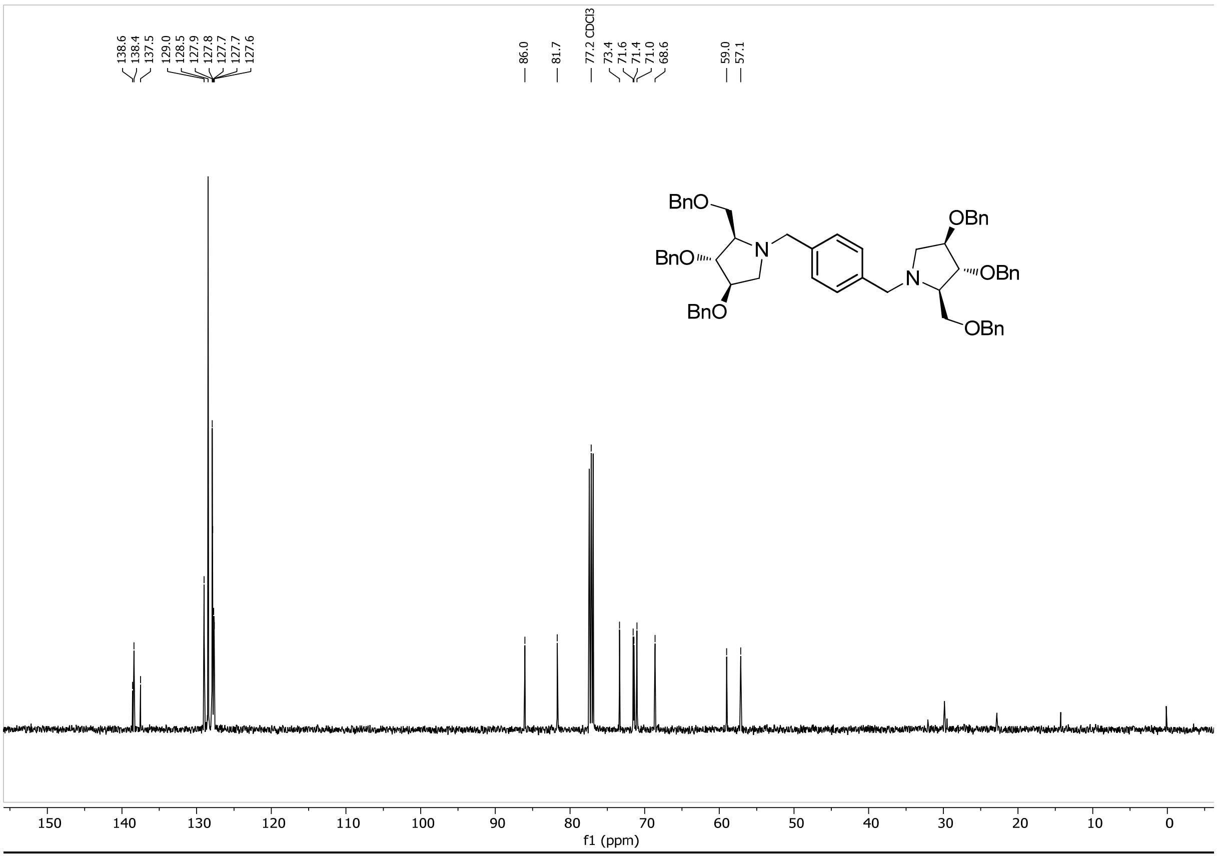

^{1}H-NMR spectrum of 12c ($CDCl_3$)

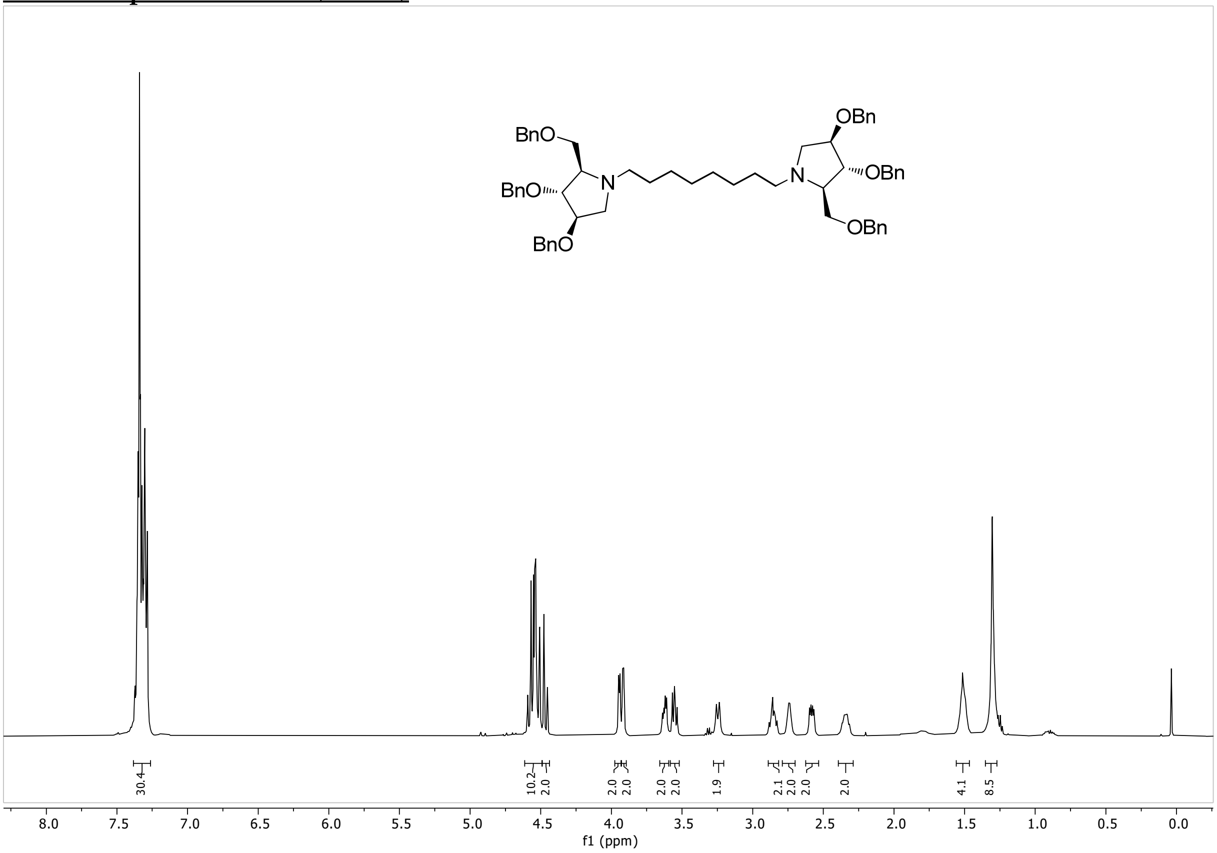

^{13}C-NMR spectrum of 12c ($CDCl_3$)

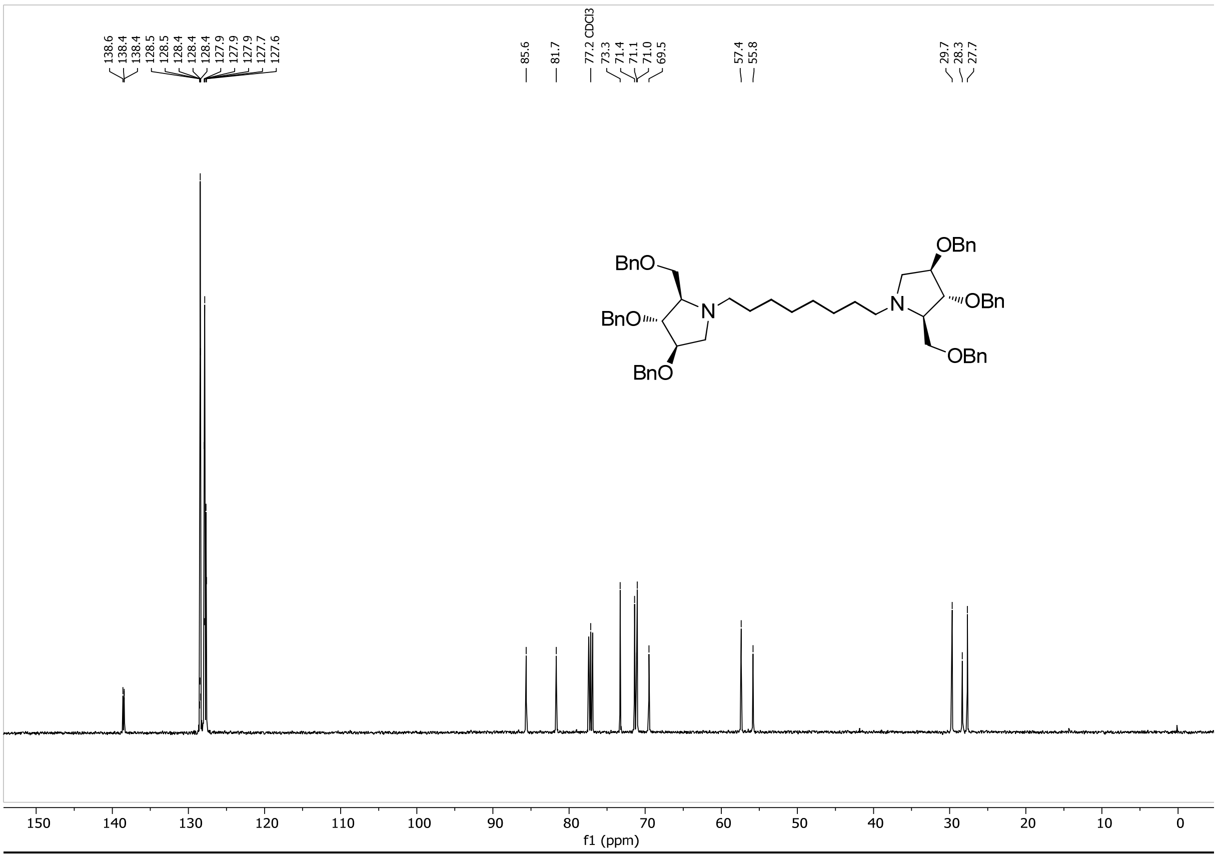

^{1}H-NMR spectrum of 12d ($CDCl_3$)

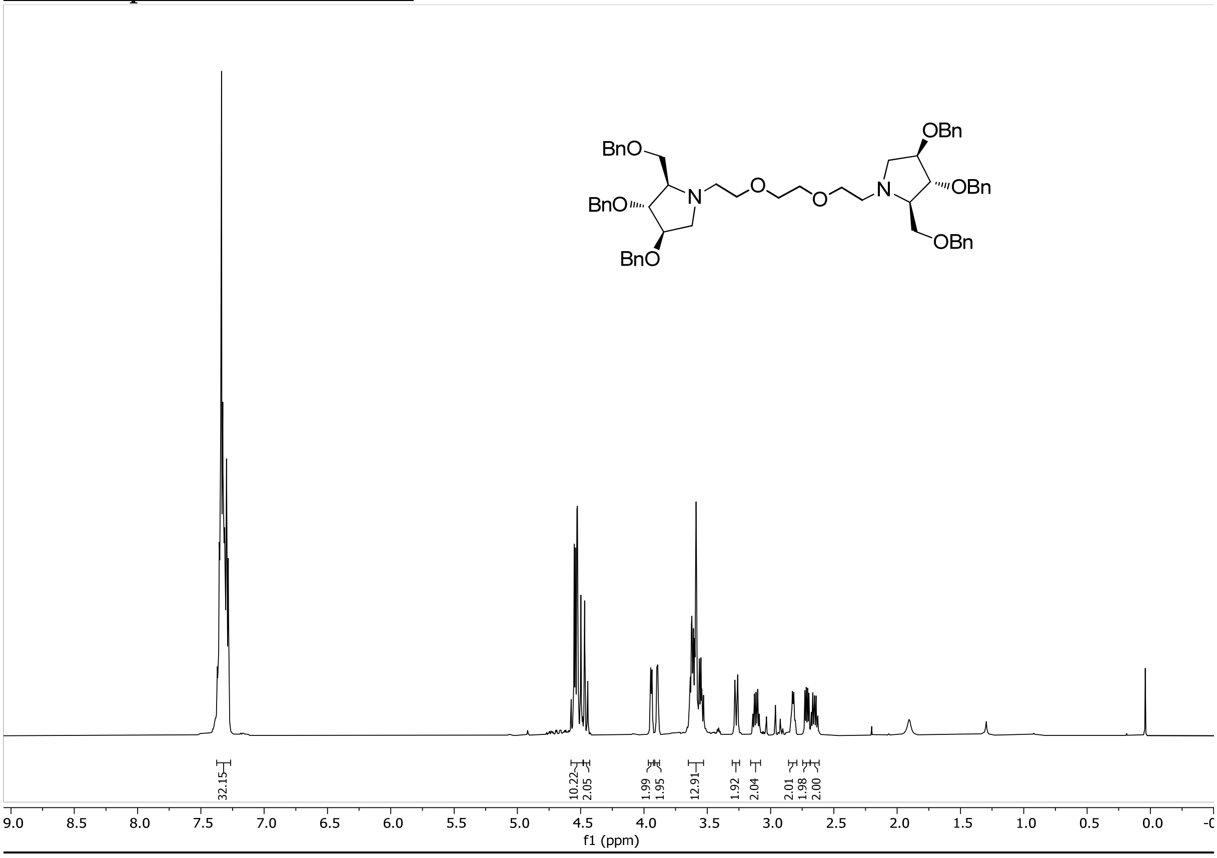

^{13}C-NMR spectrum of 12d ($CDCl_3$)

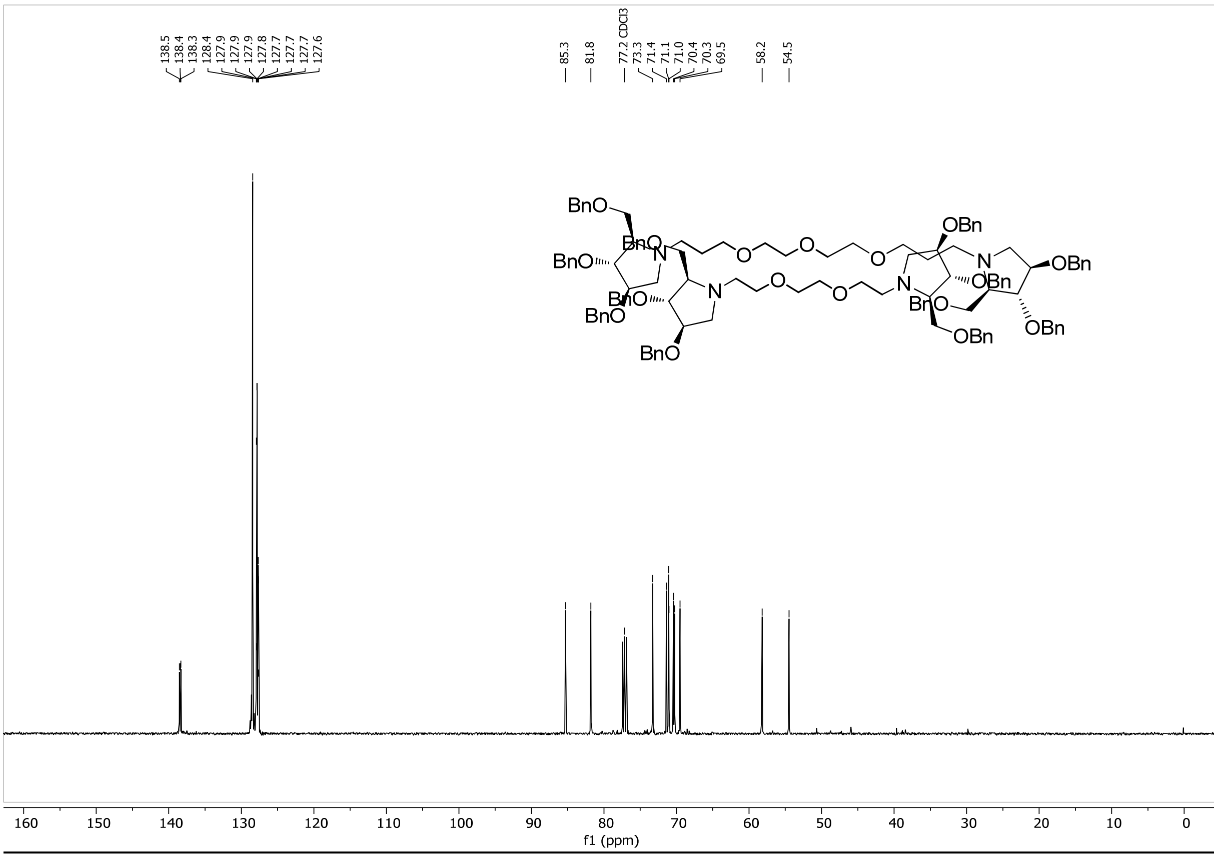

¹H-NMR spectrum of 12e (CDCl₃)

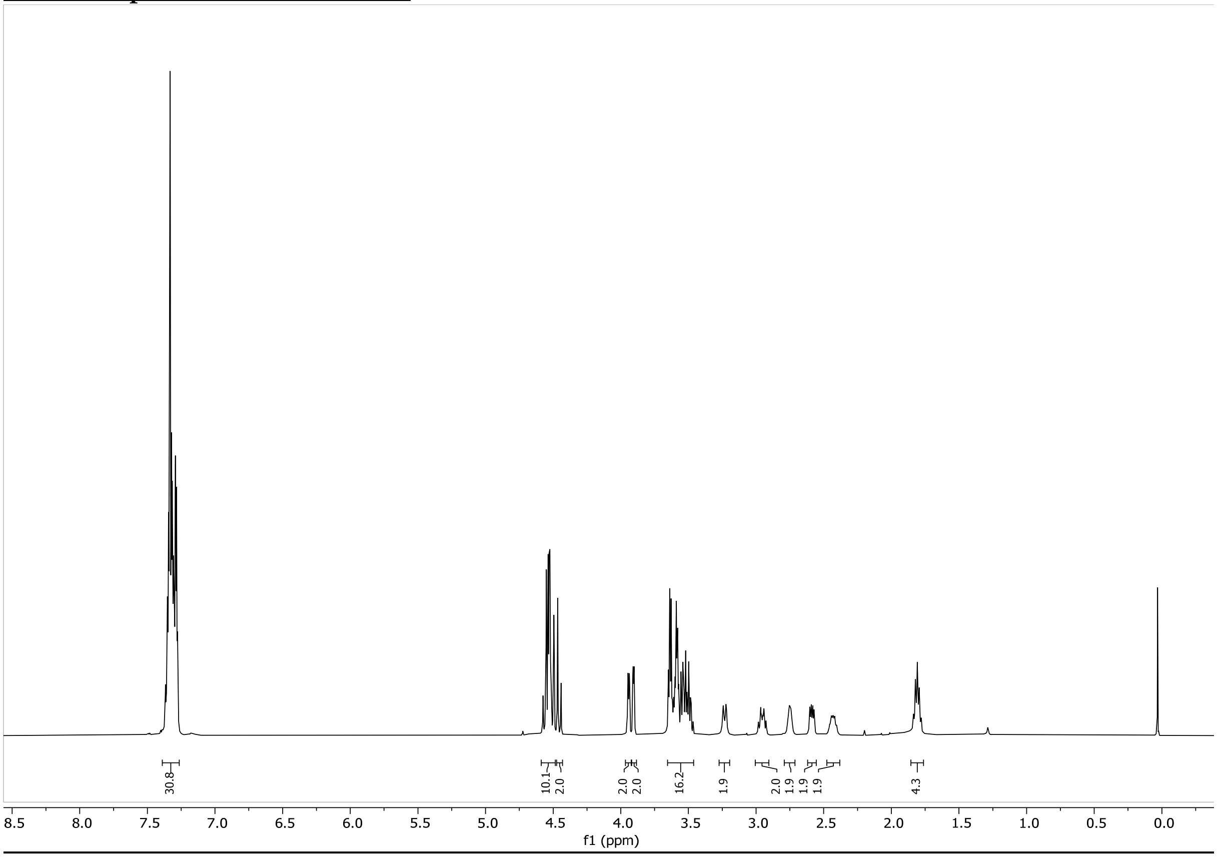

¹³C-NMR spectrum of 12e (CDCl₃)

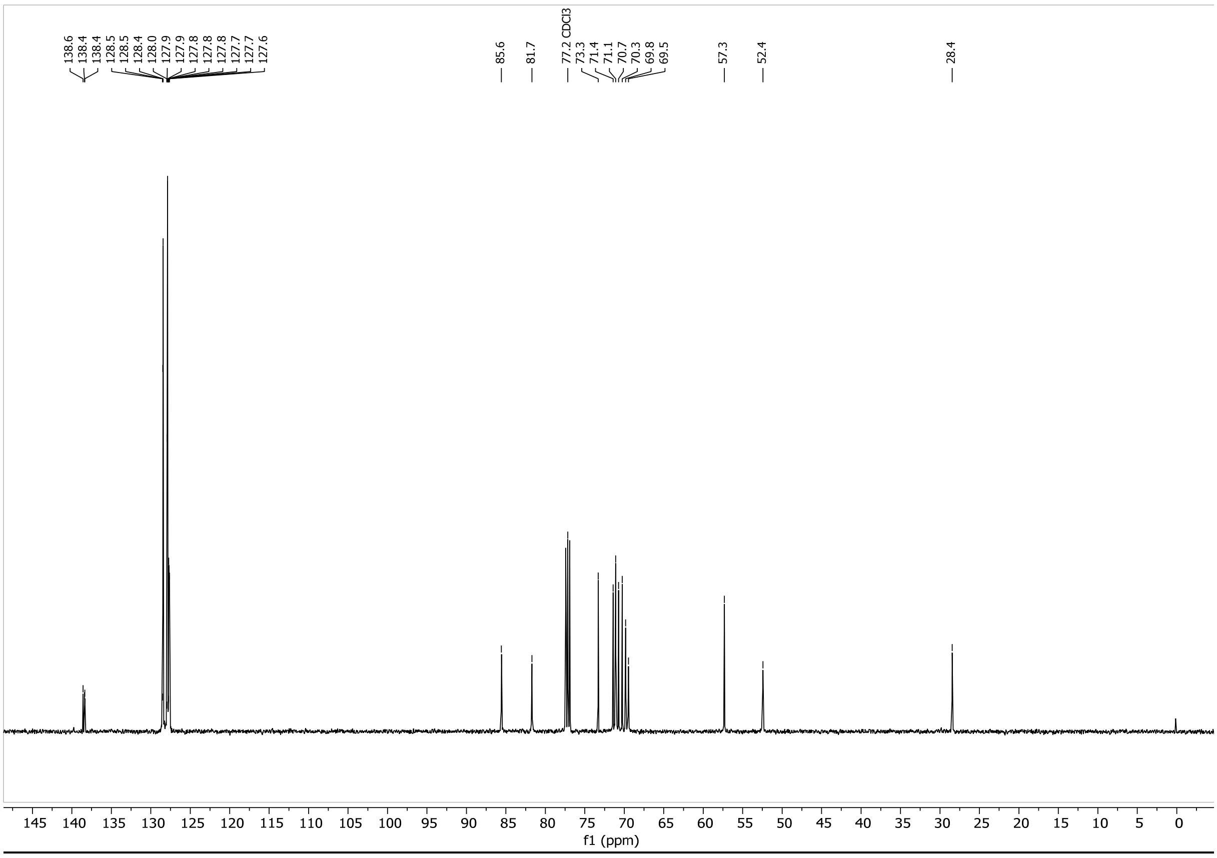

<u>^{1}H-NMR spectrum of 13a (D_2O)</u>

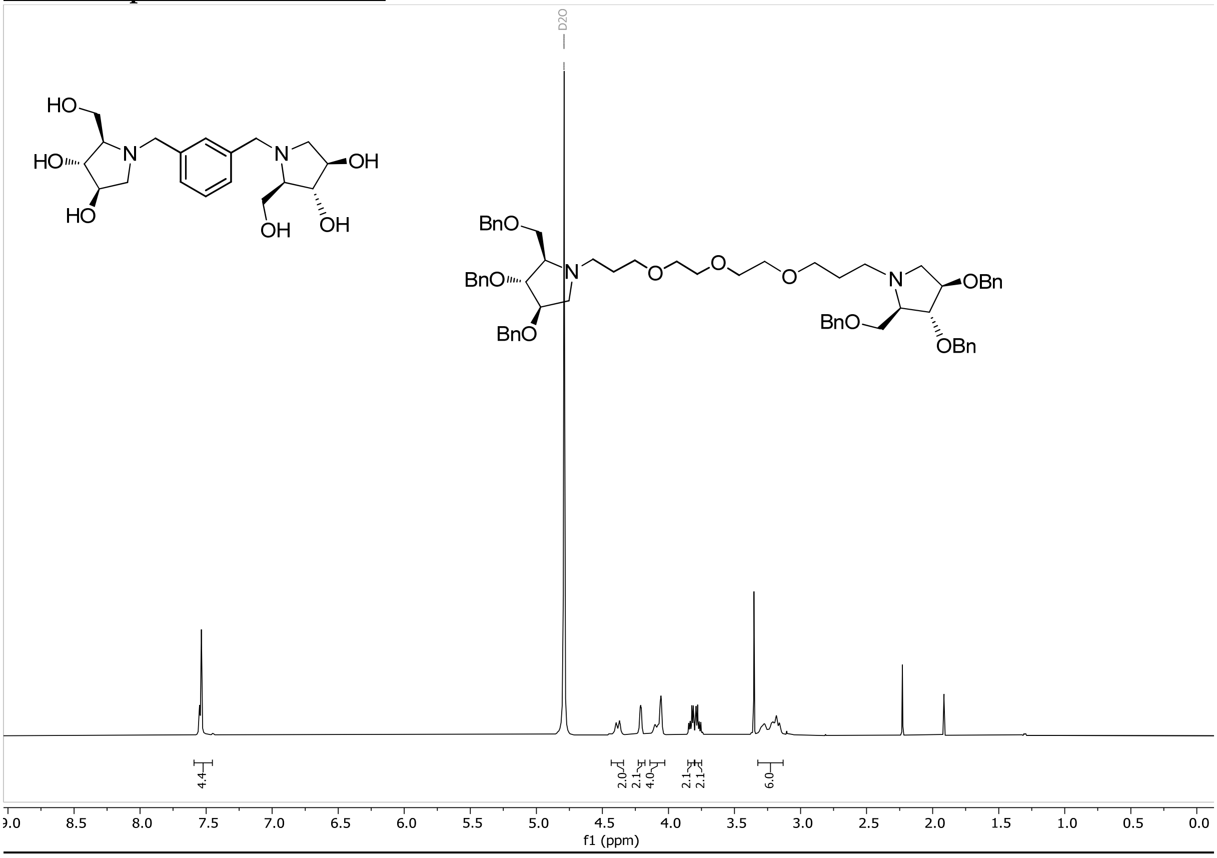

<u>^{13}C-NMR spectrum of 13a (D_2O)</u>

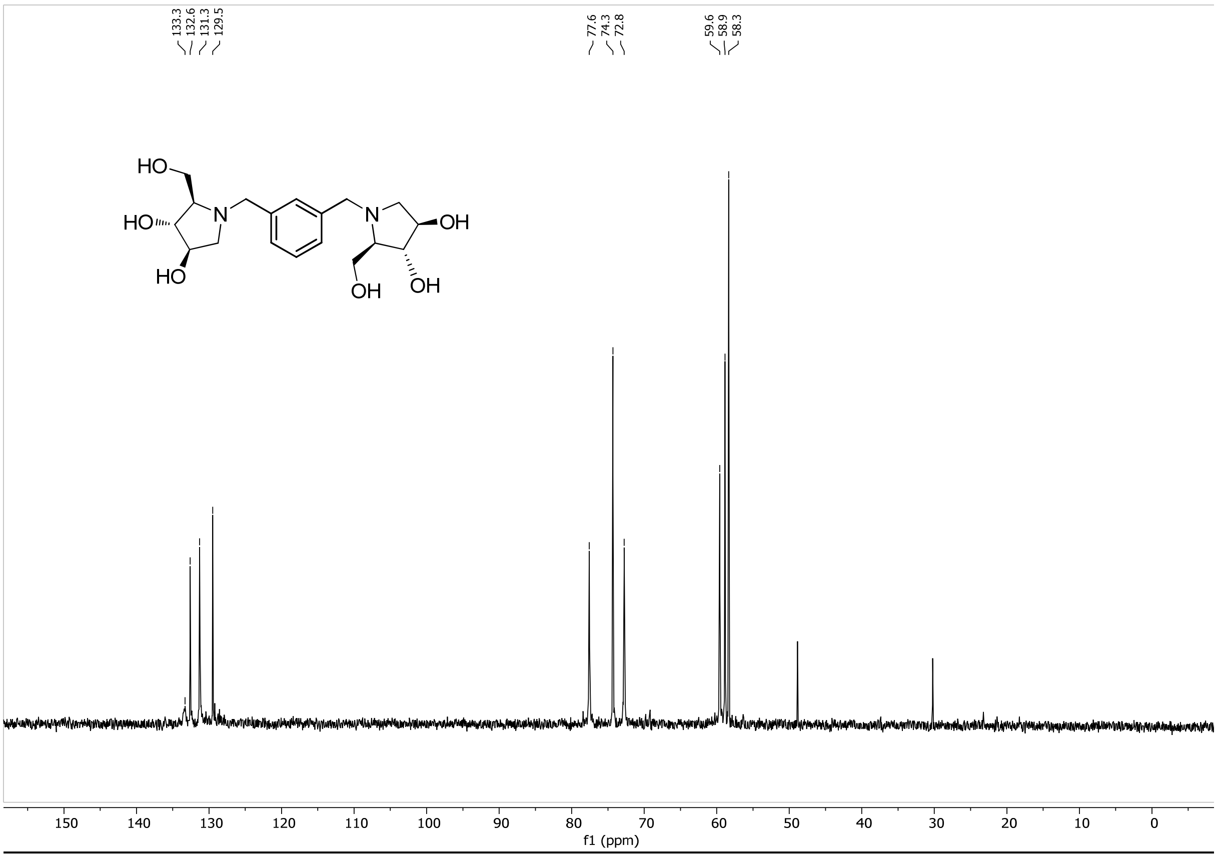

^{1}H-NMR spectrum of 13b (D_2O)

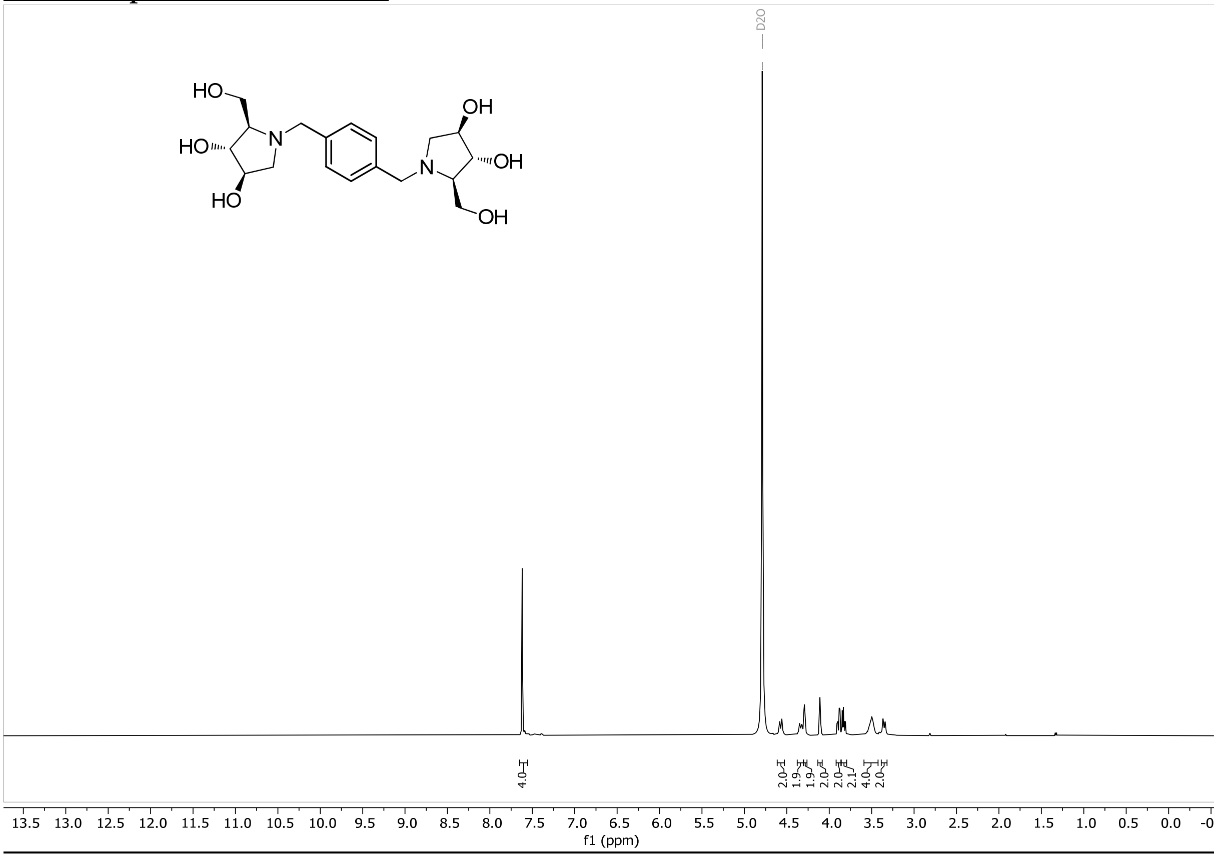

^{13}C-NMR spectrum of 13b (D_2O)

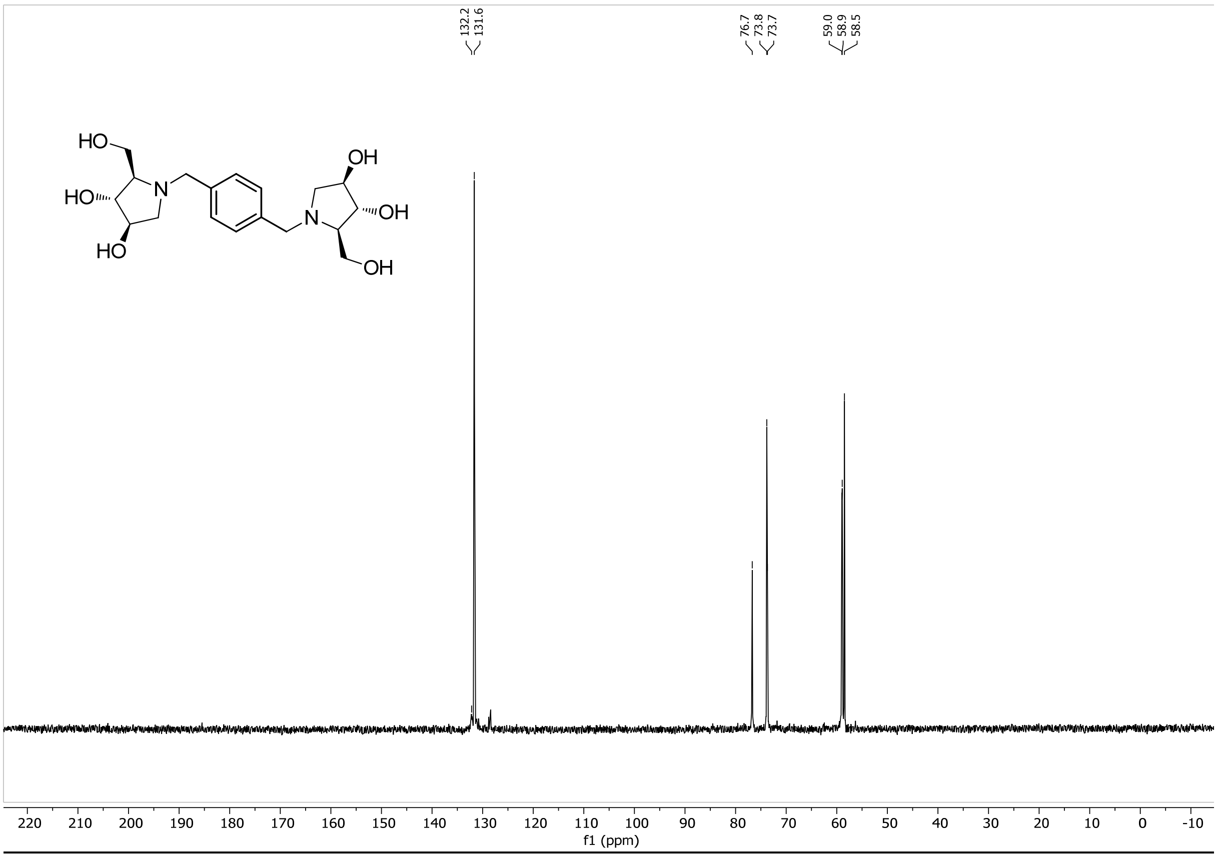

^{1}H-NMR spectrum of 13c (D_2O)

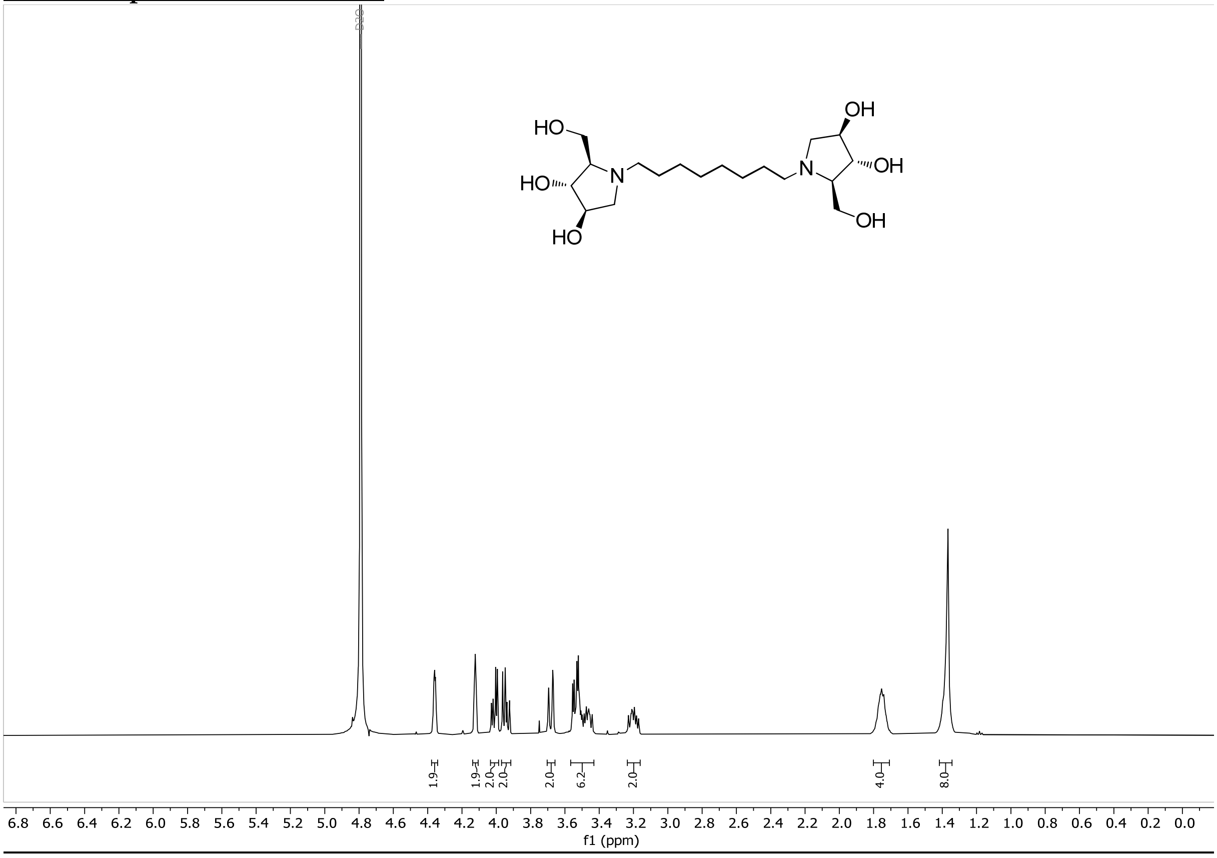

^{13}C-NMR spectrum of 13c (D_2O)

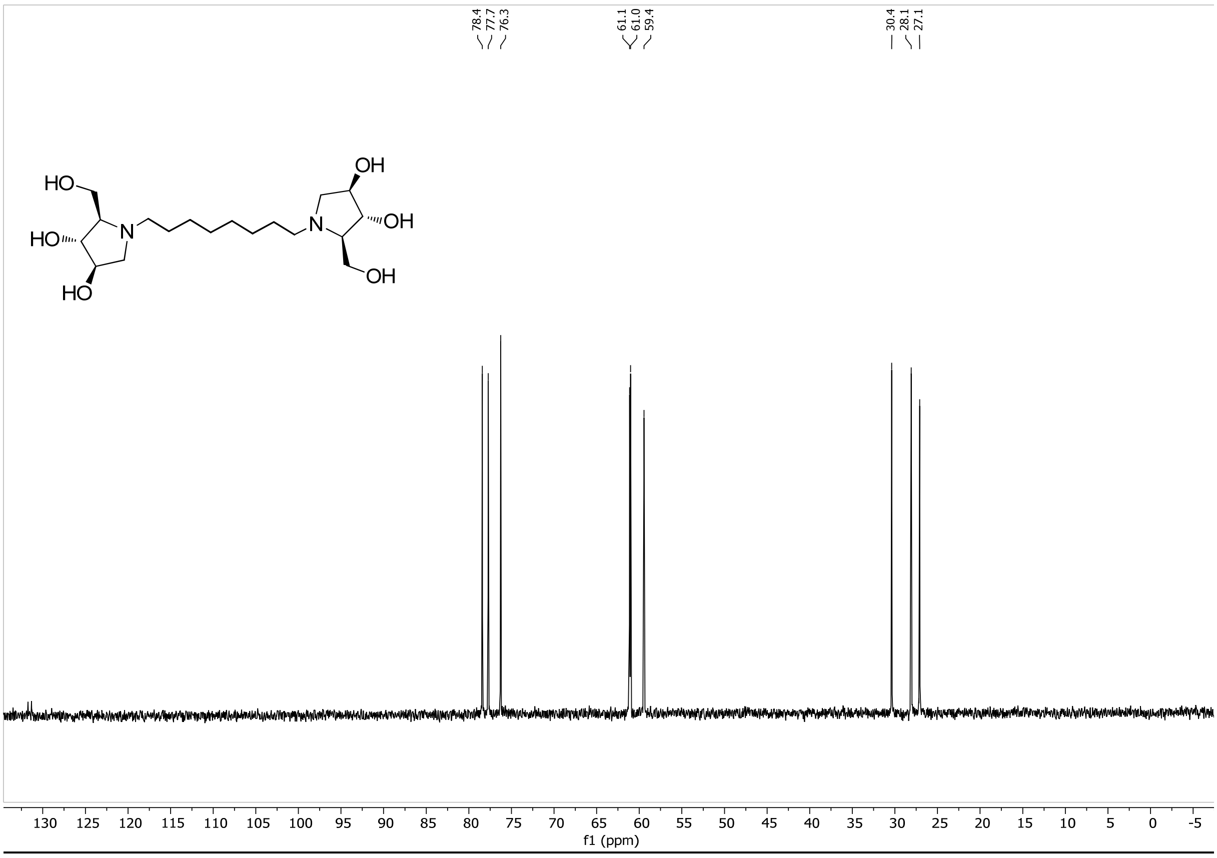

^{1}H-NMR spectrum of 13d (D_2O)

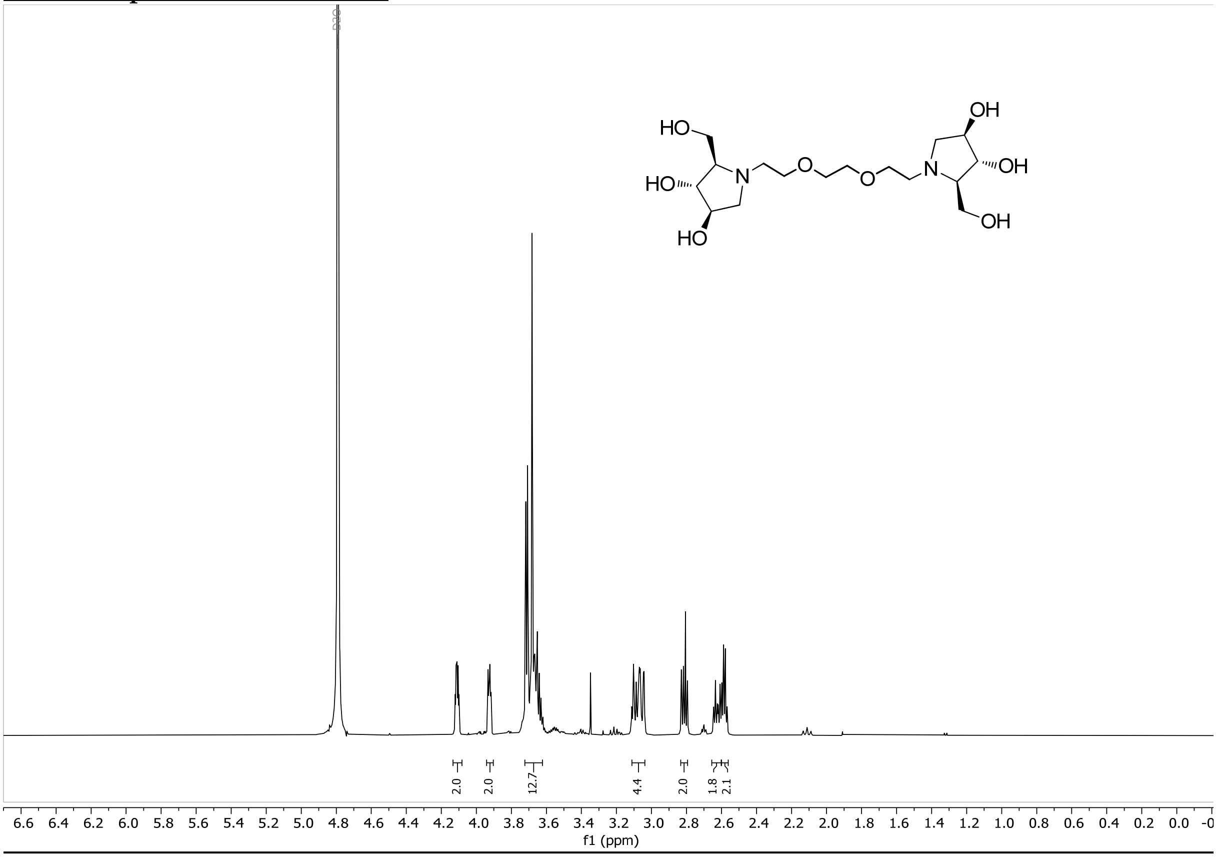

^{13}C-NMR spectrum of 13d (D_2O)

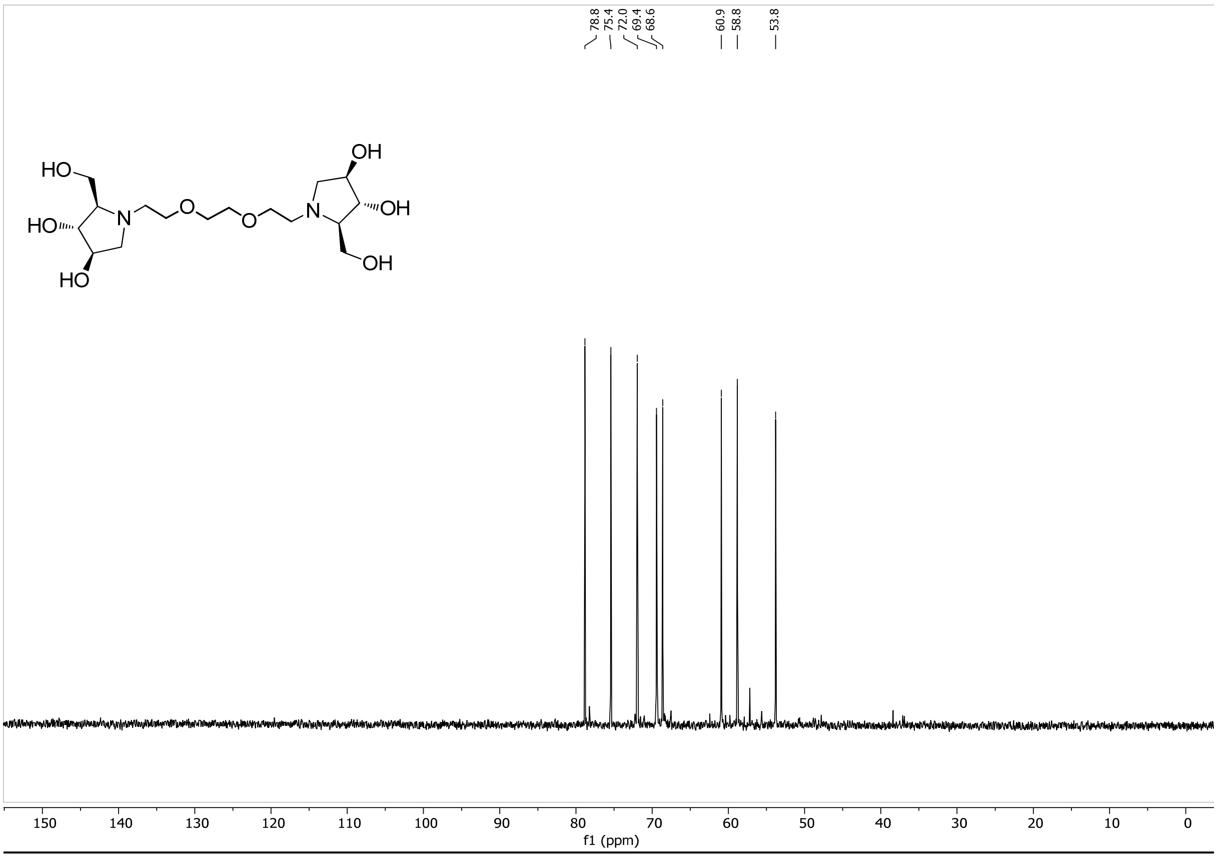

^{1}H-NMR spectrum of 13e (D_2O)

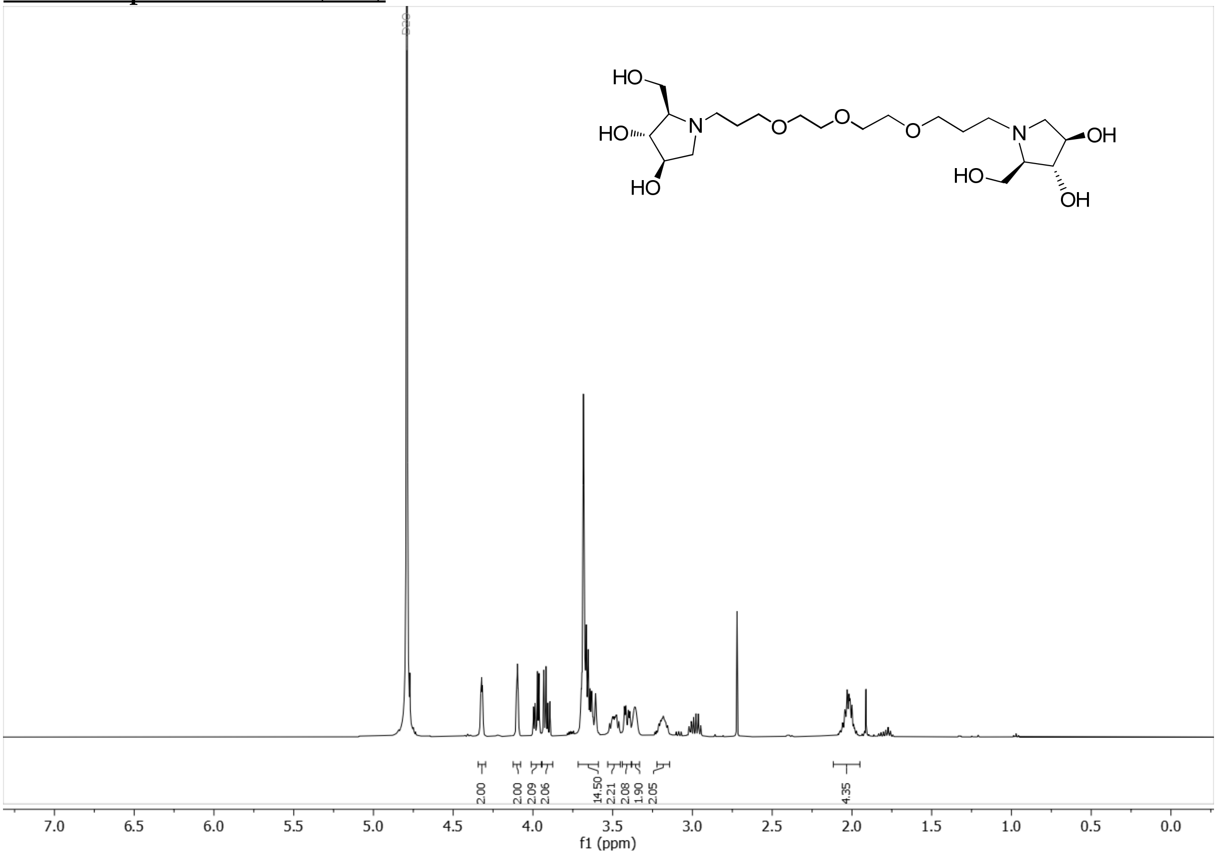

^{13}C-NMR spectrum of 13e (D_2O)

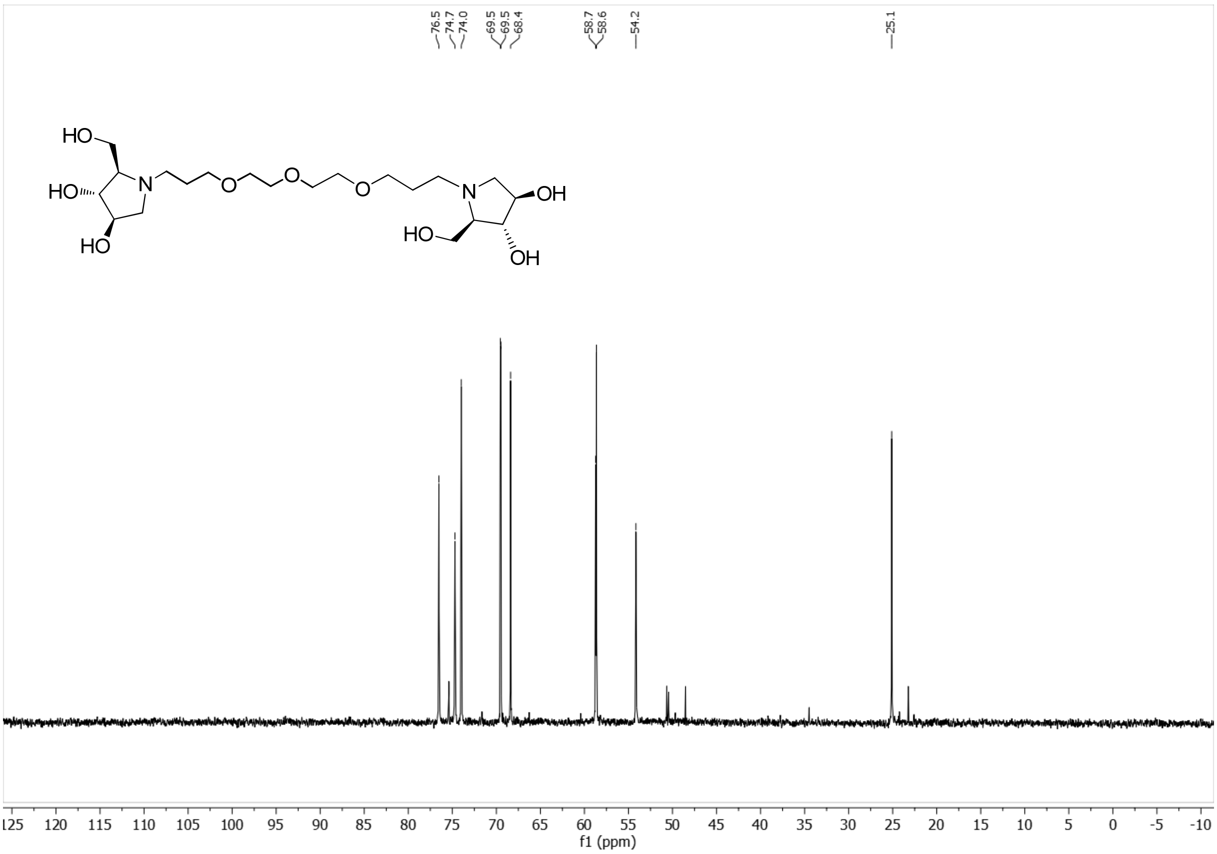

^{1}H-NMR spectrum of 14d ($CDCl_3$)

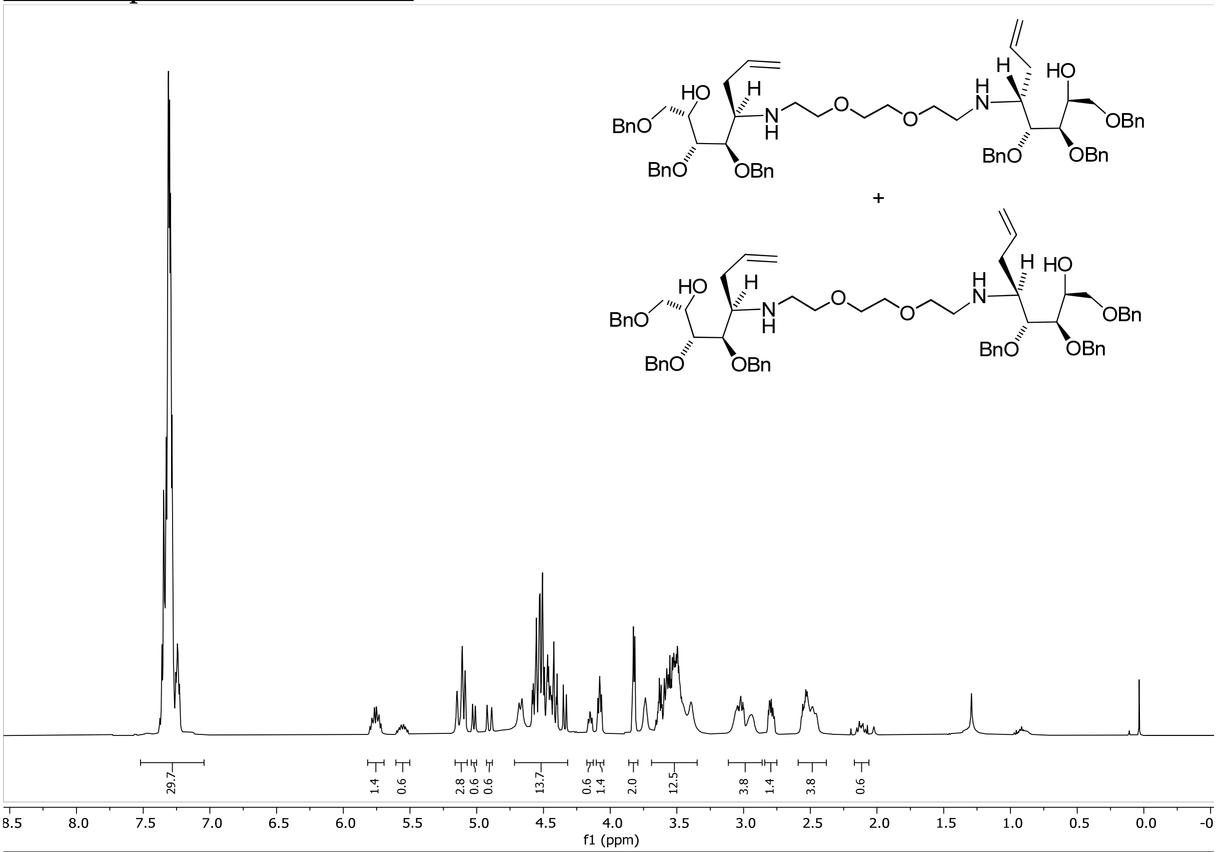

^{13}C-NMR spectrum of 14d ($CDCl_3$)

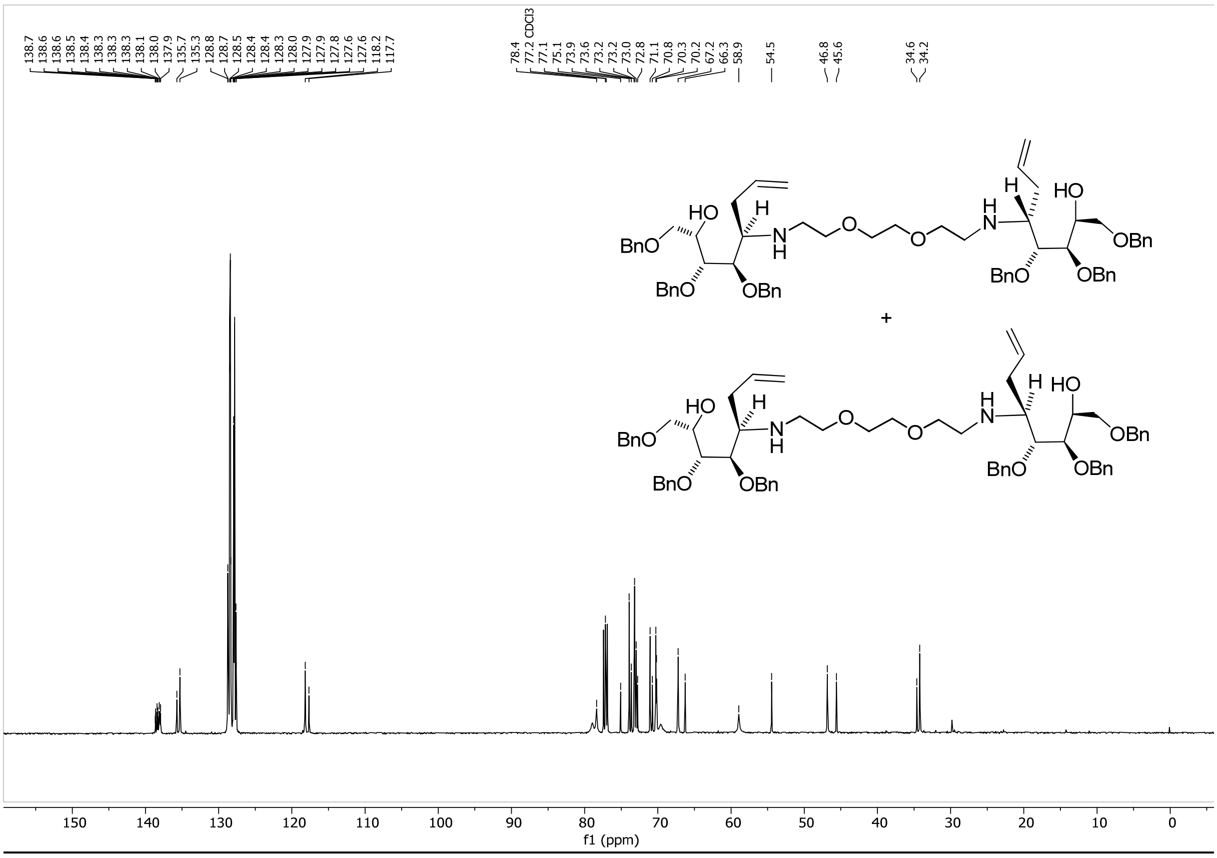

^{1}H-NMR spectrum of 15d(*R*,*R*) (CDCl$_3$)

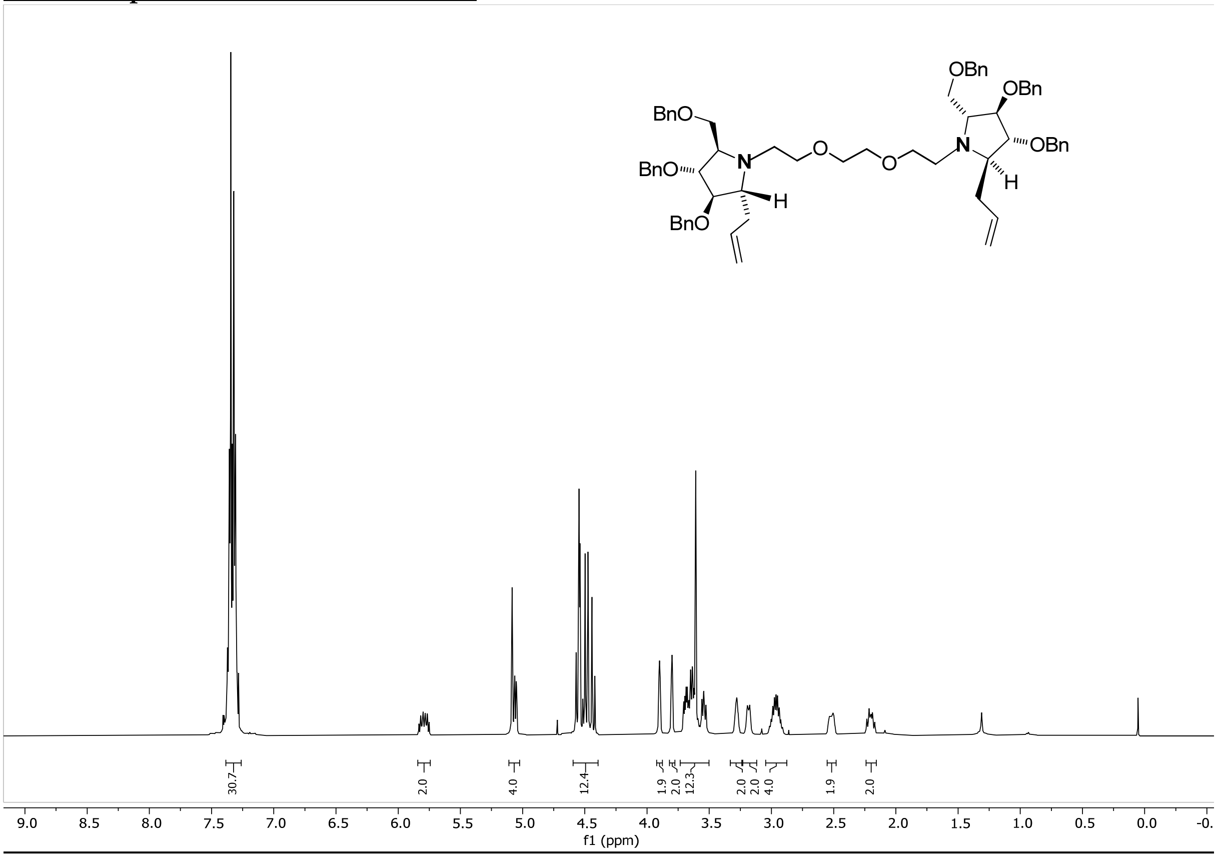

^{13}C-NMR spectrum of 15d(*R*,*R*) (CDCl$_3$)

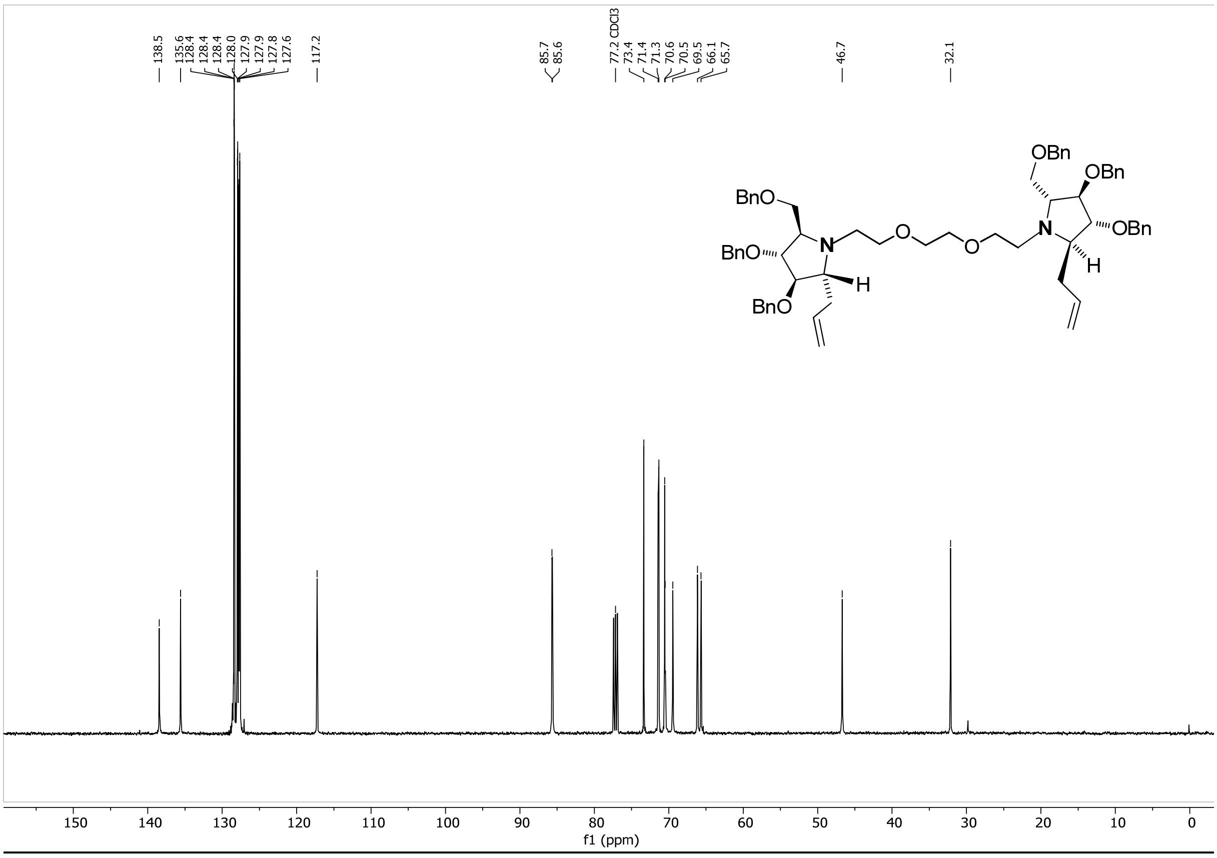

NOESY 2D NMR spectrum of 15d(*R,R*) ($CDCl_3$)

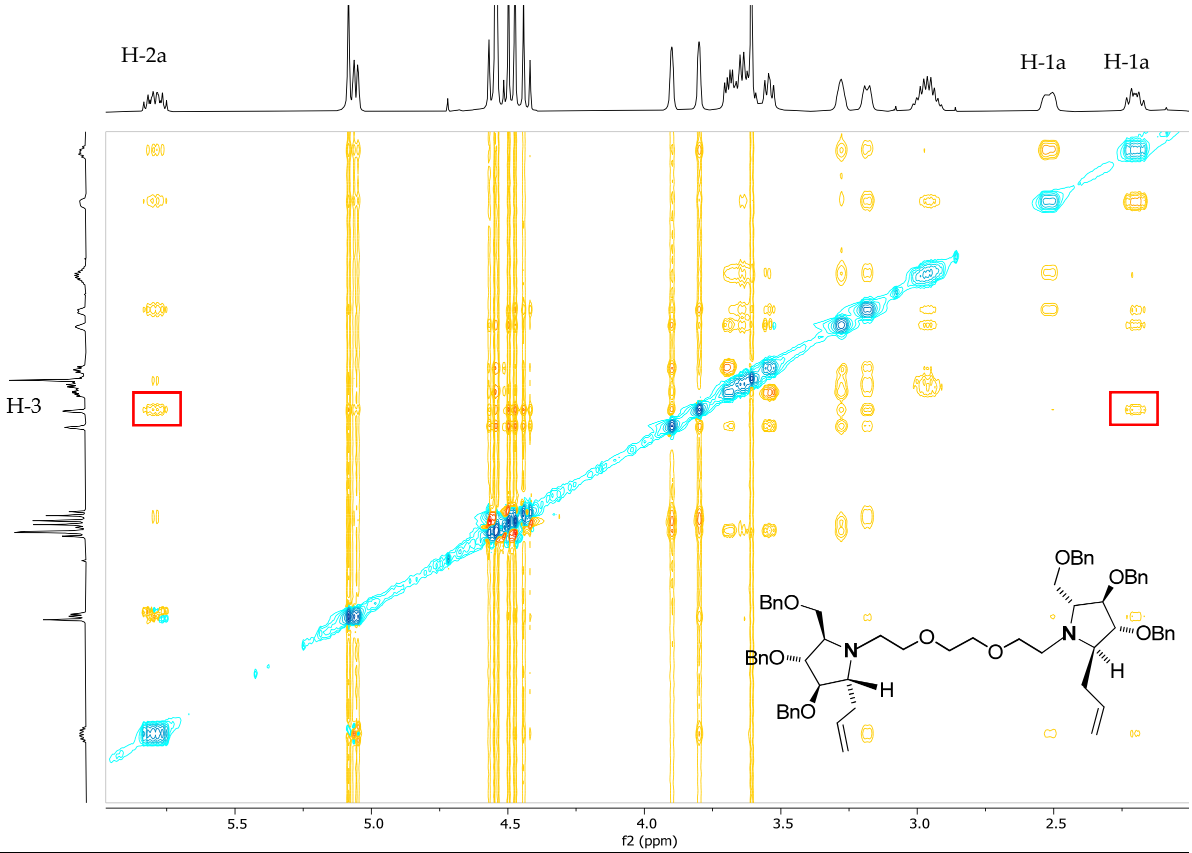

<u>^{1}H-NMR spectrum of 15d(*R*,*S*) (CDCl$_3$)</u>

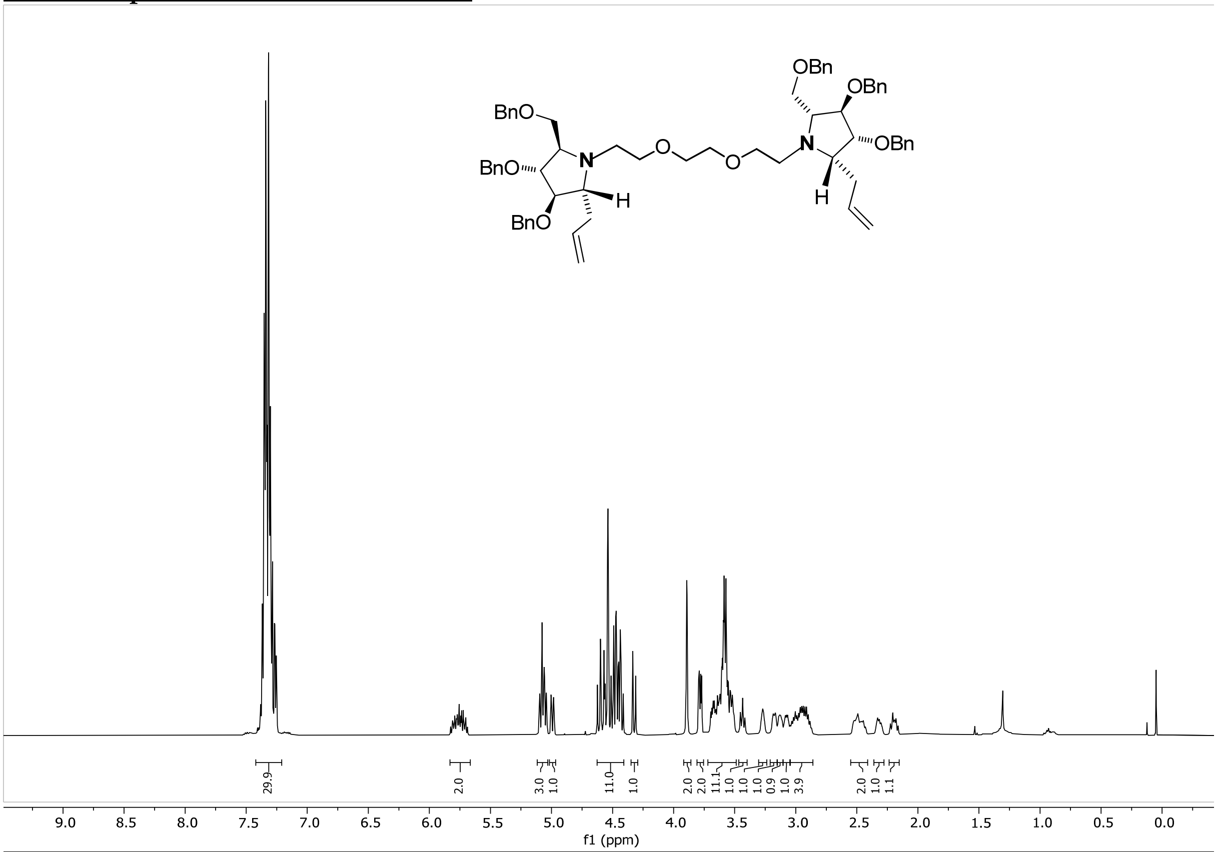

<u>^{13}C-NMR spectrum of 15d(*R*,*S*) (CDCl$_3$)</u>

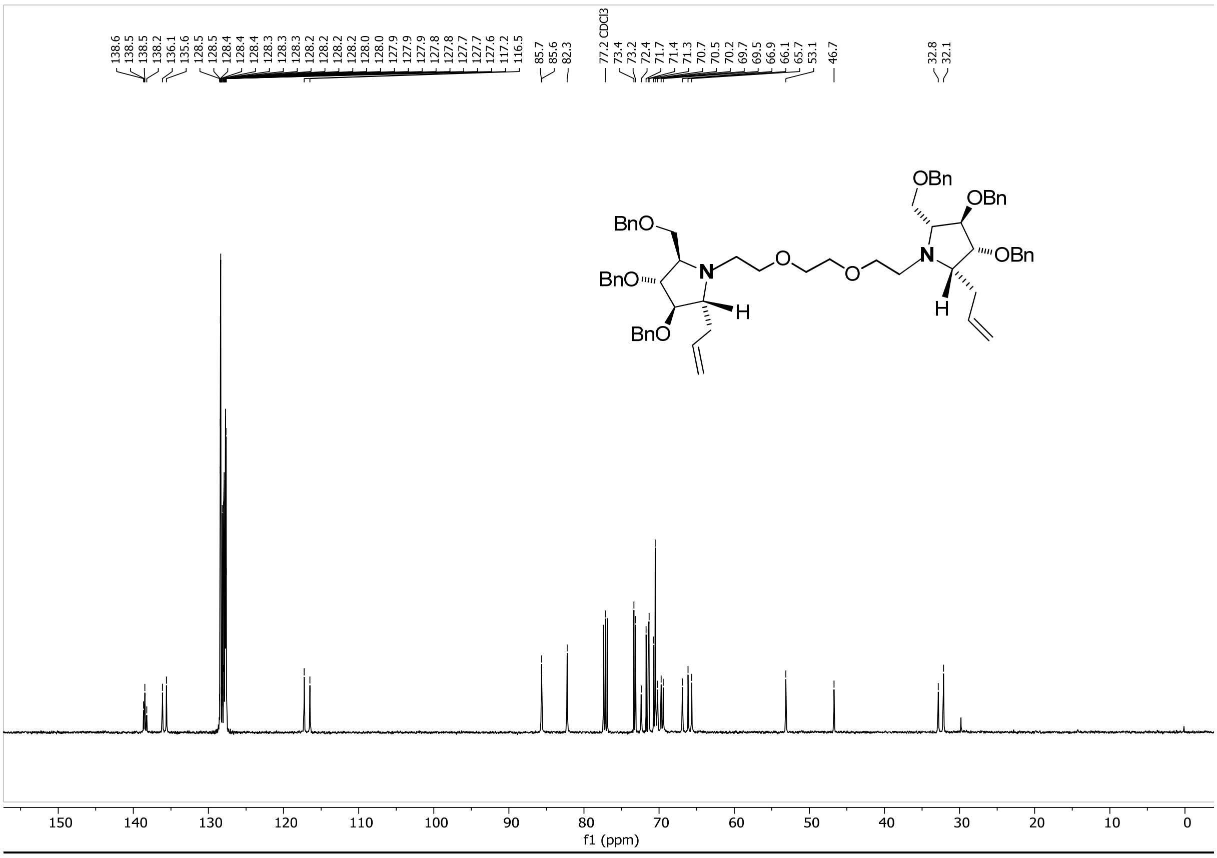

^{1}H-NMR spectrum of 16d(*R*,*R*) (D_2O)

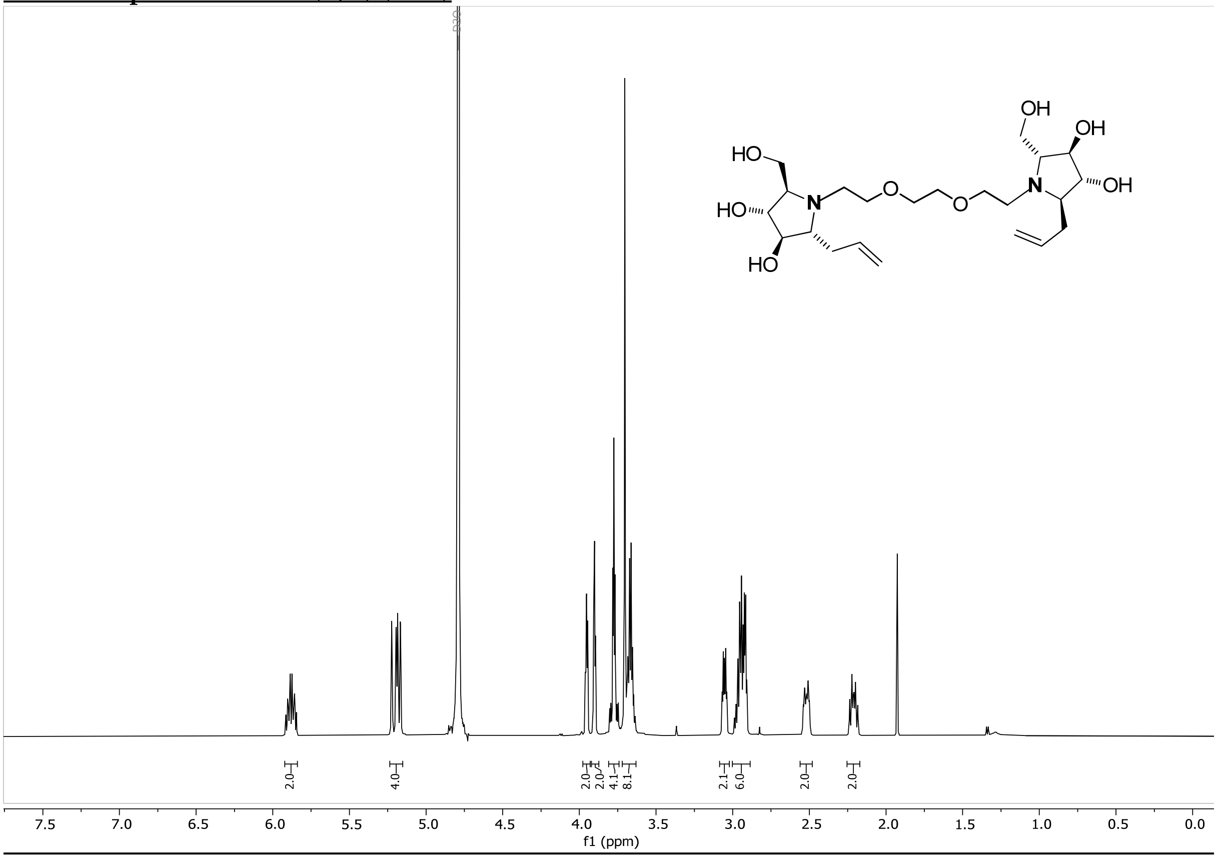

^{13}C-NMR spectrum of 16d(*R*,*R*) (D_2O)

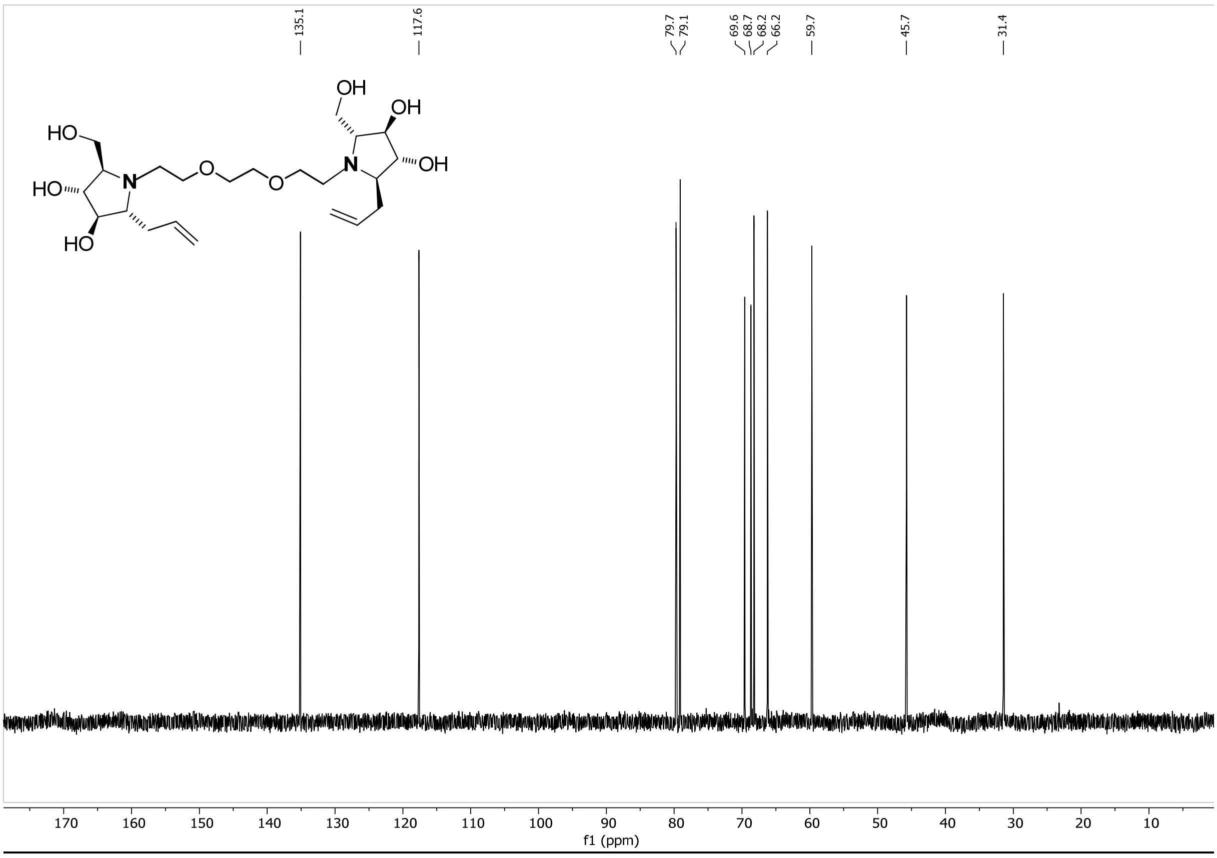

<u>^{1}H-NMR spectrum of 18d ($CDCl_3$)</u>

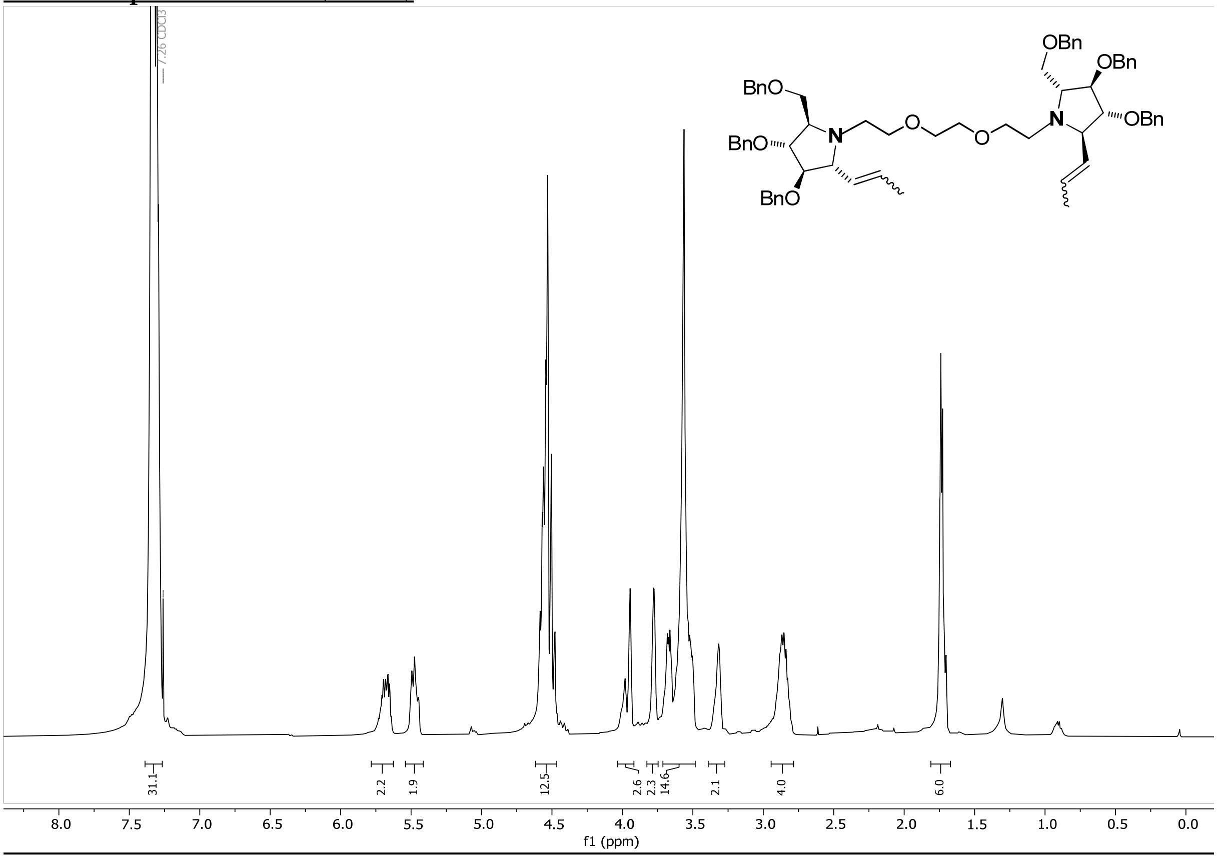

<u>^{13}C-NMR spectrum of 18d ($CDCl_3$)</u>

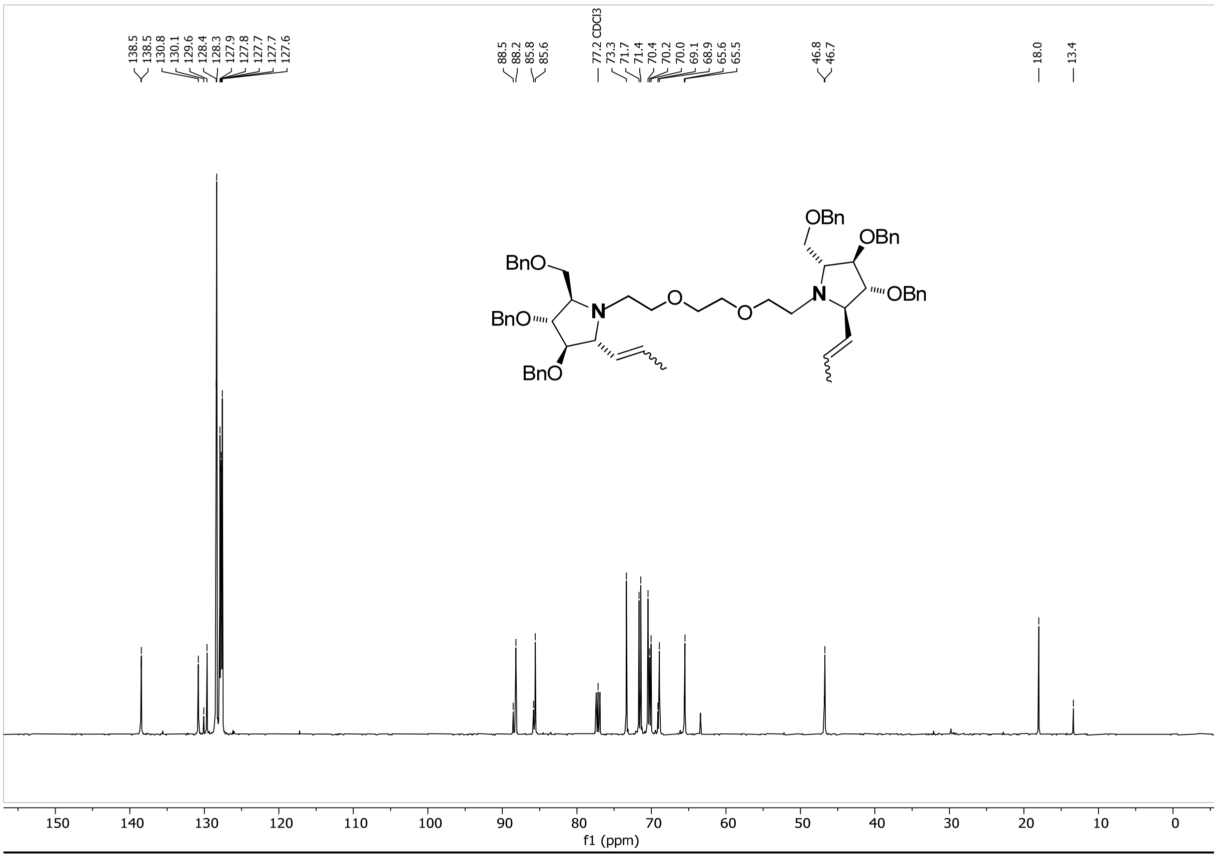

^{1}H-NMR spectrum of 19d(*R*,*R*) and 19d(*R*,*S*) (CD_3OD)

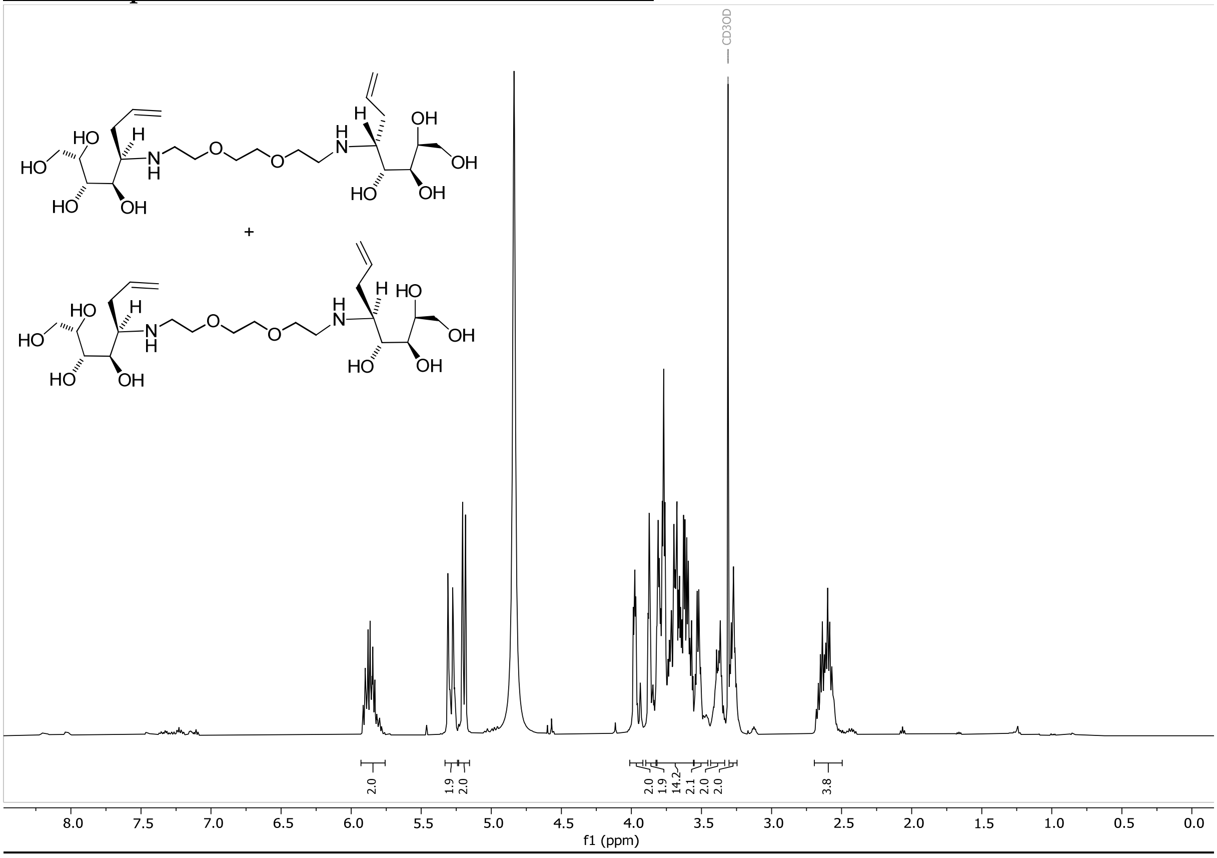

^{13}C-NMR spectrum of 19d(*R*,*R*) and 19d(*R*,*S*) (CD_3OD)

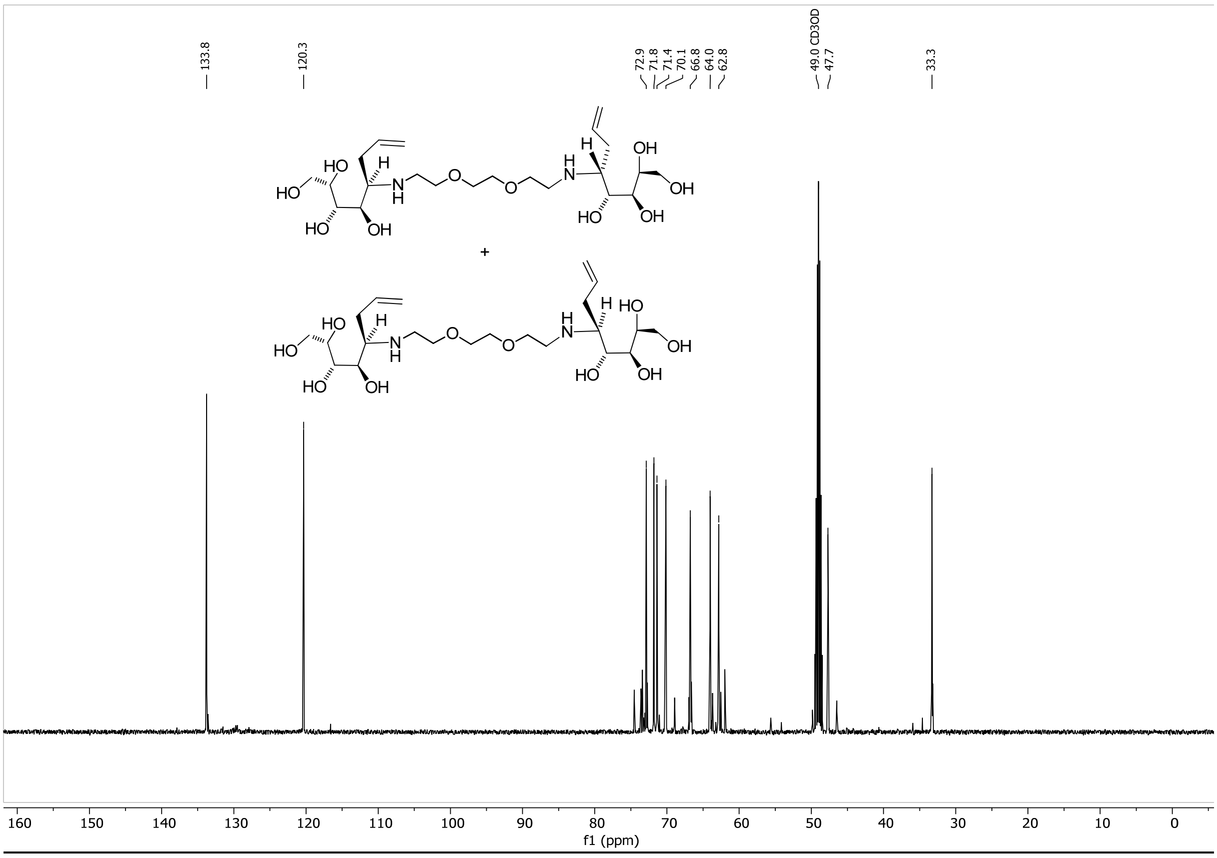

^{1}H-NMR spectrum of 20d (CDCl₃)

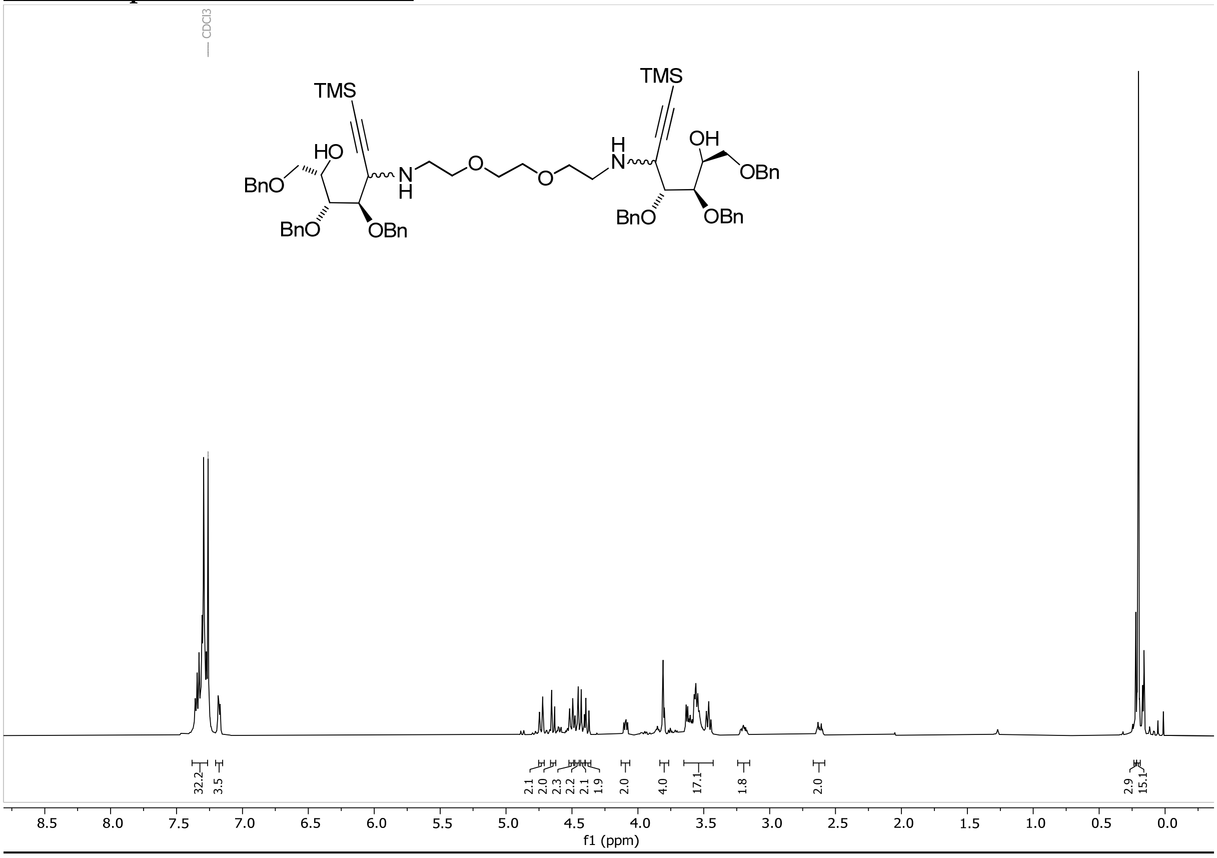

^{13}C-NMR spectrum of 20d (CDCl₃)

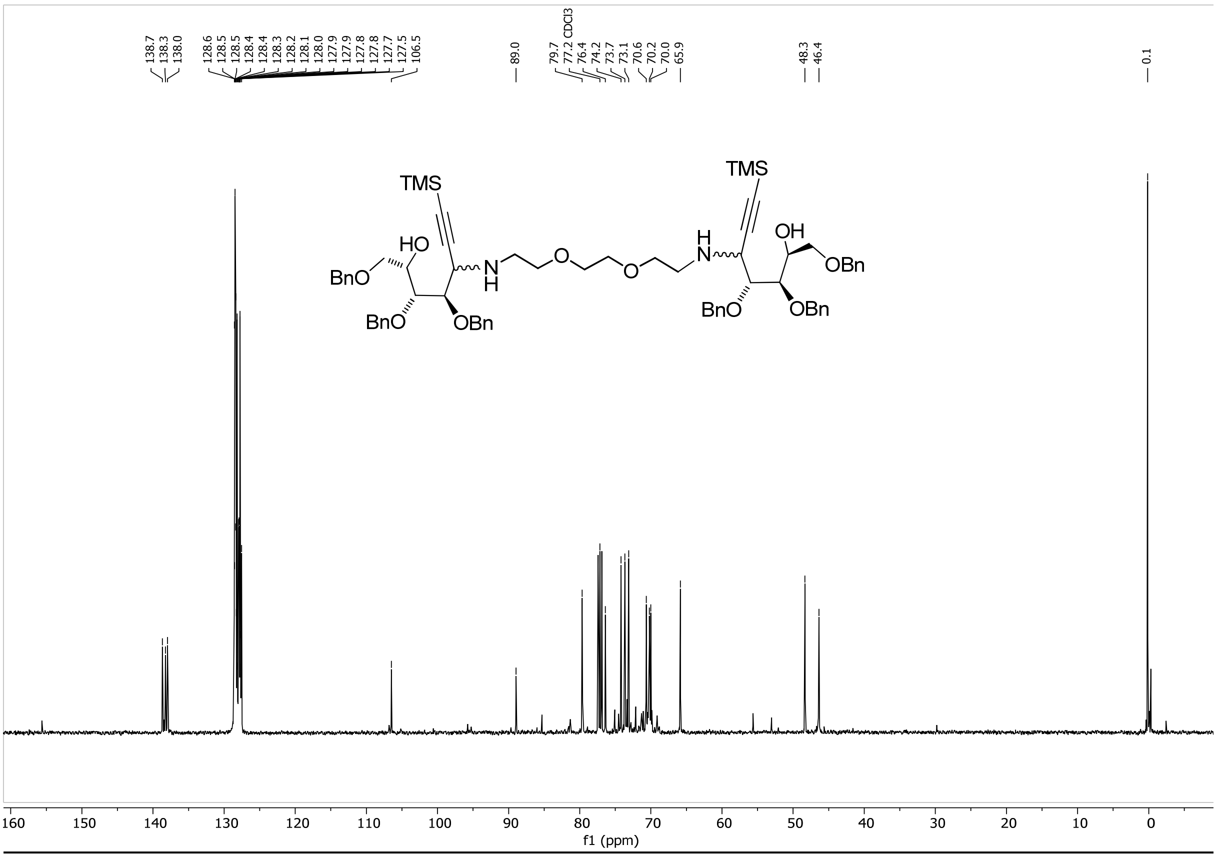

^{1}H-NMR spectrum of 21d (CDCl$_3$)

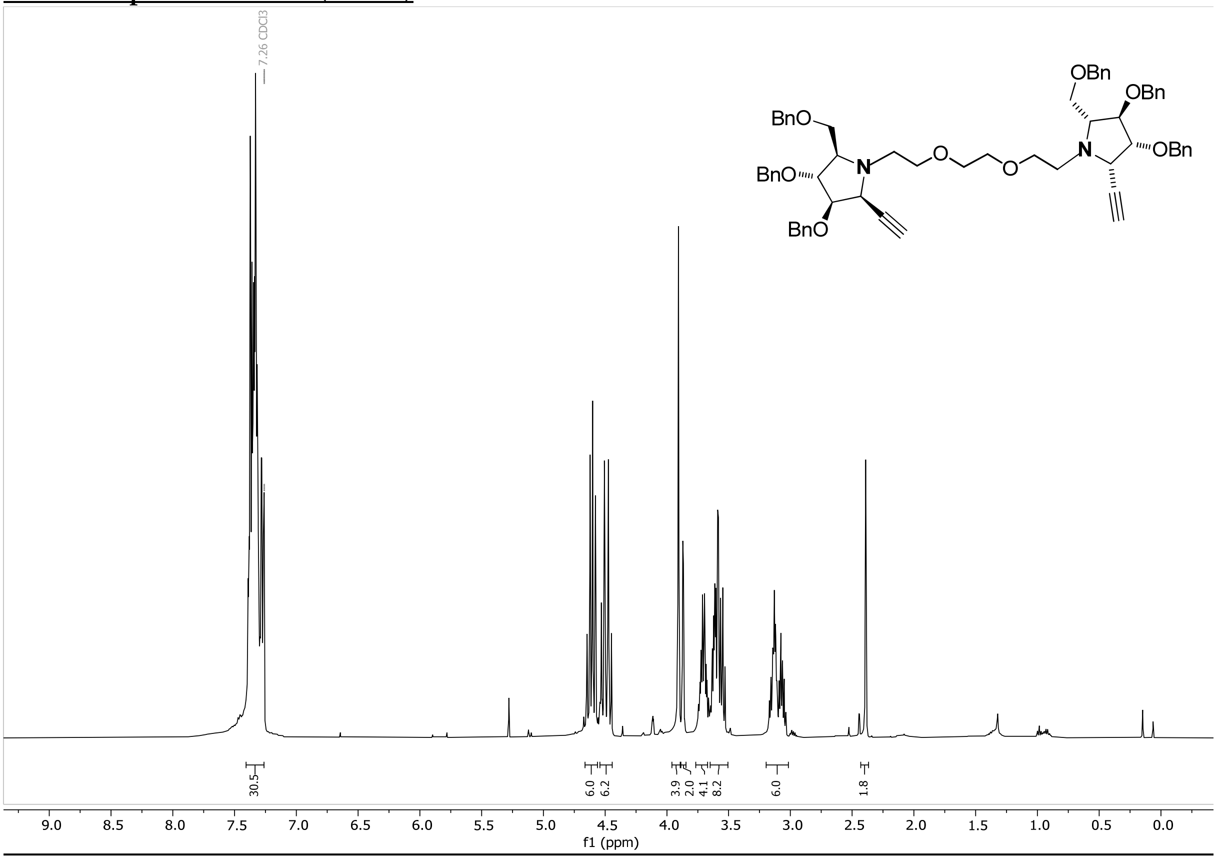

^{13}C-NMR spectrum of 21d (CDCl$_3$)

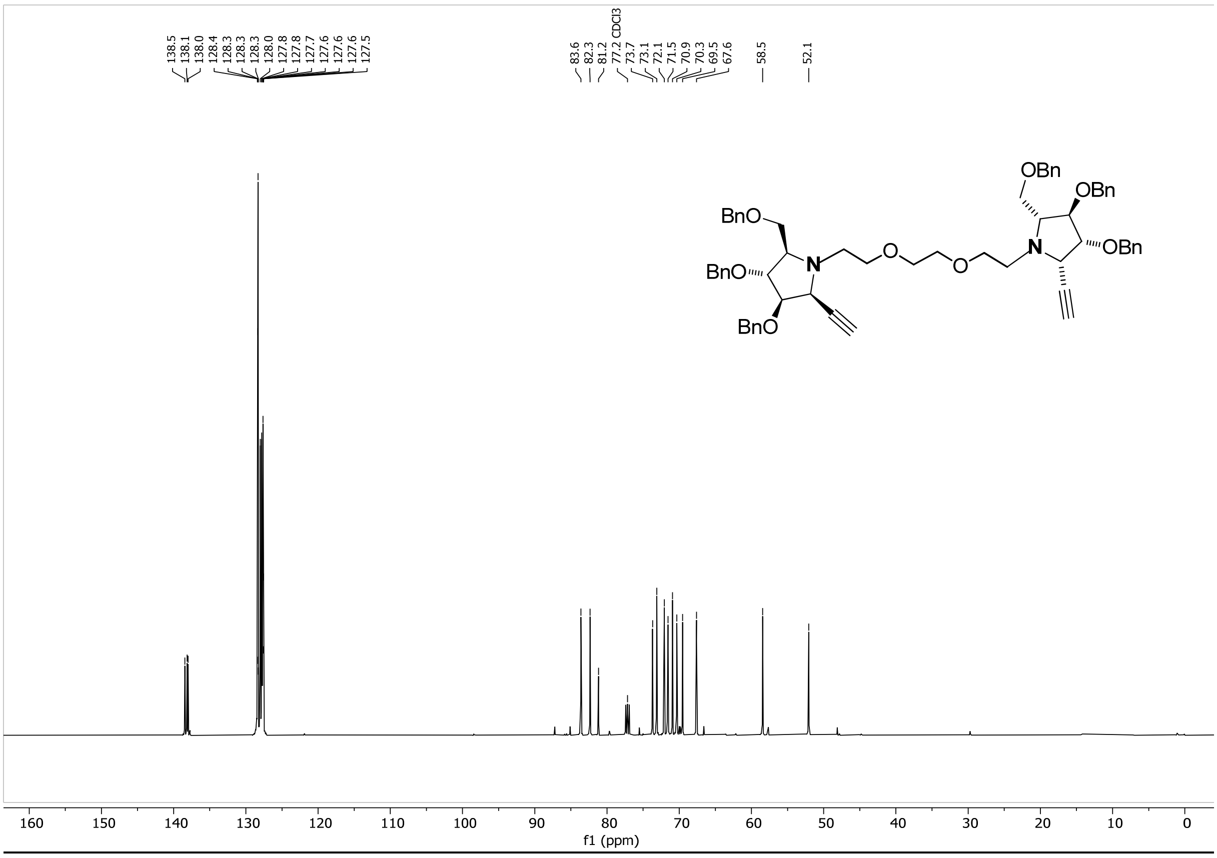

NOESY 2D NMR spectrum of 21d ($CDCl_3$)

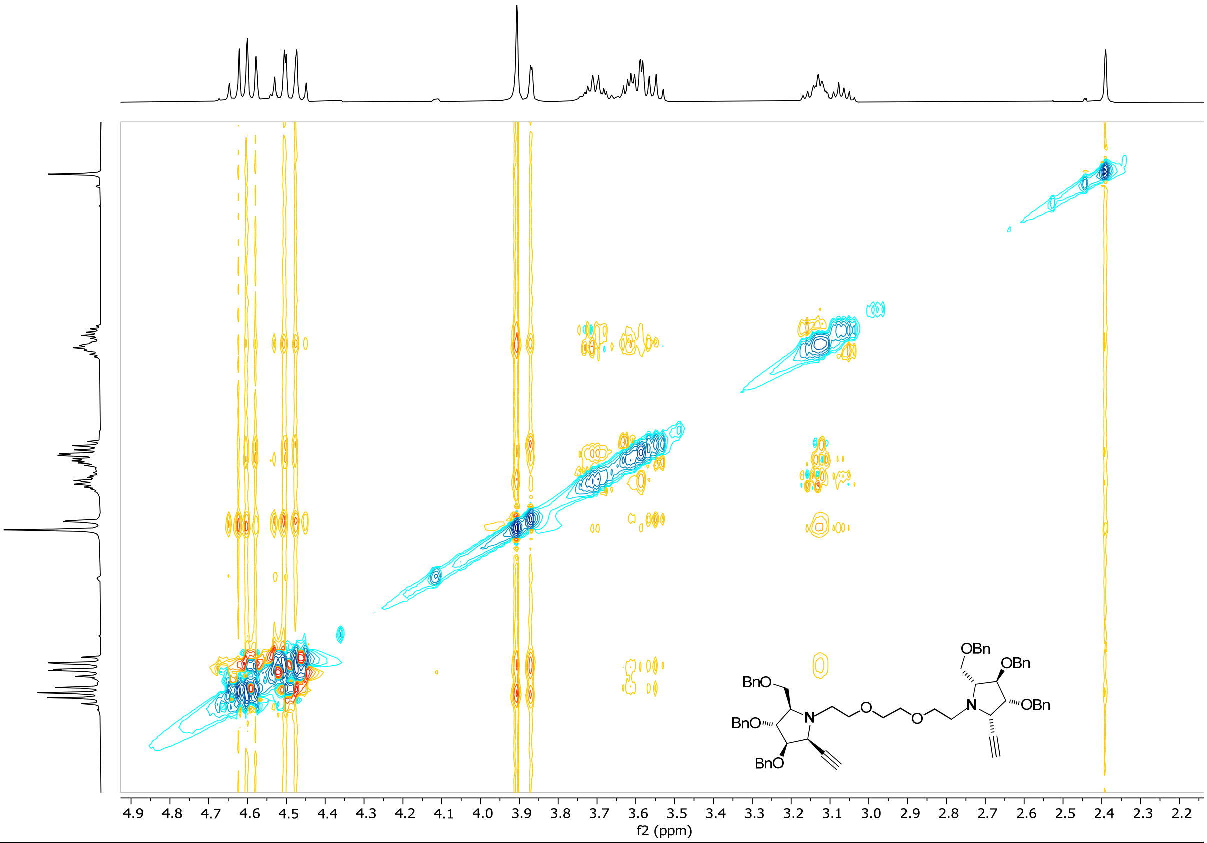

^{1}H-NMR spectrum of 22d ($CDCl_3$)

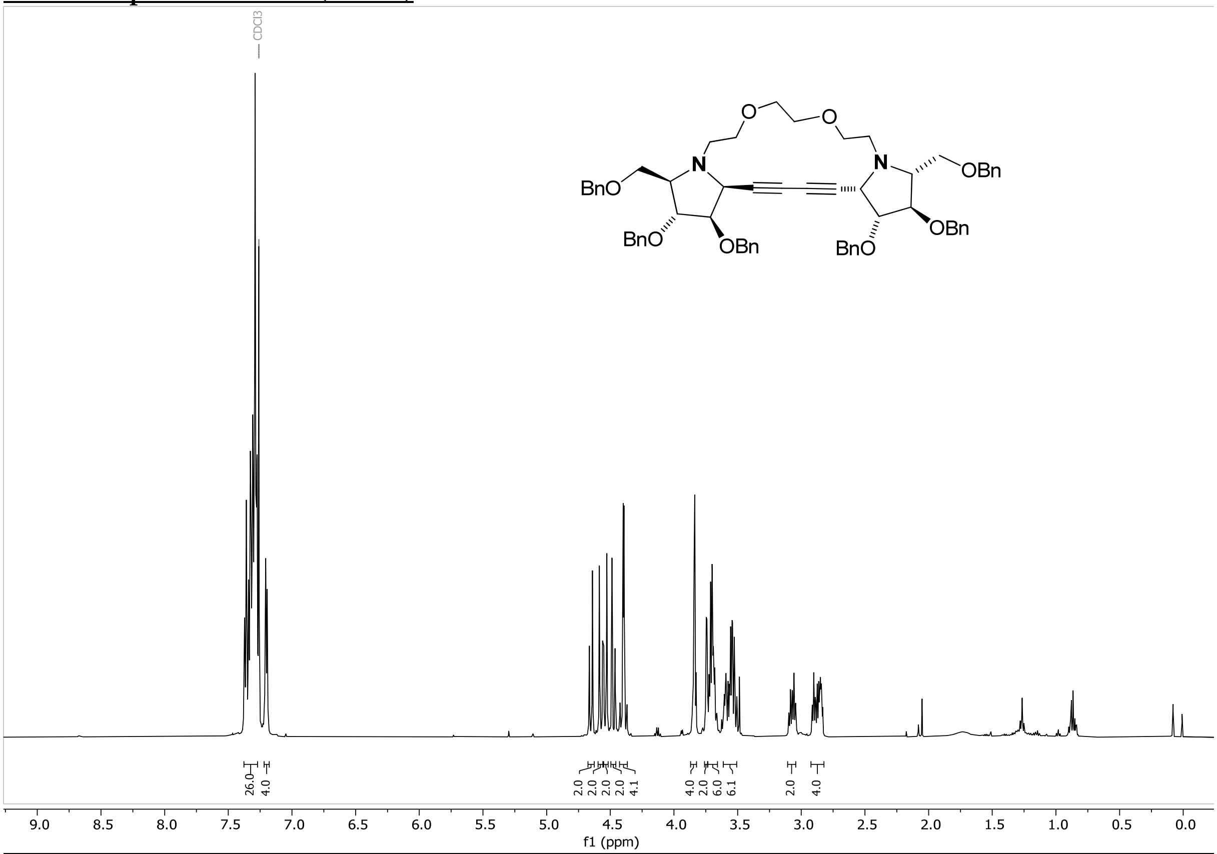

^{13}C-NMR spectrum of 22d ($CDCl_3$)

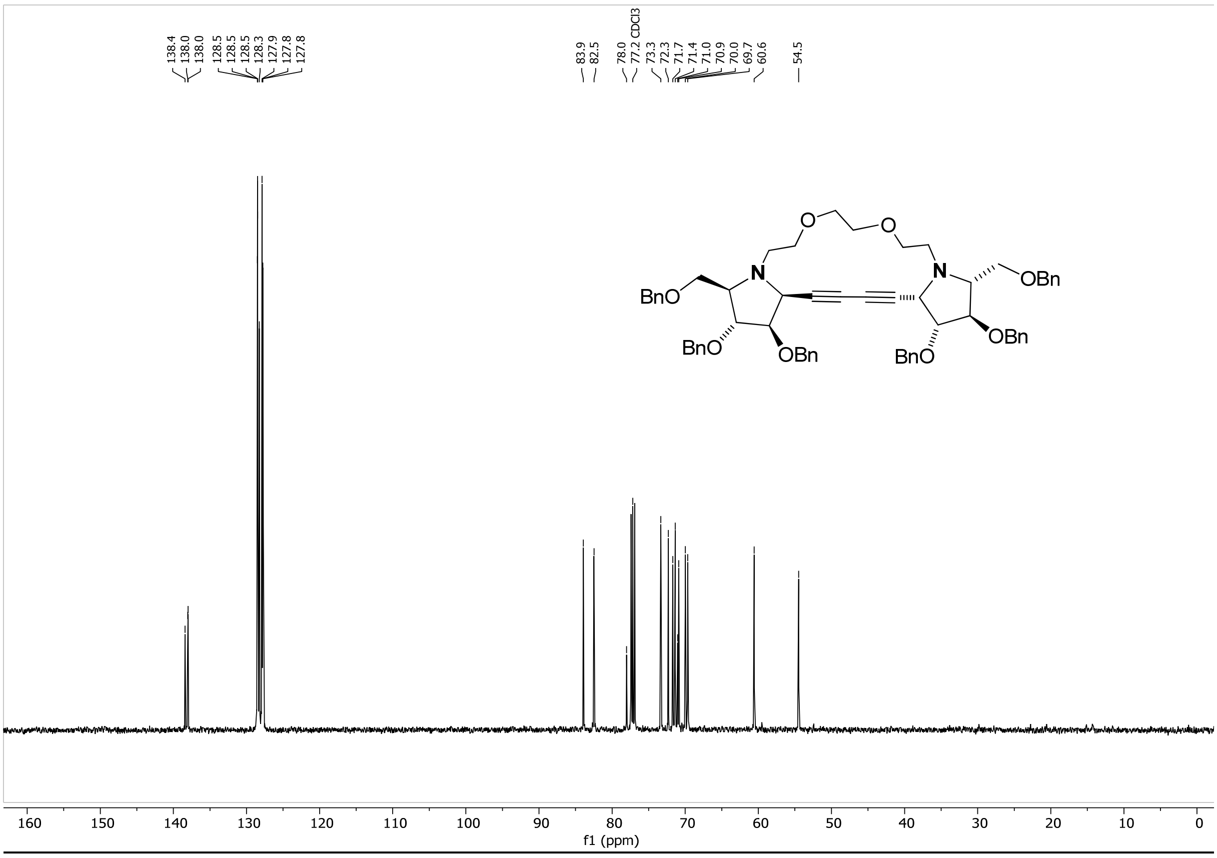

^{1}H-NMR spectra of 19d(*R*,*R*) and 19d(*S*,*S*) (D_2O)

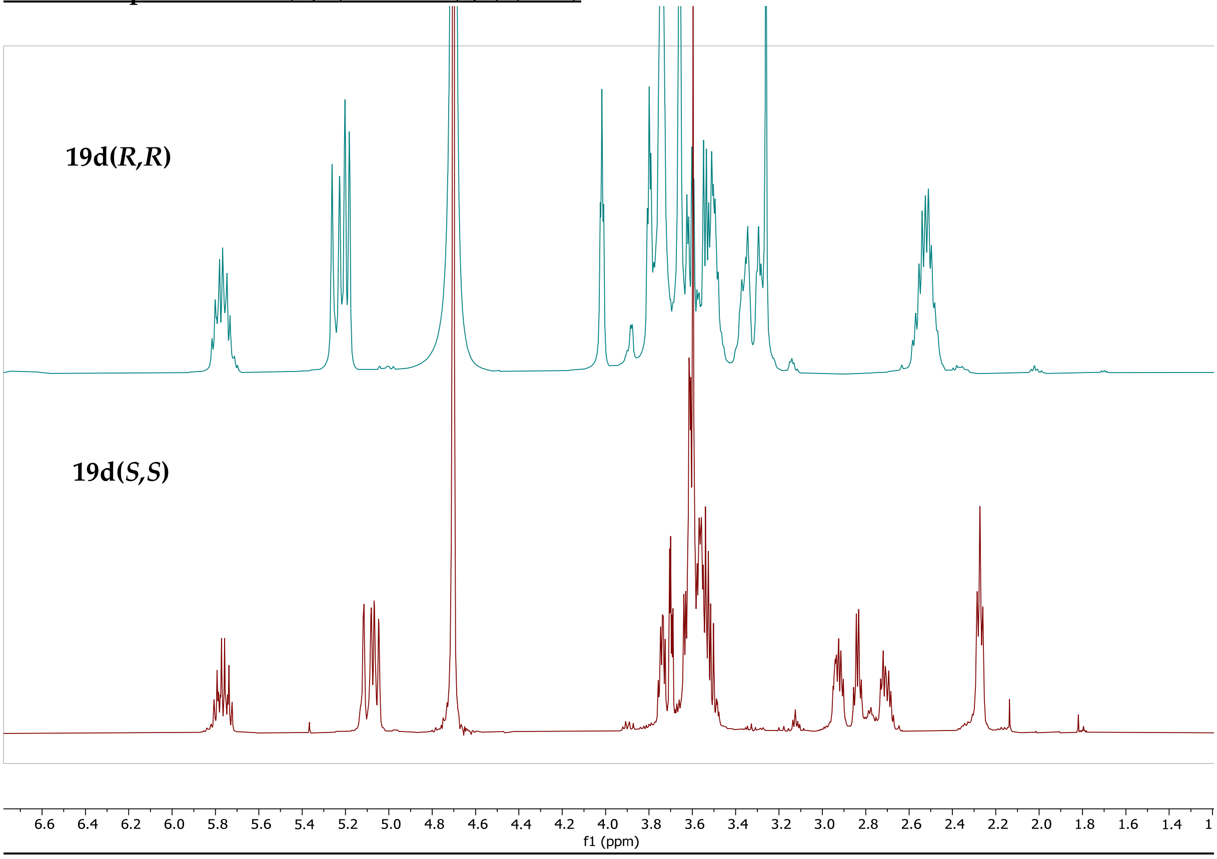

^{13}C-NMR spectra of 19d(*R*,*R*) and 19d(*S*,*S*) (D_2O)

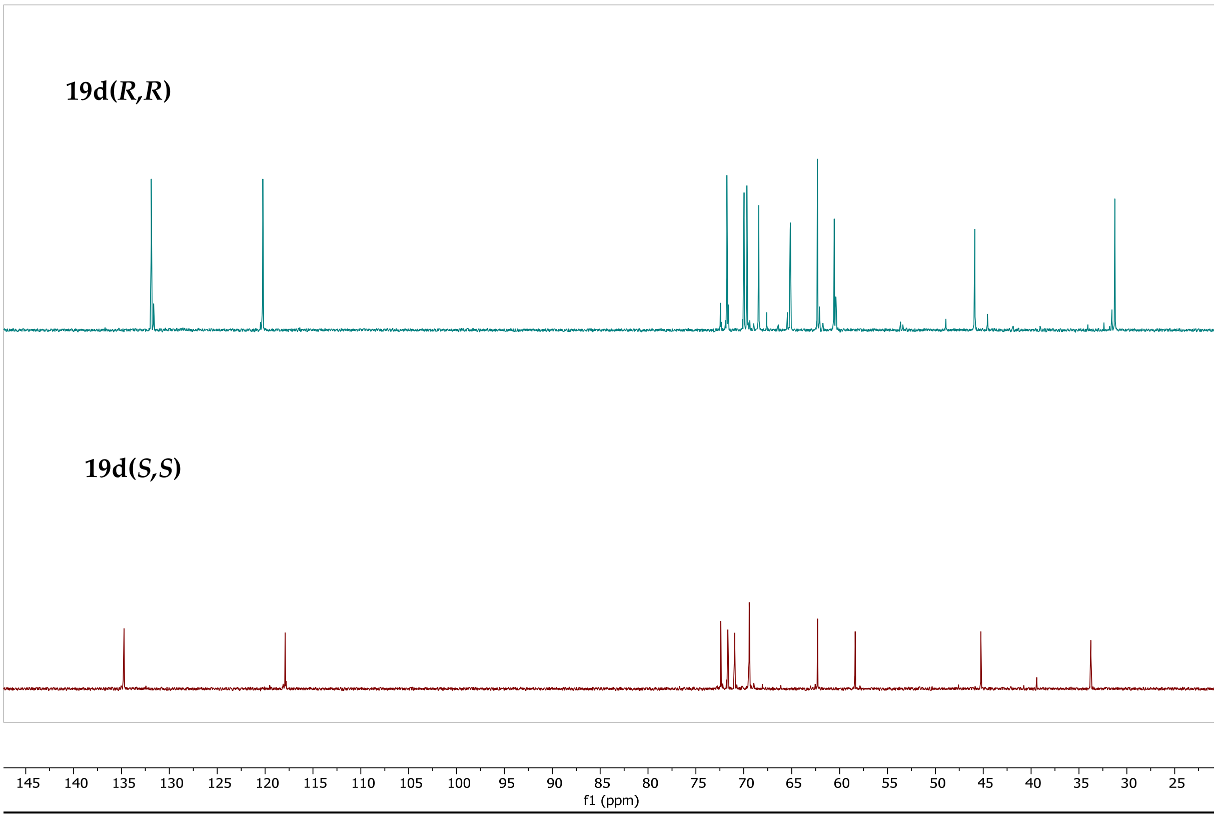
19d(*R*,*R*)
19d(*S*,*S*)
145 140 135 130 125 120 115 110 105 100 95 90 85 80 75 70 65 60 55 50 45 40 35 30 25
f1 (ppm)
